# Conversational Multi-Agentic Network-Driven Disease Module Identification and Drug Repurposing Prediction With ChatDRex: Platform Development Study

**DOI:** 10.2196/93862

**Published:** 2026-11-03

**Authors:** Simon Süwer, Kester Bagemihl, Sylvie Baier, Lucia Dicunta, Markus List, Jan Baumbach, Fernando Miguel Delgado-Chaves, Andreas Maier

**Affiliations:** 1 Institute for Computational Systems Biomedicine, University of Hamburg, Albert-Einstein-Ring 8-10, Hamburg, Hamburg, Germany, +49 40 42838-7635; 2 Data Science of Systems Biology, School of Life Sciences, Technical University of Munich, München, Bavaria, Germany; 3 Data Science in Systems Biology, TUM School of Life Sciences, Technical University of Munich, Freising, Germany; 4 Munich Data Science Institute, Technical University of Munich, , Garching, Germany; 5 Institute for Mathematics and Computer Science, University of Southern Denmark, Odense, Denmark

**Keywords:** drug repurposing, systems medicine, multi-agent systems, reproducible biomedical pipelines, network medicine, biomedical knowledge graphs, large language models, conversational artificial intelligence

## Abstract

**Background:**

Repurposing approved drugs offers a time- and cost-effective alternative to traditional drug development. However, in silico prediction of repurposing candidates is challenging and requires the effective collaboration of specialists in various fields, including pharmacology, medicine, biology, and bioinformatics. Fragmented, specialized algorithms and tools often address only narrow aspects of the overall problem. Heterogeneous, unstructured data landscapes require the expertise of specialized users. Hence, diverse data sources might not integrate smoothly across workflows.

**Objective:**

We aimed to develop an integrated, natural language–driven system that enables domain experts to conduct complex network-based drug repurposing analyses by integrating heterogeneous biomedical data sources and specialized bioinformatics methods within a unified workflow.

**Methods:**

With ChatDRex, we present a conversation-based, multi-agent system that facilitates the execution of complex bioinformatic analyses aiming for network-based drug repurposing prediction. It builds on the integrated systems medicine knowledge graph NeDRexDB. ChatDRex provides natural language access to its extensive biomedical knowledge base. It integrates bioinformatics agents for network analysis, literature mining, and drug repurposing. These are complemented by agents that evaluate functional coherence for in silico validation. Its flexible multi-agent design assigns specific tasks to specialized agents, including query routing, data retrieval, algorithm execution, and result visualization. A dedicated reasoning module keeps the user in the loop and allows for hallucination detection.

**Results:**

ChatDRex operationalizes complex network-based drug repurposing workflows through conversational interaction, integrating biomedical knowledge access with algorithm execution and validation-oriented assessments. Its modular multi-agent architecture enables scalable task specialization across retrieval, analysis, validation, and visualization, while the reasoning module provides transparency and safeguards against unreliable outputs.

**Conclusions:**

By enabling researchers with limited computational expertise to control complex analyses with natural language, ChatDRex could democratize access to bioinformatics as an important resource for drug repurposing. It could enable clinical experts to generate hypotheses and explore drug repurposing opportunities, ultimately accelerating the discovery of novel therapies and advancing personalized medicine and translational research.

## Introduction

The development of novel pharmaceuticals requires substantial time and financial investments. Additionally, it is often accompanied by a high risk of failure in the late stages of clinical trials [1-3]. To address these challenges, there is an increasing emphasis on repurposing already formulated drugs for novel therapeutic applications. This approach leverages comprehensive pharmacological, clinical, or genomic data to identify potential new indications for existing drugs [1,2]. This approach not only significantly accelerates the development process but also reduces the risk of clinical failures, as the safety and tolerability of the substances are already established [1,2].

In particular, systems medicine provides a conceptual framework for understanding disease as a complex, multiscale process that arises from interactions across molecular, cellular, and clinical levels [4]. Network medicine operationalizes this paradigm by translating system-level views into explicit computational models, which are typically represented as biological and pharmacological networks [5]. Researchers have found these network-based methodologies to be helpful in identifying promising drug repurposing candidates, thereby speeding up the transition from discovery to clinical application [6,7]. Since networks capture the relationships among genes, drugs, proteins, and diseases, they enable the identification of novel indications for existing pharmaceutical agents [8]. This type of network-based prediction of drug repurposing candidates has already been demonstrated in the context of Alzheimer disease and colorectal cancer [9-12].

A generic workflow for network-driven drug repurposing was implemented, demonstrated, and evaluated for biological adequacy within the NeDRex (Network-based Drug Repurposing and Exploration) project [7], which was later expanded and complemented by, for example, NeDRex-Web [13] and Drugst.One [6]. Central to this methodology are concepts such as *seed genes*—typically genes known to be implicated in a specific disease—which serve as the starting point for drug repurposing queries. *Disease modules* are clusters of interconnected genes driving the manifestation of a disease phenotype. For example, in Huntington disease, the *HTT* gene is the main disease-causing gene, and the associated network consisting of genes involved in neuronal damage forms the Huntington disease module. *Disease module identification* algorithms, such as DIAMOnD (Disease Module Detection), are used to extract these modules from complex biological networks [14]. Once a disease module is identified, *drug prioritization* algorithms rank drugs that target the identified module, thereby allowing researchers to focus on the most promising candidates for repurposing [15]. These algorithms utilize network topology, often through proximity measures, to determine the association between a drug target and the disease module, providing insights into the drug’s potential efficacy [15].

Nevertheless, the field faces considerable challenges due to the substantial and intricate nature of biomedical information that must be examined to identify drug candidates with potential for novel indications. The NeDRex platform, primarily composed of NeDRexDB, which includes the NeDRex knowledge graph (KG) and the NeDRex API, was developed to address the identified challenges by providing a comprehensive suite of tools for network-based drug repurposing [7]. Utilizing these advanced tools requires a substantial level of bioinformatics expertise or dedicated training, which poses a significant hurdle for many clinicians despite their valuable pharmacological or disease-specific expertise [16]. Consequently, there is a pressing need for more accessible and user-friendly interfaces to bridge the gap between complex bioinformatics tools and the researchers and clinicians who could benefit most from their insights, ultimately accelerating the development of new treatments and personalized medicine approaches [17,18].

Large language models (LLMs) represent a transformative advance in this regard, offering unprecedented capabilities in natural language processing, including question answering (QA) and even medical reasoning [19,20]. The success of these models can be attributed to their extensive training on vast quantities of text data, which has enabled them to comprehend and generate coherent natural language [20]. These models have revolutionized biomedical data analysis by providing powerful text-understanding capabilities [21-23]. However, their generalized nature often limits their effectiveness in specialized biomedical domains [24,25]. Additionally, it has been observed that LLMs tend to generate hallucinations, producing plausible yet erroneous or fallacious information [26,27]. This phenomenon poses a significant challenge in high-risk domains such as biomedicine. The unreliability of LLMs underscores the necessity for domain-specific adaptations and rigorous validation when using LLMs in biomedical research and clinical decision-making processes [26,27]. To overcome this limitation, Retrieval-Augmented Generation (RAG) and In-Context Learning (ICL) techniques promise to enhance LLMs by incorporating external knowledge retrieval, thereby improving the accuracy and relevance of responses in specialized fields [20,28]. LLMs can further be organized as agents, which are autonomous entities designed to perform specific tasks. These agents operate without supervision, allowing them to pursue individual objectives and implement different strategies, including analyzing external data [29]. To execute a task, LLM agents possess a memory that enables them to acquire knowledge from prior interactions and to preserve context over extended periods of time [29]. In particular, in the area of drug repurposing, LLM agents can work toward the goal of providing accurate biomedical insights by autonomously accessing data services, retrieving relevant information, and iteratively refining their responses based on user feedback [30]. Through interagent communication, these specialized agents interact with databases, execute algorithms, and generate human-understandable explanations. This collaborative approach bridges the gap between complex computational tools and the needs of researchers and clinicians.

Building on these advancements in LLMs and agent technology, we introduce ChatDRex, an intuitive conversational agent designed to assist with identifying disease modules and repurposing drugs [31]. ChatDRex leverages a multi-agent architecture to provide a user-friendly interface for essential functions of the NeDRex platform, automating complex analyses and enhancing result visualization (Figure 1). The objective of ChatDRex is to democratize access to computational drug repurposing tools and facilitate the identification of potential treatment options for existing diseases by both researchers and clinicians. Additionally, ChatDRex incorporates network-driven drug repurposing strategies into an interactive, conversation-based system. This system is designed to help researchers and clinicians identify potential treatment options for existing diseases.

**Figure 1.**
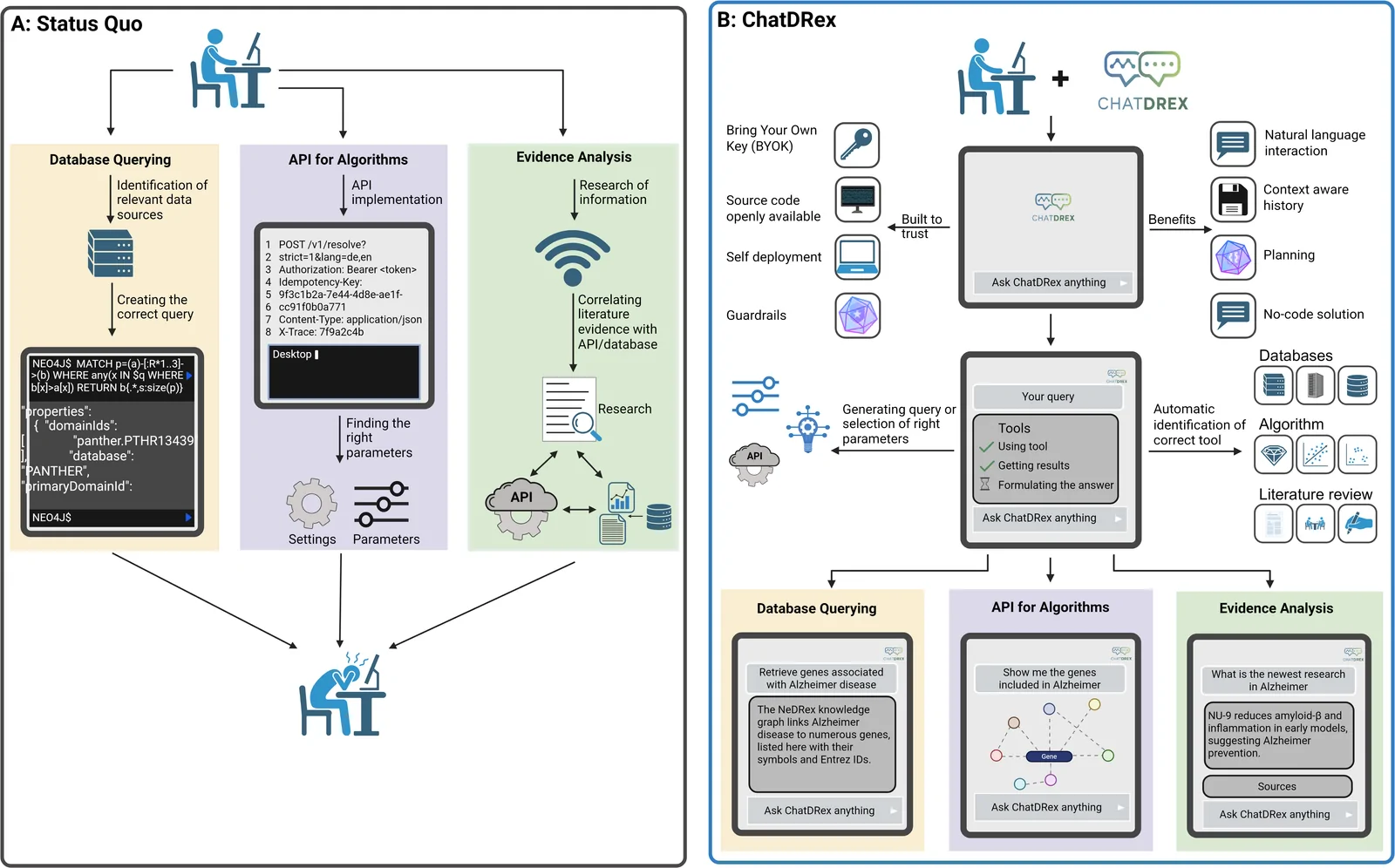
ChatDRex overview. The figure compares the traditional manual workflow for biomedical analyses (A) with the ChatDRex approach (B). Presently, users are required to formulate database queries, configure algorithmic APIs, and manually correlate the results with literature references. ChatDRex integrates these steps into a single, natural language–based workflow. ChatDRex automatically selects tools and generates the appropriate parameters. This capability allows ChatDRex to integrate relevant information from databases, algorithms, and the literature into a cohesive result. Figure created in bioRender [32]. NeDRex: Network-based Drug Repurposing and Exploration.

## Methods

### ChatDRex

ChatDRex streamlines complex, network-based drug repurposing analyses with a conversational interface. This approach is designed to address potential biases and hallucinations in LLMs by integrating directly with validated biomedical databases and specialized bioinformatics tools. It ensures data accuracy and enables sophisticated analyses without requiring specialized computational expertise.

The implemented multi-agent architecture is inspired by the chain-of-thought (CoT) paradigm [33] but adapted to the modularity of complex systems. In contrast to the original CoT, in which a single model sequentially externalizes the intermediate steps of reasoning [34], our approach delegates reasoning to a set of specialized agents, each of which has a clearly defined role in the drug repurposing workflow (Figure 2). A central planning agent functions as a meta-controller, breaking down user requests into subtasks, iteratively assigning them to the appropriate expert agents, and merging their results into a coherent outcome [35]. Consequently, the architecture enables task planning and orchestration, allowing each agent to focus on a small subtask using CoT [34].

**Figure 2.**
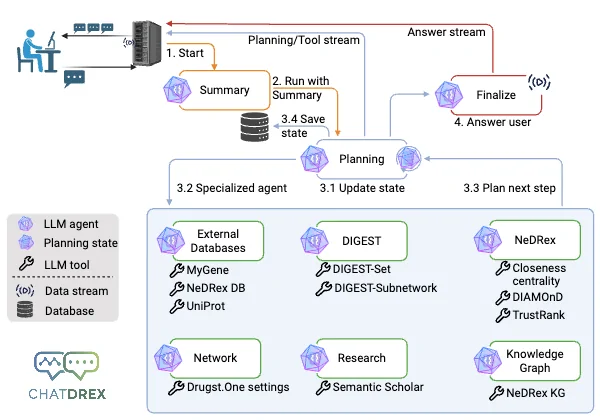
ChatDRex multi-agent architecture. The workflow begins when a user sends a prompt (1). The summary agent synthesizes the current conversation and task context into a concise overview (2). ChatDRex is orchestrated by a central planning agent that routes the user prompt and the summary to specialized agents according to required tasks (3). The DIGEST (In Silico Validation of Disease and Gene Sets, Clusterings or Subnetworks) agent performs functional coherence and enrichment analyses [36]. The knowledge graph (KG) agent provides access to the NeDRex (Network-based Drug Repurposing and Exploration) KG [7]. Our NeDRex agent enables network-based drug repurposing analyses via the NeDRex API [7]. The research agent conducts literature retrieval, while an external database agent enriches queries with additional external knowledge sources. The network agent controls and adapts the visualization of interaction networks using a Drugst.One-powered graph interface [6]. Finally, the finalize agent integrates and synthesizes the outputs of all agents into a coherent response (4). Figure created in bioRender [32]. DIAMOnD: Disease Module Detection; LLM: large language model.

### ChatDRex Agent Architecture

ChatDRex is a multi-agent system (Figure 2), where all agents are constructed based on the same architectural principles (Figure 3). Each agent integrates a unified control loop consisting of few-shot prompting, which is part of ICL, short-term and long-term memory access, internal planning routines, and external tool integration. Our lightweight summary agent maintains contextual continuity by compressing prior interaction states (Section S1 in Multimedia Appendix 1). This reduces token usage and enables controlled memory resets. Prompt-injection safeguards are applied at this stage, which is particularly relevant given that LLMs are systematically vulnerable to jailbreaks and instruction-overriding attacks [37-39]. In addition, a sanity check is performed here to verify, based on user feedback, whether the LLM has correctly understood the request (details and an evaluation of the input guardrails can be found in Multimedia Appendix 2). According to the available summary and the new user prompt, the coordination of tasks is the responsibility of a planning agent. This agent uses an LLM-based decision process to dynamically direct user input to the most suitable specialized agents (Section S2 in Multimedia Appendix 1). The selection of agents is formulated as a multistep problem, ensuring efficient task decomposition and specialization [35]. If biological validation or interpretation is requested by the user, this is provided by the DIGEST (In Silico Validation of Disease and Gene Sets, Clusterings or Subnetworks) agent (more detailed algorithm descriptions can be found in Section S3 in Multimedia Appendix 1). It uses DIGEST to evaluate the functional coherence of gene sets and disease-associated subnetworks. Conventional enrichment approaches, which enumerate overrepresented terms, are contrasted with DIGEST, which quantifies internal functional coherence through comparison with randomized reference sets. This process provides empirical significance estimates [36]. Within ChatDRex, DIGEST is used to assess the biological plausibility of disease modules derived from network analyses, as well as user-provided gene sets in the absence of network context.

**Figure 3.**
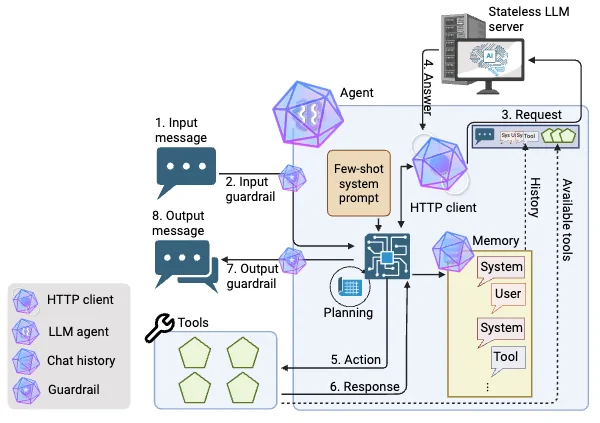
ChatDRex agent architecture*.* The incoming user prompt is enriched by a few-shot system prompt together with relevant memory contents, thereby documenting previous interactions among the user, system, and invoked tools in a structured form (1). This context package is then transmitted to a stateless large language model (LLM) server (open source hosted or proprietary; 3), which processes the request independently of prior states. The server’s response is subsequently evaluated by an interpretation module that decides whether immediate feedback can be returned to the user or whether additional tools need to be engaged [40,41] (4-8). When tools are invoked, their outputs are written back into the respective agent’s memory (5-6), thus closing the chain-of-thought [34] (planning). In addition, ChatDRex can incorporate optional safeguard layers. Input guardrails mitigate prompt injection risks before queries reach the LLM (2), while output guardrails constrain generated responses (6) to domain-specific requirements and filter potentially hallucinatory content (7) [42]. These safeguards are not fixed components of every agent but can be activated depending on the task. This selective integration allows the system to balance robustness against computational efficiency in a fine-grained manner. Figure created in bioRender [32].

Network-driven analyses are conducted by the NeDRex agent, which provides algorithmic access to the integrated NeDRex KG (more detailed algorithm descriptions can be found in Section S4 in Multimedia Appendix 1). This agent orchestrates established network-medicine tools, including DIAMOnD for disease module expansion from seed genes (Section S4.1 in Multimedia Appendix 1) [15]. TrustRank is a methodology that facilitates the prioritization of drug targets based on their proximity to trusted disease nodes (Section S4.2 in Multimedia Appendix 1) [43]. Closeness centrality is used to rank candidates according to their global network distances (Section S4.3 in Multimedia Appendix 1) [7]. The integration of these tools facilitates the systematic identification of disease-relevant subnetworks and candidate therapeutics within a unified interaction graph, thereby constituting the computational core of ChatDRex’s network-based drug repurposing capabilities. The KG agent is responsible for the structured knowledge retrieval process (Section S5 in Multimedia Appendix 1). This agent translates natural language queries into schema-constrained graph queries over the NeDRex KG (Figure 4). The agent enhances its resilience, traceability, and resistance to hallucinations by integrating semantic query decomposition, embedding-based node matching, and deterministic Cypher query generation. Additionally, it uses fallback strategies that are guided by graph-based retrieval.

In order to achieve this, user queries are first semantically mapped to the fixed schema of the NeDRex KG and decomposed into a list of typed subquestions, rather than an explicit question graph as used in prior work [44]. Each subquestion is processed via an LLM call. This call identifies and annotates the corresponding KG node type, generates the query, and determines whether subsequent filtering is required. Subquestions that have been filtered are enriched using an embedding-based similarity search with the top 5 most similar KG nodes. Based on this representation, the agent generates Cypher queries deterministically for execution on the Neo4j-backed NeDRex KG. Query generation is guided by the known NeDRex schema and predefined type and edge constraints, reducing hallucinations and improving traceability [45,46]. The prior node enrichment enables robust filtering using resolved KG identifiers, which mitigates errors caused by spelling variations or lexical mismatches. To prevent over-constraining queries, filtering is applied only to elements that are explicitly marked. If Cypher query generation fails after a fixed number of retries, the agent switches to a GraphRAG-based strategy, in which graph-guided retrieval results are condensed and given to the LLM to generate a response [47]. In instances where NeDRex knowledge is inadequate, the research agent initiates a literature-based retrieval process by decomposing the query into focused subquestions and querying external scholarly resources (Semantic Scholar; Section S6 in Multimedia Appendix 1) [35,48]. This process serves to complement the KG with the most recent scientific evidence [35,48]. Consequently, the finalize agent synthesizes intermediate results into a coherent, citation-aware response (Section S7 in Multimedia Appendix 1) [49,50]. A secondary verification step evaluates generated content against the planning state, which contains the raw results from the other agents to mitigate hallucinations and ensure factual consistency [51,52]. If a graphical representation is required, the user has the option of controlling the Drugst.One graph representation via the network agent [6].

**Figure 4.**
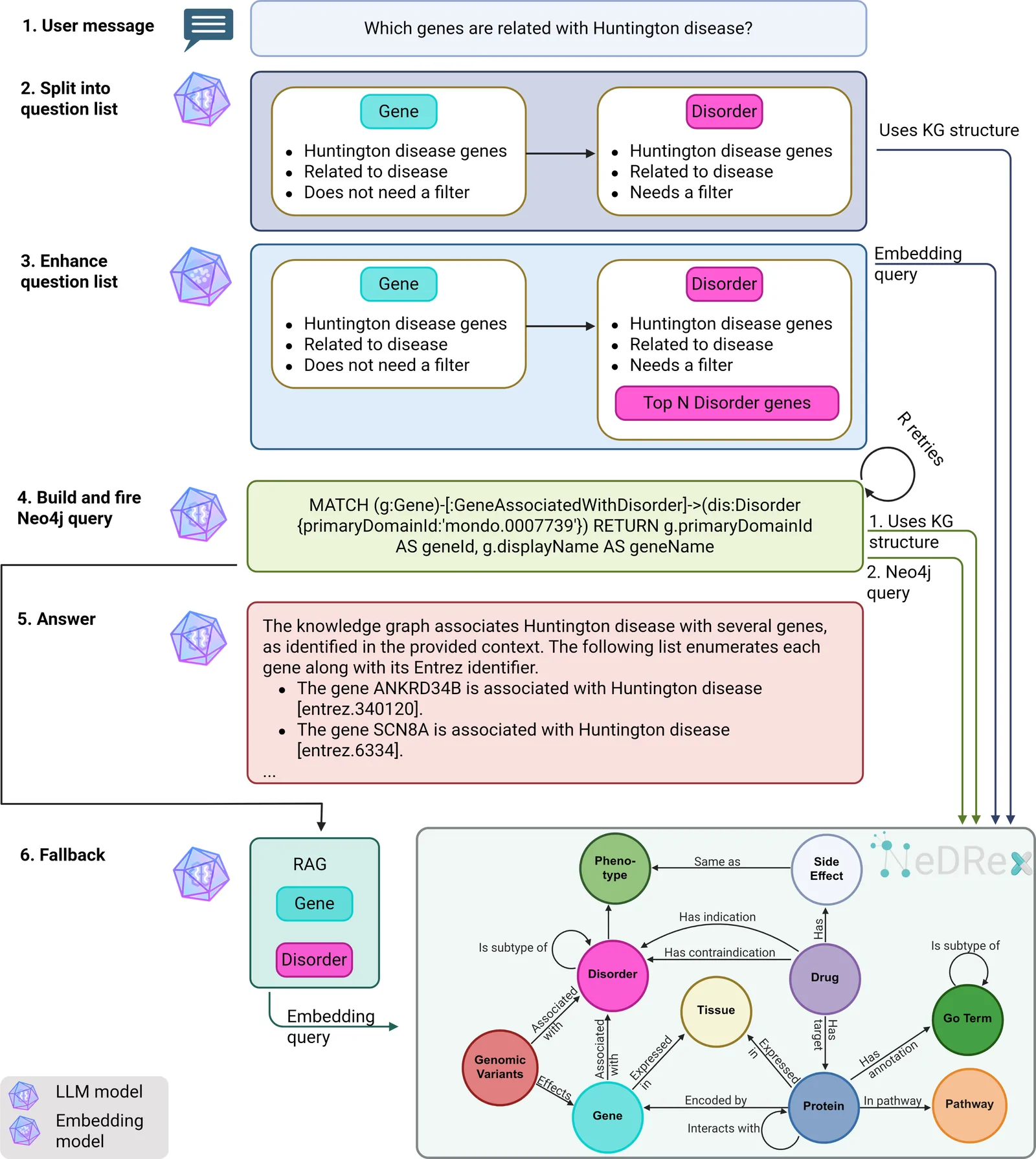
Knowledge graph (KG) agent architecture. This workflow illustrates the generic processing steps of this agent. A user prompt (1) is decomposed into a structured list of subqueries (eg, gene and disorder; 2). Then, these subqueries are expanded using embeddings and the NeDRex (Network-based Drug Repurposing and Exploration) KG schema (3). Subsequently, a Cypher query is formulated and executed on the NeDRex Neo4j database (4). The results are returned as a response (5). In the event of query failure, a fallback step to Retrieval-Augmented Generation (RAG) is initiated (6). Figure created in bioRender [32]. LLM: large language model.

### Evaluation

Evaluating LLM-based agent systems is challenging because, unlike deterministic programs, their outputs are inherently interpretive and nondeterministic, meaning there is often no single objective ground truth beyond simple accuracy metrics. Unlike deterministic systems, LLMs generate responses that encompass generation processes, complex interpretation, and decision-making mechanisms [53]. Additionally, stochastic LLM outputs can lead to variability across runs. The aforementioned problems result in a multifaceted evaluation challenge [54]. First, it must be verified whether the model uses external tools correctly and appropriately. The next step is to check that the resulting information has been processed and communicated correctly. Furthermore, LLMs have the capacity to generate statements that, while superficially plausible, are factually inaccurate, thereby introducing a degree of complexity to the evaluation process [55]. Quantitative assessment is only feasible for well-defined subtasks with an explicit ground truth (eg, exact database queries). Conversely, higher-level outputs, such as literature support, scientific interpretation, and argument completeness, lack a unique ground truth and are therefore only partially and qualitatively assessable [55]. A robust evaluation, therefore, requires a combined approach of automated metrics and manual validation [53]. To evaluate the accuracy of the answers created by an LLM, an LLM-as-a-judge model is used. An LLM-as-a-judge model evaluates the quality, accuracy, or relevance of answers using an LLM to simulate human judgment in tasks such as evaluation, ranking, or criticism [56]. All LLM-as-a-judge evaluations were performed by a single fixed judge model, gpt-oss:20b, regardless of which model generated the answers under assessment. The judge was run with a temperature of 1 and top-P of 0.9 (Multimedia Appendix 3). Given the potential for such distortions to be influenced by hallucinations, the process was closely monitored by experts and underwent corrective adjustments as needed. All evaluations were run thrice with gpt-oss:20b, nemotron-3-nano:4b, qwen3.6:27b, and gemma4:e4b. All model parameters can be found in Multimedia Appendix 3. Individual reported numbers represent the average of the best-performing model.

### Tool Validation

*Tool accuracy* is defined as the ability of an agent to identify and select the correct tool in response to a user’s input. For each tool, 100 evaluation questions were generated, resulting in a total of 400 queries. To assess tool selection under different prompting styles, 50 questions per tool explicitly contained tool-related keywords, whereas the remaining 50 questions described the desired task in natural language without mentioning the tool itself. This setup allowed us to evaluate both keyword-based and semantic tool selection capabilities. To further assess robustness to imperfect user input, 10 questions within each tool category were modified to include realistic human typing errors, such as misspellings, omitted characters, and typographical mistakes. The evaluation questions were generated synthetically to reduce manual workload, ensure a consistent structure, and maintain linguistic variation. A ground-truth dataset was constructed manually by selecting NeDRex diseases with associated genes available as gene symbols, UniProt identifiers, and Entrez Gene identifiers. These identifiers were incorporated into the questions and a dedicated extraction field, and the corresponding correct translations were recorded as ground truth. During testing, only the translation endpoint of the NeDRex API was executed live. The evaluation of all NeDRex tools and the network agent was conducted using a precomputed set of results associated with each question in the test set. This approach was implemented to optimize the evaluation runtime. The evaluation followed a 2-stage procedure. *Call accuracy* measures correct tool and API invocation, while *answer accuracy* evaluates the correct reflection of returned results in generated answers. This setup isolates tool-selection errors from errors in result interpretation and enables a focused assessment of ChatDRex’s tool accuracy.

### KG Agent

To evaluate the NeDRex KG tool, we created 174 questions to determine whether the result set returned by the agent represents a superset of the silver standard annotation dataset (SSAD) created by human experts. The SSAD for the NeDRex KG evaluation was created using a self-generating and self-evaluating mechanism that leverages several language models and targeted validation steps (Section S8 in Multimedia Appendix 1). We manually reviewed the generated question-Cypher pairs, rephrasing queries where necessary to ensure they were biologically meaningful and logically sound. The end result was a manually corrected dataset. For evaluation purposes, each returned element must contain all properties defined in the SSAD, such as the biological meaning of the natural language question and the semantic relatedness between the natural language question and the corresponding Cypher query. In the comparison between the ground truth and generated answer, additional attributes were allowed provided that no expected elements were missing and no additional semantically inappropriate entries were introduced. A matching procedure was implemented to calculate the *F*_₁_-score. This procedure identifies a possible hit in the LLM result for each manually curated question. Such a hit is only counted if all values of the SSAD result are contained in the corresponding LLM entry. We compared the results with GraphRAG and a classic CypherQA chain [57]. To contextualize the performance of the NeDRex KG tool, we compared its final answer outputs with those produced by a GraphRAG baseline and a conventional CypherQA chain [47,58]. For these baseline comparisons, we evaluated only the final returned results and did not assess intermediate reasoning steps, tool calls, or query-generation trajectories.

### Research Agent

To quantitatively evaluate the research agent, we developed a benchmark for answering biomedical literature questions based on the publicly available PubMedQA dataset [59,60]. We assessed answer correctness with an LLM-as-a-judge procedure, using the PubMedQA question, the research agent’s response, and the reference answer as context. Responses were marked correct if they were semantically consistent with the reference answer. This included cases with additional relevant, literature-based context that did not contradict the expected conclusion. This semantic evaluation was appropriate as the agent produces free-text evidence syntheses rather than categorical yes/no/maybe labels.

### Technologies Used

ChatDRex is built on LangChain4j and Quarkus Support to streamline complex, network-based drug repurposing analyses through a conversational interface [61-63]. The frontend (Angular) connects to Quarkus servers, which communicate via WebSocket [64]. In addition to the WebSocket server, the backend offers a Representational State Transfer (REST) API and a model context protocol (MCP) server [65]. These provide the most important tools for external integration. These features make the frontend interchangeable and integrable into other LLM workflows [65]. ChatDRex utilizes, for example, gpt-oss:20b [66], a highly capable LLM developed by OpenAI and optimized for helpful responses (Multimedia Appendix 3). It is hosted through a self-hosted Ollama instance [67]. We use Ollama behind a self-hosted OpenWebUI that allows access control [68]. Furthermore, ChatDRex’s multi-agent architecture is designed to be flexible, allowing the integration of new and improved LLMs at the individual agent level as they become available. This modularity ensures that developers can add new algorithms, external APIs, or domain-specific applications through dedicated tool wrappers and service connectors without altering the core dialogue and orchestration logic. Consequently, ChatDRex remains adaptable to advances in LLMs and biomedical software. Presently, this extensibility is developer-mediated rather than fully self-service; arbitrary user-provided tools are not yet dynamically plugged in at runtime without a wrapper implementation.

### Ethical Considerations

This was a small-scale user study in which participants tested the ChatDRex application. In accordance with the screening procedure of the Ethics Commission of the Department of Informatics, University of Hamburg, the completed basic questionnaire (“Basisfragebogen”) indicated that the study posed no more than minimal risk to participants and involved no deception, no vulnerable populations, and no processing of sensitive personal data. Formal review by the Ethics Commission was therefore not required. The study was conducted in accordance with the Declaration of Helsinki and the data protection requirements of the University of Hamburg. All participants provided informed consent prior to participation.

## Results

### Tool Validation

ChatDRex provides end-to-end conversational analysis for network-based drug repurposing. It accomplishes this by following a workflow proposed, amongst others, in NeDRex [7], but within a chatbot-like environment. ChatDRex combines schema-constrained KG querying, network module expansion (DIAMOnD), drug prioritization (TrustRank or closeness centrality), and in silico validation based on functional coherence (DIGEST). In addition, literature research is integrated into the workflow. These findings are presented as interactive networks in-chat with a consistent visual legend across analyses using Drugst.One for visualization [6]. Examples can be found in Multimedia Appendix 4.

Beyond qualitative use cases, we quantitatively evaluated the agent tool interactions underlying ChatDRex (Table 1). Network-based tools, such as DIAMOnD, TrustRank, and closeness centrality, achieved near-perfect tool accuracy ranging from 0.97 (SD 0.00) to 0.99 (SD 0.00), indicating robust recognition of intention and correct tool selection by the agent. In contrast, the DIGEST-based analysis showed slightly lower tool and call accuracies (mean 0.85, SD 0.01 and mean 0.77, SD 0.01, respectively), reflecting the increased complexity of mapping closely related analytical tasks from user prompts.

**Table 1.** Overview of the evaluated agents and their tools.

Agent, model, and tool			Tool accuracy, mean (SD)	Call accuracy, mean (SD)	Answer accuracy, mean (SD)
NeDRex^a^					
qwen3.6:27b					
Closeness centrality			0.98 (0.01)	0.97 (0.01)	—^b^
TrustRank			0.97 (0.00)	0.96 (0.01)	—
gpt-oss:20b					
DIAMOnD^c^			0.99 (0.00)	0.96 (0.02)	—
DIGEST^d^					
gpt-oss:20b					
DIGEST Set			0.85 (0.01)	0.77 (0.01)	0.3 (0.01)^e^; 0.66 (0.01)^f^
Knowledge graph					
qwen3.6:27b					
NeDRex knowledge graph^g^			—	0.82 (0.01)	0.76 (0.03)
Research					
qwen3.6:27b					
Semantic Scholar			—	—	0.93 (0.01)

a NeDRex: Network-based Drug Repurposing and Exploration.

b Not applicable.

c DIAMOnD: Disease Module Detection.

d DIGEST: In Silico Validation of Disease and Gene Sets, Clusterings or Subnetworks.

e Raw answer score obtained using the automated LLM-as-a-judge evaluation.

f Corrected answer score obtained after manual assessment by human experts.

g The NeDRex knowledge graph tool is evaluated using *F*₁-scores.

For network expansion and prioritization tools (DIAMOnD, TrustRank, and closeness centrality), the final outcome presented to the user is determined directly by the underlying API outputs (Figure 5A, B, and D). The LLM does not perform additional interpretation in these cases but returns the deterministic results produced by NeDRex, such that tool and call accuracies fully characterize the system performance. In contrast, DIGEST-based functional enrichment requires the LLM to interpret structured enrichment results and summarize them in natural language, introducing an additional reasoning layer that was explicitly evaluated (Figure 5F). Accordingly, DIGEST showed reduced answer accuracy and was strongly penalized by the automated LLM-as-a-judge, which achieved only 0.3 (SD 0.01) accuracy for this task, despite the underlying analytical information being factually correct. Manual expert evaluation of the same DIGEST outputs yielded a substantially higher answer accuracy of 0.66 (SD 0.01), highlighting a pronounced mismatch between automatic LLM-based judging and human assessment. This discrepancy raised the question of whether LLM-based judging can be trusted more broadly. To address this, 20 randomly sampled answers from each evaluation area were independently assessed by human raters. Across all other components, human and automated judgments showed no significant differences, confirming the reliability of the LLM-as-a-judge in general. The mismatch is therefore specific to DIGEST. Here, outputs require interpreting complex enrichment results, and these results are expressed in natural language. This task is not well suited for current automatic evaluation schemes.

**Figure 5.**
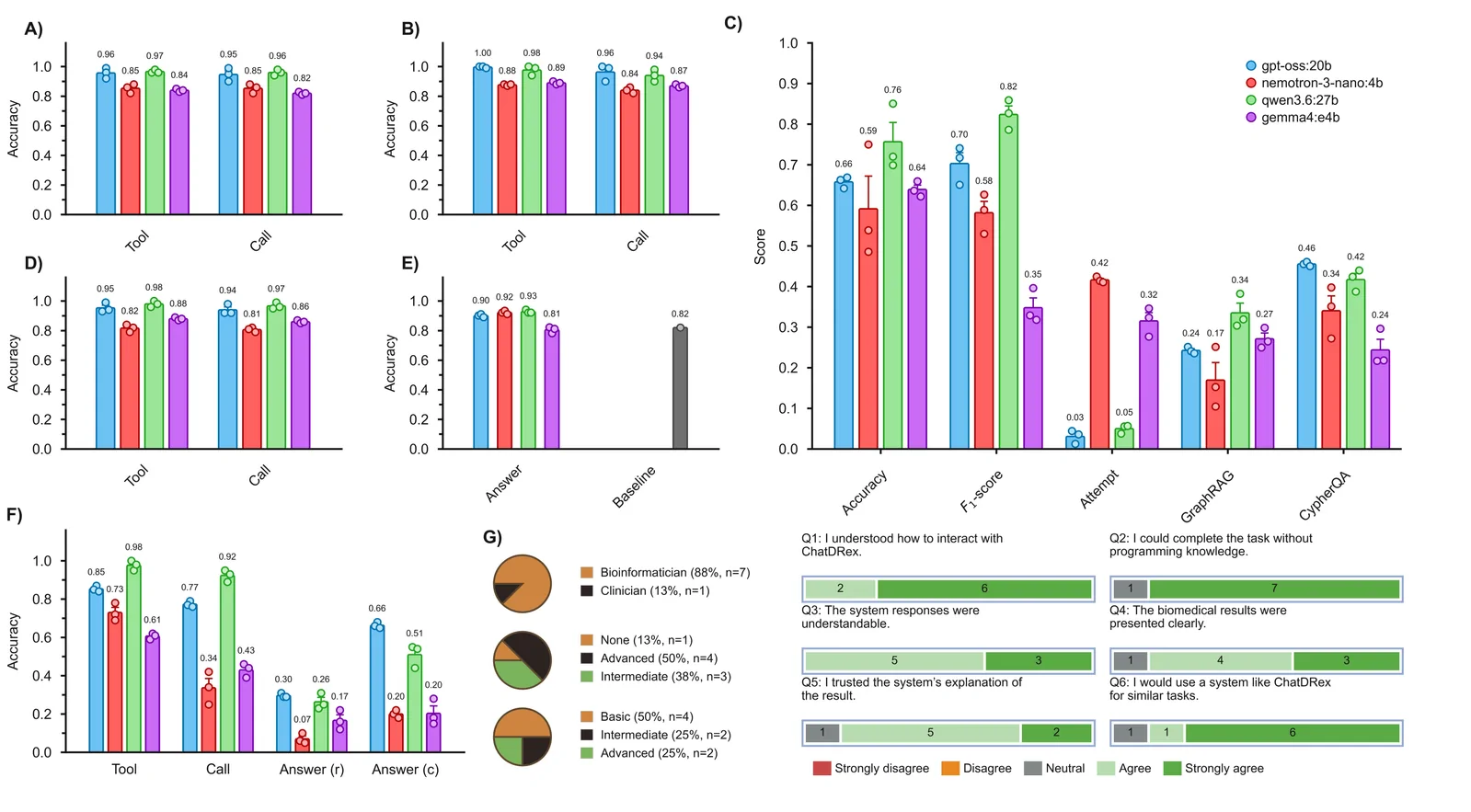
Quantitative performance and usability evaluation of ChatDRex. The tool-specific benchmark results for the ChatDRex agent are shown across the core functional modules: (A) TrustRank, (B) DIAMOnD (Disease Module Detection), (C) NeDRex (Network-based Drug Repurposing and Exploration) knowledge graph (KG) querying, (D) Closeness centrality, (E) Literature research (baseline is GPT-4, Medprompt) [60,69], and (F) DIGEST (In Silico Validation of Disease and Gene Sets, Clusterings or Subnetworks) enrichment analysis (answer (r) denotes the raw answer before correction, and answer (c) denotes the corrected answer after manual assessment by human experts). Bars indicate aggregate performance metrics for tool accuracy, call accuracy, and answer accuracy, where applicable, across the evaluated models. For the NeDRex KG benchmark (C), model-wise performance is shown for multiple evaluation criteria, including answer accuracy, *F*_1_-score, needed attempts (normalized from 1 to 3 attempts in the range of 0 to 1), graph query handling, and Cypher query generation. Individual points indicate replicate or question-level performance values, and bars summarize the corresponding mean scores. (G) Usability assessment of ChatDRex in a pilot user study. Participant background is summarized by professional role, programming experience, and experience with bioinformatics or network biology. Percentages shown for participant characteristics are rounded to the nearest whole number. The questionnaire assessed whether users understood how to interact with ChatDRex, could complete tasks without programming knowledge, perceived system responses as understandable, considered biomedical results to be clearly presented, trusted system explanations of results, and would use a system like ChatDRex for similar tasks. Responses are shown on a 5-point Likert scale ranging from strong disagreement to strong agreement. Overall, the results demonstrate that ChatDRex supports the accurate orchestration of network medicine tools and biomedical information retrieval while remaining accessible to users with diverse computational and biomedical backgrounds, and it was rated as understandable and usable by the pilot cohort, which consisted predominantly of computationally experienced users.

To further investigate this discrepancy, all DIGEST responses were manually re-evaluated by a human annotator using the NeDRex API as an external reference for verifying the correctness of the generated answers. This reassessment confirmed that several responses originally marked as incorrect by the automatic evaluator were in fact correct, indicating that the observed disagreement was primarily caused by limitations of the evaluation procedure rather than by errors in the generated analyses. Consequently, manual verification supported by domain-specific resources remains necessary for reliably assessing such outputs. These findings highlight the need for more specialized evaluation frameworks for biomedical LLM systems. Generic answer-similarity metrics or broad LLM-as-a-judge scores may insufficiently capture whether an answer is biologically plausible, mechanistically grounded, supported by retrieved evidence, and safe for downstream biomedical interpretation. Future work should therefore prioritize the development of domain-specific evaluation metrics that jointly assess factual correctness; evidence grounding; biomedical reasoning quality; uncertainty handling; and the distinction among partially correct, unsupported, and biologically misleading outputs.

Importantly, this evaluation artifact does not reflect a flaw in ChatDRex’s analytical pipeline. The consistently high tool and call accuracies across all components demonstrate that the underlying results are methodologically sound and can be trusted. Taken together, these findings show that ChatDRex provides reliable and reproducible tool outputs, while improving the automatic evaluation of complex natural language summaries remains the primary bottleneck for further development.

### KG Agent

The evaluation of schema-constrained NeDRex KG queries showed that the proposed agent improves automatic Cypher query generation for accessing biomedical KGs while also highlighting remaining limitations in translating complex natural language queries into complete Neo4j statements. The strongest performance was observed for qwen3.6:27b, which achieved the best overall results with an average accuracy of 0.76 (SD 0.03) and a call accuracy (*F*_₁_-score) of 0.82 (SD 0.01; Figure 5C). For this model, the fallback was used once, and in 5% of cases, more than one run was required to generate a correct Cypher query. The gpt-oss:20b model demonstrated the second-best performance, achieving an average accuracy of 0.66 (SD 0.01) and a call accuracy (*F*_₁_-score) of 0.70 (SD 0.02). In contrast, smaller models demonstrated lower and less stable performance, with nemotron-3-nano:4b achieving an average accuracy of 0.59 (SD 0.06) and an *F*_₁_-score of 0.58 (SD 0.02) and gemma4:e4b achieving an accuracy of 0.64 (SD 0.01) and an *F*_₁_-score of 0.35 (SD 0.02).

The CypherQA baseline achieved values of 0.46 (SD 0.00) for gpt-oss:20b, 0.34 (SD 0.03) for nemotron-3-nano:4b, 0.42 (SD 0.01) for qwen3.6:27b, and 0.24 (SD 0.02) for gemma4:e4b. On the other hand, GraphRAG’s performance was lower on structure-specific KG questions, achieving values of 0.24 (SD 0.00), 0.17 (SD 0.04), 0.34 (SD 0.02), and 0.27 (SD 0.01), respectively. Overall, these results demonstrate that the KG agent benefits from the proposed architecture.

### Research Agent

For the evaluated PubMedQA questions, all tested local models achieved high semantic answer accuracy. The best-performing configuration was qwen3.6:27b, with an answer accuracy of 0.93 (SD 0.01), closely followed by nemotron-3-nano:4b (accuracy of 0.92, SD 0.01) and gpt-oss:20b (accuracy of 0.90, SD 0.00). The gemma4:e4b model achieved a lower but still solid value of approximately 0.81 (SD 0.01; Figure 5E). As expected, a larger model was associated with better results in most cases. For comparison, the GPT-4 (Medprompt) baseline achieved a value of 0.82 [60,69]. Thus, the best ChatDRex research agent configurations in this evaluation outperformed the GPT-4 (Medprompt) baseline and performed significantly better than the originally reported accuracy of the fine-tuned BioBERT PubMedQA [69].

Importantly, the research agent was not limited to the single summary associated with each PubMedQA entry. Instead, it accessed an average of more than 30 literature sources per question, allowing the system to synthesize insights from multiple publications. This explains why the evaluation should not be interpreted as a standard PubMedQA ranking comparison but rather as an end-to-end benchmark for the retrieval and synthesis of biomedical literature using PubMedQA questions as controlled reference tasks [59]. The results show that ChatDRex can leverage external literature sources to generate semantically correct biomedical answers and that its literature-enhanced research workflow offers a clear advantage over purely fine-tuned, closed-context models for this type of biomedical QA. Drugst.One was excluded from the quantitative agent evaluation (see the *Limitations* section).

### Hallucination Validation

To evaluate how hallucinations are handled, we created a set of 10 curated biomedical multistep reasoning cases. These cases were designed to test the detection and correction of hallucinations under controlled conditions (Figure 6). Each case included an unsupported conclusion. For instance, therapeutic, causal, drug-target, drug-disease, pathway, or variant-disease claims that were not directly entailed by the retrieved evidence could be introduced.

We evaluated ChatDRex to determine if its grounding and validation mechanisms could detect unsupported answers, trigger corrections, and produce final answers consistent with NeDRex and the literature. The detection and correction score reflects how often unsupported drafts were successfully identified and corrected. The hallucinated draft score quantifies the grounding risk of the initial unsupported answer, while the corrected answer score measures the remaining unsupported content after correction. Across the evaluated models and repeated runs, ChatDRex achieved high detection and correction scores, with complete correction in most runs (Figure 6). The mean hallucinated draft score remained high across models, whereas the corrected answer score decreased substantially, indicating improved grounding after correction. A representative case demonstrated that an unsupported claim that imatinib targets *EGFR* was rejected and revised to reflect that imatinib is only supported for targeting *ABL1*, *KIT*, and *PDGFRA*. Thus, *EGFR*-based repurposing is not supported by the retrieved evidence.

**Figure 6.**
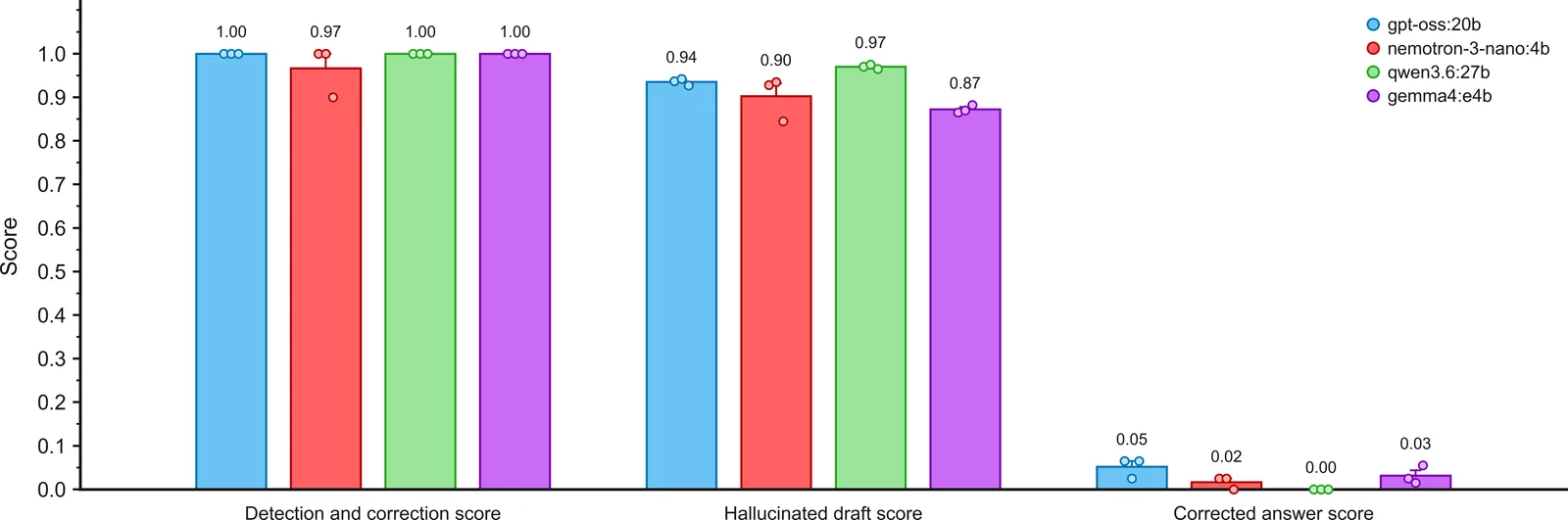
Evaluation of hallucination detection and correction in ChatDRex*.* The performance of the grounding guardrail was evaluated using 10 curated, multistep biomedical reasoning cases that contained unsupported draft conclusions. For each case, the finalization model first produced an incorrect draft and then a corrected draft after guardrail-triggered reprompting. Bars indicate the mean performance of each model across repeated runs, and points indicate individual runs. The detection and correction score represents the fraction of cases in which the unsupported draft was detected and successfully corrected. The hallucinated draft score quantifies the grounding-risk score assigned to the initial unsupported answer. The corrected answer score quantifies the residual unsupported content after correction. Across models, hallucinated drafts received high scores, while corrected answers received markedly lower scores. This indicates that the grounding and validation mechanisms successfully suppress unsupported biomedical claims before generating the final answer.

### User Study

To evaluate the usability of ChatDRex, we conducted a pilot study aimed at obtaining a user-centered assessment of ChatDRex’s usability (Figure 5G). The study participants included 7 bioinformaticians and 1 clinician (3 male and 5 female experts). To assess the predictions and their comparability, these participants were asked to review and evaluate the examples (Multimedia Appendix 4). The questionnaire can be found in Multimedia Appendix 5. In the questionnaire, participants reported good comprehensibility and user friendliness. Participants expressed confidence in the accuracy of ChatDRex’s predictions, as sources and specific links between KG nodes were included.

### Example Workflow for Huntington Disease

Through all the previously presented steps, ChatDRex provides researchers with an interface to access and analyze biomedical data while maintaining conversation coherence and system reliability. The system implicitly translates between gene and protein identifiers and efficiently handles secure connections to the NeDRex database through a custom API client (Tables S1 and S3 in Multimedia Appendix 4), facilitating the comprehensive exploration of genomic data relationships. To demonstrate the platform’s capabilities, we present concrete use cases that align with common workflows in drug repurposing research (Figure 7; Table S5 in Multimedia Appendix 4).

The platform interacts with NeDRex, which enables schema-constrained Cypher queries to retrieve disease indications and related associations directly from the KG. It also uses DIAMOnD for disease module detection and DIGEST to estimate these modules’ functional coherence based on enriched pathways and functional annotations (Tables S1 and S4 in Multimedia Appendix 4). TrustRank is applied to prioritize proteins and drugs for assessing network reliability (Table S5 in Multimedia Appendix 4). Finally, ChatDRex uses the research agent to facilitate targeted literature searches and summarize relevant findings about drugs and diseases (Table S2 in Multimedia Appendix 4). Multimedia Appendix 4 provides step-by-step workflows, intermediate outputs, and user interface snapshots for each use case.

**Figure 7.**
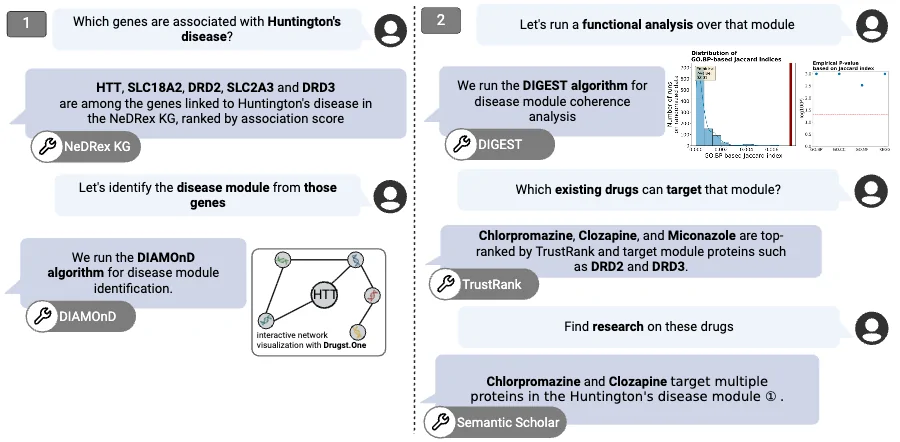
A manually condensed example workflow for Huntington disease. A user initiates a query about Huntington disease. ChatDRex identifies relevant genes, constructs a disease module using DIAMOnD (Disease Module Detection), visualizes it with Drugst.One, performs a function-based module coherence analysis via DIGEST (In Silico Validation of Disease and Gene Sets, Clusterings or Subnetworks), prioritizes candidate drugs with TrustRank, and retrieves supporting literature through Semantic Scholar, ultimately providing a comprehensive, conversational analysis of potential drug repurposing opportunities. Drug prioritization by TrustRank reflects network-based interactions between drugs and proteins of the disease module and does not imply clinical efficacy or validation for Huntington disease. The Semantic Scholar step shows that ChatDRex retains outputs from earlier steps. However, the literature search is based only on the prioritized drugs and is not limited to Huntington disease; therefore, the retrieved publications may concern other conditions. This example can be replicated with the help of Table S5 in Multimedia Appendix 4, where we provide a detailed workflow. Figure created in bioRender [32].

### Example Workflow for the Colorectal Cancer Module

ChatDRex has the ability to identify disease-related gene modules in biological networks using its integrated tools. Starting with the disease term *colorectal cancer*, ChatDRex retrieved a curated seed gene set of 38 canonical colorectal cancer genes from NeDRex, including genes involved in Wnt signaling, such as *APC*, and in DNA repair and damage response, such as *MLH1*, *MSH2*, and *TP53*. These genes served as disease-associated seeds for network expansion.

The DIAMOnD algorithm expanded the seed set by prioritizing genes with significant connectivity to the seed genes within the NeDRex protein-protein interaction network, yielding a disease module of 138 nodes and 2129 edges, which comprised the 38 seed genes and 100 genes added by DIAMOnD. Functional interpretation of the top 10 genes was executed via the ChatDRex DIGEST agent, which evaluated functional coherence by comparing observed annotation consistency against randomized gene sets. The returned DIGEST coherence scores indicated significant signals for cellular compartments (cytoplasm, nucleus, nucleoplasm, cytosol, and chromatin) and for cancer-related signaling pathways (pathways in cancer, Ras signaling, and PI3K-Akt signaling). The empirical *P* values were .000999 for both the Gene Ontology (GO) Cellular Component and Kyoto Encyclopedia of Genes and Genomes (KEGG) pathway annotation layers and .002 for GO Biological Process, which had been derived from permutation-based reference distributions. Given the broad nature of the most frequent cellular component terms and the exploratory nature of permutation-based coherence testing, these results can be interpreted as indicative rather than confirmatory, but they are consistent with biological processes previously implicated in colorectal cancer.

The top 10 genes (*AKT1*, *SMARCA4*, *FBXW7*, *SMAD1*, *FANCC*, *PTPN11*, *FGF23*, *SMAD4*, *FGF6*, and *PTEN*) emerged as highly connected genes within the expanded module, collectively spanning PI3K-Akt signaling, growth factor signaling, TGF-β/SMAD signaling, chromatin remodeling, and DNA repair. These genes represent candidates for further investigation, as their network positions suggest they may integrate multiple layers of colorectal cancer pathogenesis. However, direct interactions among them could only be partially verified in the NeDRex KG and the literature.

## Discussion

### Principal Findings

ChatDRex demonstrates that a conversational, multi-agent architecture can reliably orchestrate established network medicine tools for drug repurposing without requiring users to interact with the underlying pipelines directly. Across all evaluated components, network expansion, drug prioritization, and KG querying, ChatDRex achieved consistently high tool and call accuracies, indicating that the system correctly identifies and invokes the appropriate backend methods in response to user prompts. The resulting outputs are largely determined by validated, deterministic algorithms rather than by unconstrained model generation, which accounts for the stability and reproducibility observed across repeated runs.

Reliability, however, differs across pipeline stages. For network expansion and prioritization, performance reflects correct orchestration of deterministic backend methods, and the outcome is directly determined by the returned tool outputs. For DIGEST-based functional analysis, we observed comparatively weaker performance. This task requires the model to abstract structured coherence and enrichment statistics into a natural language summary, a genuine generative step that the other tools do not involve. This could impact the results.

### Comparison With Prior Work

ChatDRex sits at the intersection of 2 lines of work that have so far developed independently. Network medicine platforms, such as NeDRex, provide validated, deterministic methods for disease module identification and drug prioritization but presuppose that the user can appropriately use the interface, assemble a parameterized pipeline, and interpret raw network output. Their expressive power is therefore accessible primarily to bioinformaticians rather than clinicians and experimental biologists who generate the underlying hypotheses.

Conversely, general-purpose LLMs and biomedical LLM assistants offer a natural language interface but lack grounding in curated biomedical KGs. Without schema-constrained query generation and validated analytical backends, such systems are prone to unverifiable output and cannot guarantee that a returned drug candidate derives from an established prioritization method. Recent agentic systems in biomedicine have begun to couple LLMs with external tools but typically evaluate end-to-end answer quality without isolating tool selection from result interpretation.

ChatDRex differs in coupling both components explicitly. User prompts are translated into schema-constrained Cypher queries to retrieve relevant information from the NeDRex KG, while downstream analyses are performed by established tools, such as DIAMOnD, TrustRank, closeness centrality, and DIGEST, rather than by the model.

### Limitations

There are some limitations. First, a key limitation is that our evaluation establishes only that ChatDRex reliably selects and invokes the underlying methods and faithfully reports their outputs—not that the resulting candidates are clinically valid. We performed no retrospective validation against known drug-disease indications and tested no candidates experimentally. ChatDRex’s predictive validity for actual clinical drug repurposing therefore remains unverified, and its outputs should be regarded as hypothesis-generating rather than clinically actionable.

Second, the NeDRex KG benchmark may be affected by generation bias within the model family. The SSAD used to evaluate natural language-to-Cypher query generation was created with the help of several language models, including qwen2.5-coder:7b. It was then manually reviewed and corrected. Since qwen3.6:27b was the top performer in the NeDRex KG evaluation, its strong performance may be partly due to its alignment with query structures, wording patterns, or Cypher syntax produced by a related model family. This potential confounding factor should be considered when interpreting the relative model ranking.

Third, Drugst.One was not tested as part of the quantitative agent evaluation because it primarily provides a visual representation layer without adding contextual or analytical functions directly relevant to the agent’s reasoning and decision-making capabilities.

Fourth, all automated assessments of response quality were performed by a single judge model (gpt-oss:20b), which was also among the evaluated systems, and thus, self-preference bias cannot be excluded for that configuration. Independent models could provide a more reliable assessment. We took this into account by manually reviewing the responses and correcting them where necessary.

Fifth, the pilot cohort consisted of 7 bioinformaticians and a single clinician, most with intermediate to advanced programming experience. The results reflect usability for computationally experienced users and do not yet validate no-code usability for novice or noncomputational users.

### Future Directions

Several directions follow directly from the limitations above. First, prospective validation is required: candidate drugs prioritized by ChatDRex for a given disease module should be assessed against experimental readouts or, where available, retrospective clinical evidence, in order to establish predictive rather than procedural validity.

Second, the contribution of the visualization layer warrants quantitative assessment. Although Drugst.One does not participate in the agent’s reasoning, its effect on user comprehension and hypothesis formation can be measured through an extended usability study.

Finally, the step where the LLM turns DIGEST’s statistical results into an explanation offers the clearest room for improvement, as this is where the most errors appear. Approaches that better structure or constrain this interpretation step, potentially through task-specific fine-tuning, could reduce these errors while preserving the reliable and reproducible results delivered by the deterministic DIGEST tool.

### Conclusions

By automating query formulation, network construction, prioritization, visualization, and literature grounding, ChatDRex can shoulder a substantial portion of analytical work, allowing scientists to focus on interpretation, hypothesis refinement, and experimental design. It thereby lowers the technical barrier to network-based drug repurposing without displacing the validated methods on which such analyses depend.

## Supplementary material

10.2196/93862 Multimedia Appendix 1 Detailed descriptions of the ChatDRex agents (summary, planning, DIGEST, NeDRex, knowledge graph, research, and finalize) and the underlying algorithms DIAMOnD, TrustRank, and closeness centrality, including the silver standard annotation dataset generation procedure for the NeDRex knowledge graph evaluation.

10.2196/93862 Multimedia Appendix 2 Guardrail implementation and evaluation: prompt injection filtering, pre-execution workflow plausibility check, and results of the 100 curated safeguard test cases.

10.2196/93862 Multimedia Appendix 3 Sampling and context parameters of all language models used in this study.

10.2196/93862 Multimedia Appendix 4 Use cases: step-by-step ChatDRex workflows, agent planning decisions, tool calls, intermediate outputs, and user interface snapshots for functional enrichment analysis, literature search, network analysis, disease module detection, and the Huntington disease module detection and drug prioritization workflow.

10.2196/93862 Multimedia Appendix 5 ChatDRex usability feedback form and overview of participant responses collected during the usability pilot study.

## Abbreviations

CoT: chain-of-thought
DIAMOnD: Disease Module Detection
DIGEST: In Silico Validation of Disease and Gene Sets, Clusterings or Subnetworks
GO: Gene Ontology
ICL: In-Context Learning
KEGG: Kyoto Encyclopedia of Genes and Genomes
KG: knowledge graph
LLM: large language model
MCP: model context protocol
NeDRex: Network-based Drug Repurposing and Exploration
QA: question answering
RAG: Retrieval-Augmented Generation
REST: Representational State Transfer
SSAD: silver standard annotation dataset
