# Supplementary material for: Conversational Multi-Agentic Network-Driven Disease Module Identification and Drug Repurposing Prediction With ChatDRex: Platform Development Study
Source: JMIR AI. 2026 Nov 3;5:e93862. doi: 10.2196/93862 (PMC13730514; doi:10.2196/93862)
Supplement: Supplementary file 1 [file ai-v5-e93862-s001.pdf]

Multimedia Appendix 1. ChatDRex Agents

Section S1: Summary Agent

The **Summary agent's** function is to summarize previous steps based on the current input, with the objective of saving tokens and improving the ability to respond to previous questions and answers [1]. This allows the reset of all agents' memory to zero and the reinitialization of the summary. The agent uses an input guardrail to check for prompt injection and returns an error message if it is present.

Section S2: Planning Agent

The **Planning agent** functions as an orchestration agent to our multi-agent system, forwarding human input to the appropriate agent depending on the agent type [2]. The system utilizes an LLM to analyze requests and determine the appropriate agent to which they should be directed. The solution to this problem is achieved through the completion of N steps (in our case N=6). To identify the most suitable agent, the LLM is provided with the number of remaining steps and the current state each time it is invoked. The state contains the responses from the previously contacted agents. This approach enhances efficiency and accuracy by ensuring that each request is handled by the most suitable specialized agent, thereby optimizing system performance through task specialization [3].

Section S3: DIGEST Agent

The **DIGEST agent** provides *in silico* validation for generated disease modules to assess their relevancy [4]. DIGEST follows the hypothesis that disease modules, or clusters therein, should capture specific molecular machinery that all together should be associated with the same functions. A logical consequence is that functional enrichment analysis results for clusters of genes, such as the ones generated by disease module identification methods, should be coherent, meaning that all captured proteins or genes can actually be found in the same functional context according to GO and KEGG annotation. As disease module search usually starts with a list of seed genes that are supposed to define the disease context, DIGEST offers the use of those seeds as the reference the module set of genes is compared to, estimating if the functional context and coherence remain the same between seed genes and disease module. To make results interpretable DIGEST distills the functional coherence scores into an empirical p-value by randomly creating seed sets using selectable random background models and comparing the functional coherence score of these random sets to the observed one. Specifically for network-based methods, DIGEST provides a subnetwork mode, allowing the network-based sampling of random modules.

In summary, DIGEST facilitates *in silico* disease module validation through functional coherence analysis, here used to evaluate disease modules. In contrast to conventional enrichment approaches, DIGEST quantifies the coherence of functional annotations within a gene set by comparison to comparable random gene sets, yielding a p-value that reflects the statistical significance of functional coherence rather than merely listing enriched terms or an uninterpretable functional coherence score. Through the use of LLMs, the agent interfaces with DIGEST via API calls to assess the relevance and statistical significance of disease modules and disease-associated genes. To ensure biological validity, the agent applies a predefined decision logic when invoking DIGEST. As subnetwork-based functional coherence analysis is only meaningful for gene sets derived from prior network-based disease module inference (e.g., DIAMOnD or TrustRank), the agent first checks its internal memory for an existing network context. If such context is available, DIGEST is executed on the derived subnetwork; otherwise, it is applied directly to the user-provided gene set. The LLM-based agent then presents both graphical and textual results through its chatbot-like

interface, facilitating accessible interpretation of functional coherence and pathway enrichment results.

Section S4: NeDRex Agent

The **NeDRex agent** is responsible for queries pertaining to the analysis of genes and their interactions from the NeDRex knowledge graph [5]. In the system, proteins and genes are internally translated between, but for simplicity within the chatbot and this study, they will be used interchangeably, as is common within the reductionistic scope of network medicine. In this framework, biological entities, such as genes, proteins, drugs, and diseases, are represented as nodes [5]. Molecular or biomedical relationships, such as protein-protein interactions, drug-target associations, or disease-gene associations, are represented as edges. Additionally, the NeDRex API allows network expansion via several tools, enabling ChatDRex the use of DIAMOnD for disease module identification and TrustRank and closeness centrality for drug ranking [5]. Network-based drug repurposing is based on the assumption that complex diseases are often reflected by perturbations of interacting molecular systems rather than isolated genes or proteins. This, in combination with the understanding that population-level information can be both noisy and incomplete at the same time, requires the use of specific algorithms, designed to identify key additions to a disease module given an initial list of seed genes, that should reflect the disease context. The NeDRex platform was developed for specifically network-based drug repurposing research. It integrates heterogeneous biomedical data to support disease module discovery, drug prioritization, and statistical validation [5].

Section S4.1: NeDRex Agent Tool DIAMOnD

DIAMOnD is an algorithm designed to detect disease modules by identifying additional disease-relevant proteins around a set of known disease-associated seed proteins [6]. The algorithm is based on the observation that proteins associated with the same disease tend to localize in specific neighborhoods of the human interactome. However, the original DIAMOnD study revealed that disease proteins do not correspond solely to densely connected topological communities. Rather, the authors found that connectivity significance, rather than local connection density, was the most informative quantity for characterizing disease-protein interaction patterns.

The algorithm begins with a set of seed proteins, typically known disease-associated genes or proteins. In each iteration, DIAMOnD evaluates proteins connected to the current seed/module set and calculates the significance of each candidate's connection to the disease module. This significance is expressed as a p-value reflecting whether the observed number of links from a candidate protein to the current disease module is higher than expected, given the candidate’s overall degree in the interactome. This degree-aware formulation is important because it reduces the likelihood that highly connected hub proteins will be selected merely because they have many links in general.

After calculating the connectivity significance of the candidate proteins, DIAMOnD ranks them by p-value and adds the protein with the highest significance to the module. The module is updated, and the connectivity significance is recalculated with respect to the enlarged module. Then, the process is repeated. Thus, DIAMOnD expands the disease module one protein at a time. The order in which proteins are added can be interpreted as a ranking of candidate disease proteins based on their topology, where early additions are those most strongly connected to the known disease neighborhood.

Section S4.2: NeDRex Agent Tool TrustRank

TrustRank was originally introduced as a modification of PageRank for web-spam detection. According to the original TrustRank paper, the process begins with a small set of seed pages that are manually evaluated by experts [7]. Trust is then propagated through the link structure of the web graph to identify other pages that are likely reputable.

This concept can be applied to biomedical networks. If a small set of nodes is considered reliable or relevant, then nodes strongly connected to these trusted seeds may also be relevant. In the original formulation, the graph structure determines how trust flows from the seed set to other nodes. Nodes receive higher TrustRank values when they are more strongly reached through paths originating from trusted seeds. However, the propagation process limits how far and diffusely this trust spreads through the graph.

In the NeDRex implementation, TrustRank is applied to a heterogeneous biomedical network containing protein-protein and protein-drug associations [5]. The trusted seed nodes correspond to disease proteins or disease-module proteins. Trust is then propagated through the network, and drugs are ranked according to their connection to the trusted, disease-relevant seeds. The damping factor controls propagation behavior; lower values keep the ranking closer to the seed neighborhood, while higher values allow more exploratory propagation through the network.

Section S4.3: NeDRex Agent Tool Closeness Centrality

Closeness centrality is a classic network measure that ranks nodes based on their shortest path distances to other nodes [5]. In graph theory, a node has high closeness if it can reach other nodes through short paths. This node is considered centrally located because it is topologically close to many other parts of the network [5,8].

In NeDRex, this concept is adapted for drug repurposing. Instead of calculating the closeness of drugs to all nodes in the network, NeDRex calculates their closeness to selected protein seeds in a heterogeneous network of protein-protein and protein-drug associations [5]. The rationale is that drugs should be prioritized if they are close to the disease module or disease-associated proteins because these drugs may target molecular mechanisms near the disease-relevant network neighborhood.

Section S5: Knowledge Graph Agent

The **Knowledge Graph agent** (Fig. 4) is a strictly structured, KG centered question answering chain. Based on user input, the question is first mapped semantically to the fixed structure of the NeDRex KG and broken down into a list of questions instead of a question graph, which is typically used [9]. Each element in the list is communicated through an LLM invocation that specifies the corresponding node type in the KG. This invocation is also responsible for creating both the value and the subquery for each subquestion element. Furthermore, a Boolean value is assigned to indicate whether this node requires filtering at a later stage. Subsequently, each element in the question list that requires filtering is enriched with the top N nodes that are most similar to it. To accomplish this objective, the KG is searched using an embedding-based similarity query option, with the value and the subquestion as inputs. In the next step, the agent generates deterministic Cypher queries against Neo4j. The known KG schema, in conjunction with the type and edge restrictions embedded within the question graph, serves as the guiding principle for the creation process. This approach has been demonstrated to reduce the occurrence of hallucinations and enhance traceability [10,11]. Due to the prior enrichment, the LLM is capable of utilizing the user's value for filtering purposes and has access to the relevant IDs obtained from the previous embedding-based nodes matching. This reduction in error rate is a key benefit of

the proposed approach. If only the value used by the user is employed, the Cypher query itself may be accurate; however, it may not yield any results due to variations in spelling across the database, despite the inclusion of nodes and connections. By categorizing the elements in advance as to whether they require filtering, it is ensured that the LLM does not add filters that degrade the results. The agent has a fixed number of retries for the Cypher query creation, denoted as R. If the creation fails R times, the agent uses a fallback based on the GraphRAG principle [12]. In this case, the graph-guided retrieval steps are summarized, and only the condensed passages relevant to the graph structure are submitted to the LLM for generation.

Section S6: Research Agent

The **Research agent** is used in cases where NeDRex has limited or no information. We use human-like input to query scientific information, leveraging Semantic Scholar to identify relevant literature [13]. To enhance the precision and reliability of the search, an LLM is employed to parse the query into three distinct, more targeted inquiries. This approach of task decomposition not only improves the efficiency of the search process but also enhances the relevance of the retrieved results by encompassing a diverse range of queries [3].

Section S7: Finalize Agent

The **Finalize agent** synthesizes responses from the processing agents to generate, given the initial user query, a coherent final response in Markdown format, as commonly used in other popular solutions like ChatGPT. Based on the question and the information provided by the latest state, the agent will create a comprehensive answer. This answer maintains academic accuracy by citing sources and adhering to structured formats [14,15]. This systematic approach ensures that the synthesized answers are both informative and methodologically sound, thus supporting the node's goal of providing high-quality, well-structured results in the context of various fields [14]. A specialized output guardrail has been developed to address the prevalent issue of hallucinations in LLMs by employing hallucination verification [16,17]. To this end, the streamed response from the Finalize agent is reviewed paragraph-wise by another LLM to ensure consistency with the previous context and to eliminate incorrect content. This approach effectively overcomes the common limitations of fluent but factually inaccurate formulations [16,17].

Section S8: Knowledge Graph Evaluation on NeDRex KG

We used Gemini [18] to generate a diverse set of natural language questions relevant to the domain of biomedical knowledge represented in the Neo4j graph database. The questions were designed to cover a range of complexity, from simple entity lookups to more complex relationship traversals (e.g. “What drugs are indicated for treating 'COVID-19'?” vs “What are the genomic variants associated with disorders that affect gene expression in the skin epidermis?”). qwen2.5-coder:7b [19], an LLM specialized in code generation, was then tasked with translating each natural language question into a corresponding Cypher query. Each generated Cypher query was executed against the Neo4j database. Queries that resulted in syntax errors or failed to return any results were automatically discarded, ensuring that only syntactically correct and potentially meaningful queries were retained. A second LLM, specifically the deepseek-r1:70b model [20], was used to assess the semantic relatedness and biological plausibility of each question-Cypher query pair. This step aimed to filter out question-query pairs where the query, while executable, did not accurately capture the intent of the original question or produced results that were biologically nonsensical. Only those question-query pairs deemed semantically consistent and biologically meaningful by deepseek-r1:70b were included in the SSAD. Subsequently, a final refinement step was applied. During this step, the SSAD was reviewed, and any necessary corrections were made to ensure the integrity and quality of the test set.

Multimedia Appendix 1 References

1. Wang Q, Ding L, Cao Y, Tian Z, Wang S, Tao D, Guo L. Recursively summarizing enables long-term dialogue memory in large language models. Neurocomputing 2023 Aug 29;abs/2308.15022. doi: 10.48550/arXiv.2308.15022

2. Huang X, Liu W, Chen X, Wang X, Wang H, Lian D, Wang Y, Tang R, Chen E. Understanding the planning of LLM agents: A survey. arXiv [csAI]. 2024. Available from: http://arxiv.org/abs/2402.02716

3. Li A, Xie Y, Li S, Tsung F, Ding B, Li Y. Agent-Oriented Planning in Multi-Agent Systems. Int Conf Learn Represent 2024 Oct 3;abs/2410.02189. doi: 10.48550/arXiv.2410.02189

4. Adamowicz K, Maier A, Baumbach J, Blumenthal DB. Online in silico validation of disease and gene sets, clusterings or subnetworks with DIGEST. Brief Bioinform Oxford University Press (OUP); 2022 July 18;23(4):bbac247. PMID:35753693

5. Sadegh S, Skelton J, Anastasi E, Bernett J, Blumenthal DB, Galindez G, Salgado-Albarrán M, Lazareva O, Flanagan K, Cockell S, Nogales C, Casas AI, Schmidt HHHW, Baumbach J, Wipat A, Kacprowski T. Network medicine for disease module identification and drug repurposing with the NeDRex platform. Nat Commun Springer Science and Business Media LLC; 2021 Nov 25;12(1):6848. PMID:34824199

6. Ghiassian SD, Menche J, Barabási A-L. A DIseAse MOdule Detection (DIAMOnD) algorithm derived from a systematic analysis of connectivity patterns of disease proteins in the human interactome. PLoS Comput Biol Public Library of Science (PLoS); 2015 Apr;11(4):e1004120. PMID:25853560

7. Gyöngyi Z, Garcia-Molina H, Pedersen J. Combating Web Spam with TrustRank. Proceedings 2004 VLDB Conference Elsevier; 2004. p. 576–587. ISBN:9780120884698

8. Cohen E, Delling D, Pajor T, Werneck RF. Computing classic closeness centrality, at scale. arXiv [csDS]. 2014. doi: 10.48550/arXiv.1409.0035

9. Zheng W, Zhang M. Question answering over knowledge graphs via structural query patterns. arXiv [csAI]. 2019. Available from: http://arxiv.org/abs/1910.09760

10. Özsoy MG, Messallem L, Besga J, Minneci G. Text2Cypher: Bridging natural language and graph databases. ArXiv 2024 Dec 13;abs/2412.10064. doi: 10.48550/arXiv.2412.10064

11. Pusch L, Conrad TOF. Combining LLMs and Knowledge Graphs to reduce hallucinations in question answering. arXiv [csCL]. 2024. Available from: http://arxiv.org/abs/2409.04181

12. Han H, Wang Y, Shomer H, Guo K, Ding J, Lei Y, Halappanavar M, Rossi RA, Mukherjee S, Tang X, He Q, Hua Z, Long B, Zhao T, Shah N, Javari A, Xia Y, Tang J. Retrieval-augmented generation with graphs (GraphRAG). arXiv [csIR]. 2024. doi: 10.48550/arXiv.2501.00309

13. Fricke S. Semantic Scholar. J Med Libr Assoc University Library System, University of Pittsburgh; 2018 Jan 12;106(1). doi: 10.5195/jmla.2018.280

14. Bolaños F, Salatino A, Osborne F, Motta E. Artificial intelligence for literature reviews: opportunities and challenges. Artif Intell Rev Springer Science and Business Media LLC; 2024 Aug 17;57(10). doi: 10.1007/s10462-024-10902-3

15. Jeong S, Baek J, Cho S, Hwang SJ, Park JC. Adaptive-RAG: Learning to adapt retrieval-Augmented Large Language Models through Question complexity. arXiv [csCL]. 2024. Available from: http://arxiv.org/abs/2403.14403

16. Jeong C. A study on the implementation method of an agent-based advanced RAG system using Graph. arXiv [csAI] arXiv; 2024; doi: 10.48550/ARXIV.2407.19994

17. Yan S-Q, Gu J-C, Zhu Y, Ling Z-H. Corrective Retrieval Augmented Generation. arXiv [csCL]. 2024. Available from: http://arxiv.org/abs/2401.15884

18. Gemini Team Google: Rohan Anil, Borgeaud S, Alayrac J-B, Yu J, Soricut R, Schalkwyk J, Dai AM, Hauth A, Millican K, Silver D, Johnson M, Antonoglou I, Schrittwieser J, Glaese A, Chen J, Pitler E, Lillicrap T, Lazaridou A, Firat O, Molloy J, Isard M, Barham PR, Hennigan T, Lee B, Viola F, Reynolds M, Xu Y, Doherty R, Collins E, Meyer C, Rutherford E, Moreira E, Ayoub K, Goel M, Krawczyk J, Du C, Chi E, Cheng H-T, Ni E, Shah P, Kane P, Chan B, Faruqui M, Severyn A, Lin H, Li Y, Cheng Y, Ittycheriah A, Mahdieh M, Chen M, Sun P, Tran D, Bagri S, Lakshminarayanan B, Liu J, Orban A, Güra F, Zhou H, Song X, Boffy A, Ganapathy H, Zheng S, Choe H, Weisz Á, Zhu T, Lu Y, Gopal S, Kahn J, Kula M, Pitman J, Shah R, Taropa E, Al Merey M, Baeuml M, Chen Z, El Shafey L, Zhang Y, Sercinoglu O, Tucker G, Piqueras E, Krikun M, Barr I, Savinov N, Danihelka I, Roelofs B, White A, Andreassen A, von Glehn T, Yagati L, Kazemi M, Gonzalez L, Khalman M, Sygnowski J, Frechette A, Smith C, Culp L, Proleev L, Luan Y, Chen X, Lottes J, Schucher N, Lebron F, Rrustemi A, Clay N, Crone P, Kocisky T, Zhao J, Perz B, Yu D, Howard H, Bloniarz A, Rae JW, Lu H, Sifre L, Maggioni M, Alcober F, Garrette D, Barnes M, Thakoor S, Austin J, Barth-Maron G, Wong W, Joshi R, Chaabouni R, Fatiha D, Ahuja A, Tomar GS, Senter E, Chadwick M, Kornakov I, Attaluri N, Iturrate I, Liu R, Li Y, Cogan S, Chen J, Jia C, Gu C, Zhang Q, Grimstad J, Hartman AJ, Garcia X, Pillai TS, Devlin J, Laskin M, de Las Casas D, Valter D, Tao C, Blanco L, Badia AP, Reitter D, Chen M, Brennan J, Rivera C, Brin S, Iqbal S, Surita G, Labanowski J, Rao A, Winkler S, Parisotto E, Gu Y, Olszewska K, Addanki R, Miech A, Louis A, Teplyashin D, Brown G, Catt E, Balaguer J, Xiang J, Wang P, Ashwood Z, Briukhov A, Webson A, Ganapathy S, Sanghavi S, Kannan A, Chang M-W, Stjerngren A, Djolonga J, Sun Y, Bapna A, Aitchison M, Pejman P, Michalewski H, Yu T, Wang C, Love J, Ahn J, Bloxwich D, Han K, Humphreys P, Sellam T, Bradbury J, Godbole V, Samangooei S, Damoc B, Kaskasoli A, Arnold SMR, Vasudevan V, Agrawal S, Riesa J, Lepikhin D, Tanburn R, Srinivasan S, Lim H, Hodkinson S, Shyam P, Ferret J, Hand S, Garg A, Le Paine T, Li J, Li Y, Giang M, Neitz A, Abbas Z, York S, Reid M, Cole E, Chowdhery A, Das D, Rogozińska D, Nikolaev V, Sprechmann P, Nado Z, Zilka L, Prost F, He L, Monteiro M, Mishra G, Welty C, Newlan J, Jia D, Allamanis M, Hu CH, de Liedekerke R, Gilmer J, Saroufim C, Rijhwani S, Hou S, Shrivastava D, Baddepudi A, Goldin A, Ozturel A, Cassirer A, Xu Y, Sohn D, Sachan D, Amplayo RK, Swanson C, Petrova D, Narayan S, Guez A, Brahma S, Landon J, Patel M, Zhao R, Villela K, Wang L, Jia W, Rahtz M, Giménez M, Yeung L, Keeling J, Georgiev P, Mincu D, Wu B, Haykal S, Saputro R, Vodrahalli K, Qin J, Cankara Z, Sharma A, Fernando N, Hawkins W, Neyshabur B, Kim S, Hutter A, Agrawal P, Castro-Ros A, van den Driessche G, Wang T, Yang F, Chang S-Y, Komarek P, McIlroy R, Lučić M, Zhang G, Farhan W, Sharman M, Natsev P, Michel P, Bansal Y, Qiao S, Cao K, Shakeri S, Butterfield C, Chung J, Rubenstein PK, Agrawal S, Mensch A, Soparkar K, Lenc K, Chung T, Pope A, Maggiore L, Kay J, Jhakra P, Wang S, Maynez J, Phuong M, Tobin T, Tacchetti A, Trebacz M, Robinson K, Katariya Y, Riedel S, Bailey P, Xiao K, Ghelani N, Aroyo L, Slone A, Houlsby N, Xiong X, Yang Z, Gribovskaya E, Adler J, Wirth M, Lee L, Music Li, Kagohara T, Pavagadhi J, Bridgers S, Bortsova A, Ghemawat S, Ahmed Z, Liu T, Powell R, Bolina V, Iinuma M, Zablotskaia P, Besley J, Chung D-W, Dozat T, Comanescu R, Si X, Greer J, Su G, Polacek M, Kaufman RL, Tokumine S, Hu H, Buchatskaya E, Miao Y, Elhawaty M, Siddhant A, Tomasev N, Xing J, Greer C, Miller H, Ashraf S, Roy A, Zhang

Z, Ma A, Filos A, Besta M, Blevins R, Klimenko T, Yeh C-K, Changpinyo S, Mu J, Chang O, Pajarskas M, Muir C, Cohen V, Le Lan C, Haridasan K, Marathe A, Hansen S, Douglas S, Samuel R, Wang M, Austin S, Lan C, Jiang J, Chiu J, Lorenzo JA, Sjösund LL, Cevey S, Gleicher Z, Avrahami T, Boral A, Srinivasan H, Selo V, May R, Aisopos K, Hussenot L, Soares LB, Baumli K, Chang MB, Recasens A, Caine B, Pritzel A, Pavetic F, Pardo F, Gergely A, Frye J, Ramasesh V, Horgan D, Badola K, Kassner N, Roy S, Dyer E, Campos VC, Tomala A, Tang Y, El Badawy D, White E, Mustafa B, Lang O, Jindal A, Vikram S, Gong Z, Caelles S, Hemsley R, Thornton G, Feng F, Stokowiec W, Zheng C, Thacker P, Ünlü Ç, Zhang Z, Saleh M, Svensson J, Bileschi M, Patil P, Anand A, Ring R, Tsihlas K, Vezer A, Selvi M, Shevlane T, Rodriguez M, Kwiatkowski T, Daruki S, Rong K, Dafoe A, FitzGerald N, Gu-Lemberg K, Khan M, Hendricks LA, Pellat M, Feinberg V, Cobon-Kerr J, Sainath T, Rauh M, Hashemi SH, Ives R, Hasson Y, Noland E, Cao Y, Byrd N, Hou L, Wang Q, Sottiaux T, Paganini M, Lespiau J-B, Moufarek A, Hassan S, Shivakumar K, van Amersfoort J, Mandhane A, Joshi P, Goyal A, Tung M, Brock A, Sheahan H, Misra V, Li C, Rakićević N, Dehghani M, Liu F, Mittal S, Oh J, Noury S, Sezener E, Huot F, Lamm M, De Cao N, Chen C, Mudgal S, Stella R, Brooks K, Vasudevan G, Liu C, Chain M, Melinkeri N, Cohen A, Wang V, Seymore K, Zubkov S, Goel R, Yue S, Krishnakumaran S, Albert B, Hurley N, Sano M, Mohananey A, Joughin J, Filonov E, Kępa T, Eldawy Y, Lim J, Rishi R, Badiezadegan S, Bos T, Chang J, Jain S, Padmanabhan SGS, Puttagunta S, Krishna K, Baker L, Kalb N, Bedapudi V, Kurzrok A, Lei S, Yu A, Litvin O, Zhou X, Wu Z, Sobell S, Siciliano A, Papir A, Neale R, Bragagnolo J, Toor T, Chen T, Anklin V, Wang F, Feng R, Gholami M, Ling K, Liu L, Walter J, Moghaddam H, Kishore A, Adamek J, Mercado T, Mallinson J, Wandekar S, Cagle S, Ofek E, Garrido G, Lombriser C, Mukha M, Sun B, Mohammad HR, Matak J, Qian Y, Peswani V, Janus P, Yuan Q, Schelin L, David O, Garg A, He Y, Duzhyi O, Älgmyr A, Lottaz T, Li Q, Yadav V, Xu L, Chinien A, Shivanna R, Chuklin A, Li J, Spadine C, Wolfe T, Mohamed K, Das S, Dai Z, He K, von Dincklage D, Upadhyay S, Maurya A, Chi L, Krause S, Salama K, Rabinovitch PG, Pavan KRM, Selvan A, Dektiarev M, Ghiasi G, Guven E, Gupta H, Liu B, Sharma D, Shtacher IH, Paul S, Akerlund O, Aubet F-X, Huang T, Zhu C, Zhu E, Teixeira E, Fritze M, Bertolini F, Marinescu L-E, Bölle M, Paulus D, Gupta K, Latkar T, Chang M, Sanders J, Wilson R, Wu X, Tan Y-X, Thiet LN, Doshi T, Lall S, Mishra S, Chen W, Luong T, Benjamin S, Lee J, Andrejczuk E, Rabiej D, Ranjan V, Styrc K, Yin P, Simon J, Harriott MR, Bansal M, Robsky A, Bacon G, Greene D, Mirylenka D, Zhou C, Sarvana O, Goyal A, Andermatt S, Siegler P, Horn B, Israel A, Pongetti F, Chen C-W “louis,” Selvatici M, Silva P, Wang K, Tolins J, Guu K, Yogev R, Cai X, Agostini A, Shah M, Nguyen H, Donnaile NÓ, Pereira S, Friso L, Stambler A, Kurzrok A, Kuang C, Romanikhin Y, Geller M, Yan ZJ, Jang K, Lee C-C, Fica W, Malmi E, Tan Q, Banica D, Balle D, Pham R, Huang Y, Avram D, Shi H, Singh J, Hidey C, Ahuja N, Saxena P, Dooley D, Potharaju SP, O’Neill E, Gokulchandran A, Foley R, Zhao K, Dusenberry M, Liu Y, Mehta P, Kotikalapudi R, Safranek-Shrader C, Goodman A, Kessinger J, Globen E, Kolhar P, Gorgolewski C, Ibrahim A, Song Y, Eichenbaum A, Brovelli T, Potluri S, Lahoti P, Baetu C, Ghorbani A, Chen C, Crawford A, Pal S, Sridhar M, Gurita P, Mujika A, Petrovski I, Cedoz P-L, Li C, Chen S, Santo ND, Goyal S, Punjabi J, Kappaganthu K, Kwak C, Pallavi LV, Velury S, Choudhury H, Hall J, Shah P, Figueira R, Thomas M, Lu M, Zhou T, Kumar C, Jurdi T, Chikkerur S, Ma Y, Yu A, Kwak S, Ähdel V, Rajayogam S, Choma T, Liu F, Barua A, Ji C, Park JH, Hellendoorn V, Bailey A, Bilal T, Zhou H, Khatir M, Sutton C, Rzadkowski W, Macintosh F, Vij R, Shagin K, Medina P, Liang C, Zhou J, Shah P, Bi Y, Dankovics A, Banga S, Lehmann S, Bredesen M, Lin Z, Hoffmann JE, Lai J, Chung R, Yang K, Balani N, Bražinskas A, Sozanschi A, Hayes M, Alcalde HF, Makarov P, Chen W, Stella A, Snijders L, Mandl M, Kärrman A, Nowak P, Wu X, Dyck A, Vaidyanathan K, Raghavender R, Mallet J, Rudominer M, Johnston E, Mittal S, Udathu A, Christensen J, Verma V, Irving Z, Santucci A, Elsayed G, Davoodi E, Georgiev M, Tenney I, Hua N, Cideron G, Leurent E, Alnahlawi M, Georgescu I, Wei N, Zheng I, Scandinaro D, Jiang H, Snoek J, Sundararajan M, Wang X, Ontiveros Z, Karo I, Cole J, Rajashekhar V, Tumeh L, Ben-David E, Jain R, Uesato J, Datta R, Bunyan O,

Wu S, Zhang J, Stanczyk P, Zhang Y, Steiner D, Naskar S, Azzam M, Johnson M, Paszke A, Chiu C-C, Elias JS, Mohiuddin A, Muhammad F, Miao J, Lee A, Vieillard N, Park J, Zhang J, Stanway J, Garmon D, Karmarkar A, Dong Z, Lee J, Kumar A, Zhou L, Evens J, Isaac W, Irving G, Loper E, Fink M, Arkatkar I, Chen N, Shafran I, Petrychenko I, Chen Z, Jia J, Levskaya A, Zhu Z, Grabowski P, Mao Y, Magni A, Yao K, Snaider J, Casagrande N, Palmer E, Suganthan P, Castaño A, Giannoumis I, Kim W, Rybiński M, Sreevatsa A, Prendki J, Soergel D, Goedeckemeyer A, Gierke W, Jafari M, Gaba M, Wiesner J, Wright DG, Wei Y, Vashisht H, Kulizhskaya Y, Hoover J, Le M, Li L, Iwuanyanwu C, Liu L, Ramirez K, Khorlin A, Cui A, Tian LIN, Wu M, Aguilar R, Pallo K, Chakladar A, Perng G, Abellan EA, Zhang M, Dasgupta I, Kushman N, Penchev I, Repina A, Wu X, van der Weide T, Ponnapalli P, Kaplan C, Simsa J, Li S, Dousse O, Yang F, Piper J, Ie N, Pasumarthi R, Lintz N, Vijayakumar A, Andor D, Valenzuela P, Lui M, Paduraru C, Peng D, Lee K, Zhang S, Greene S, Nguyen DD, Kurylowicz P, Hardin C, Dixon L, Janzer L, Choo K, Feng Z, Zhang B, Singhal A, Du D, McKinnon D, Antropova N, Bolukbasi T, Keller O, Reid D, Finchelstein D, Raad MA, Crocker R, Hawkins P, Dadashi R, Gaffney C, Franko K, Bulanova A, Leblond R, Chung S, Askham H, Cobo LC, Xu K, Fischer F, Xu J, Sorokin C, Alberti C, Lin C-C, Evans C, Dimitriev A, Forbes H, Banarse D, Tung Z, Omernick M, Bishop C, Sterneck R, Jain R, Xia J, Amid E, Piccinno F, Wang X, Banzal P, Mankowitz DJ, Polozov A, Krakovna V, Brown S, Bateni M, Duan D, Firoiu V, Thotakuri M, Natan T, Geist M, Girgin ST, Li H, Ye J, Roval O, Tojo R, Kwong M, Lee-Thorp J, Yew C, Sinopalnikov D, Ramos S, Mellor J, Sharma A, Wu K, Miller D, Sonnerat N, Vnukov D, Greig R, Beattie J, Caveness E, Bai L, Eisenschlos J, Korchemniy A, Tsai T, Jasarevic M, Kong W, Dao P, Zheng Z, Liu F, Yang F, Zhu R, Teh TH, Sanmiya J, Gladchenko E, Trdin N, Toyama D, Rosen E, Tavakkol S, Xue L, Elkind C, Woodman O, Carpenter J, Papamakarios G, Kemp R, Kafle S, Grunina T, Sinha R, Talbert A, Wu D, Owusu-Afriyie D, Du C, Thornton C, Pont-Tuset J, Narayana P, Li J, Fatehi S, Wieting J, Ajmeri O, Uria B, Ko Y, Knight L, Héliou A, Niu N, Gu S, Pang C, Li Y, Levine N, Stolovich A, Santamaria-Fernandez R, Goenka S, Yustalim W, Strudel R, Elqursh A, Deck C, Lee H, Li Z, Levin K, Hoffmann R, Holtmann-Rice D, Bachem O, Arora S, Koh C, Yeganeh SH, Põder S, Tariq M, Sun Y, Ionita L, Seyedhosseini M, Tafti P, Liu Z, Gulati A, Liu J, Ye X, Chrzaszcz B, Wang L, Sethi N, Li T, Brown B, Singh S, Fan W, Parisi A, Stanton J, Koverkathu V, Choquette-Choo CA, Li Y, Lu TJ, Ittycheriah A, Shroff P, Varadarajan M, Bahargam S, Willoughby R, Gaddy D, Desjardins G, Cornero M, Robenek B, Mittal B, Albrecht B, Shenoy A, Moiseev F, Jacobsson H, Ghaffarkhah A, Rivière M, Walton A, Crepy C, Parrish A, Zhou Z, Farabet C, Radebaugh C, Srinivasan P, van der Salm C, Fidjeland A, Scellato S, Latorre-Chimoto E, Klimczak-Plucińska H, Bridson D, de Cesare D, Hudson T, Mendolicchio P, Walker L, Morris A, Mauger M, Guseynov A, Reid A, Odoom S, Loher L, Cotruta V, Yenugula M, Grewe D, Petrushkina A, Duerig T, Sanchez A, Yadlowsky S, Shen A, Globerson A, Webb L, Dua S, Li D, Bhupatiraju S, Hurt D, Qureshi H, Agarwal A, Shani T, Eyal M, Khare A, Belle SR, Wang L, Tekur C, Kale MS, Wei J, Sang R, Saeta B, Liechty T, Sun Y, Zhao Y, Lee S, Nayak P, Fritz D, Vuyyuru MR, Aslanides J, Vyas N, Wicke M, Ma X, Eltyshev E, Martin N, Cate H, Manyika J, Amiri K, Kim Y, Xiong X, Kang K, Luisier F, Tripuraneni N, Madras D, Guo M, Waters A, Wang O, Ainslie J, Baldridge J, Zhang H, Pruthi G, Bauer J, Yang F, Mansour R, Gelman J, Xu Y, Polovets G, Liu J, Cai H, Chen W, Sheng X, Xue E, Ozair S, Angermueller C, Li X, Sinha A, Wang W, Wiesinger J, Koukoumidis E, Tian Y, Iyer A, Gurumurthy M, Goldenson M, Shah P, Blake MK, Yu H, Urbanowicz A, Palomaki J, Fernando C, Durden K, Mehta H, Momchev N, Rahimtoroghi E, Georgaki M, Raul A, Ruder S, Redshaw M, Lee J, Zhou D, Jalan K, Li D, Hechtman B, Schuh P, Nasr M, Milan K, Mikulik V, Franco J, Green T, Nguyen N, Kelley J, Mahendru A, Hu A, Howland J, Vargas B, Hui J, Bansal K, Rao V, Ghiya R, Wang E, Ye K, Sarr JM, Preston MM, Elish M, Li S, Kaku A, Gupta J, Pasupat I, Juan D-C, Someswar M, M. T, Chen X, Amini A, Fabrikant A, Chu E, Dong X, Muthal A, Buthpitiya S, Jauhari S, Hua N, Khandelwal U, Hitron A, Ren J, Rinaldi L, Drath S, Dabush A, Jiang N-J, Godhia H, Sachs U, Chen A, Fan Y, Taitelbaum H, Noga H, Dai Z,

Wang J, Liang C, Hamer J, Ferng C-S, Elkind C, Atias A, Lee P, Listík V, Carlen M, van de Kerkhof J, Pikus M, Zaher K, Müller P, Zykova S, Stefanec R, Gatsko V, Hirnschall C, Sethi A, Xu XF, Ahuja C, Tsai B, Stefanoiu A, Feng B, Dhandhania K, Katyal M, Gupta A, Parulekar A, Pitta D, Zhao J, Bhatia V, Bhavnani Y, Alhadlaq O, Li X, Danenberg P, Tu D, Pine A, Filippova V, Ghosh A, Limonchik B, Urala B, Lanka CK, Clive D, Sun Y, Li E, Wu H, Hongtongsak K, Li I, Thakkar K, Omarov K, Majmundar K, Alverson M, Kucharski M, Patel M, Jain M, Zabelin M, Pelagatti P, Kohli R, Kumar S, Kim J, Sankar S, Shah V, Ramachandruni L, Zeng X, Bariach B, Weidinger L, Vu T, Andreev A, He A, Hui K, Kashem S, Subramanya A, Hsiao S, Hassabis D, Kavukcuoglu K, Sadovsky A, Le Q, Strohman T, Wu Y, Petrov S, Dean J, Vinyals O. Gemini: A family of highly capable multimodal models. arXiv [csCL]. 2023. doi: 10.48550/arXiv.2312.11805

19. Hui B, Yang J, Cui Z, Yang J, Liu D, Zhang L, Liu T, Zhang J, Yu B, Lu K, Dang K, Fan Y, Zhang Y, Yang A, Men R, Huang F, Zheng B, Miao Y, Quan S, Feng Y, Ren X, Ren X, Zhou J, Lin J. Qwen2.5-Coder Technical Report. arXiv [csCL]. 2024. doi: 10.48550/arXiv.2409.12186

20. Bi D-AX, Chen D, Chen G, Chen S, Dai D, Deng C, Ding H, Dong K, Du Q, Fu Z, Gao H, Gao K, Gao W, Ge R, Guan K, Guo D, Guo J, Hao G, Hao Z, He Y, Hu W, Huang P, Li E, Li G, Li J, Li Y, Li YK, Liang W, Lin F, Liu AX, Liu B, Liu W, Liu X, Liu X, Liu Y, Lu H, Lu S, Luo F, Ma S, Nie X, Pei T, Piao Y, Qiu J, Qu H, Ren T, Ren Z, Ruan C, Sha Z, Shao Z, Song J, Su X, Sun J, Sun Y, Tang M, Wang B, Wang P, Wang S, Wang Y, Wang Y, Wu T, Wu Y, Xie X, Xie Z, Xie Z, Xiong Y, Xu H, Xu RX, Xu Y, Yang D, You Y, Yu S, Yu X, Zhang B, Zhang H, Zhang L, Zhang L, Zhang M, Zhang M, Zhang W, Zhang Y, Zhao C, Zhao Y, Zhou S, Zhou S, Zhu Q, Zou Y. DeepSeek LLM: Scaling open-source language models with longtermism. arXiv [csCL]. 2024. doi: 10.48550/arXiv.2401.02954
