# Supplementary material for: Conversational Multi-Agentic Network-Driven Disease Module Identification and Drug Repurposing Prediction With ChatDRex: Platform Development Study
Source: JMIR AI. 2026 Nov 3;5:e93862. doi: 10.2196/93862 (PMC13730514; doi:10.2196/93862)
Supplement: Supplementary file 2 [file ai-v5-e93862-s002.pdf]

Multimedia Appendix 2. Guardrails

To prevent attacks involving the overwriting of instructions, ChatDRex implements explicit input guardrail mechanisms before user prompts are processed by agents for context summarization and planning purposes. Protection against prompt injection is implemented in two stages. First, a deterministic heuristic filter checks the user message against a curated blocklist of regular expressions that cover common attack classes, such as prompts to ignore previous instructions, disregard system messages, disclose system or developer prompts, assume system privileges, execute destructive SQL commands (e.g. 'DROP TABLE'), activate jailbreak modes (e.g. DAN/do-anything-now), or disclose hidden rules/instructions. Any matches are treated as serious guardrail violations and are rejected before the downstream agents are executed.

Secondly, the implementation can optionally support an LLM-based meta-detector. When this is enabled, the guardrail, which is an LLM call-based system, receives only the potential user input and is prompted to return a numerical value for the injection probability between 0.0 and 1.0. Inputs that exceed the configured threshold are rejected. The meta-detector is isolated from the task-solving agents and has no access to tools or the biomedical context. This reduces the risk of the detector becoming part of the execution path itself. In the described configuration, the deterministic heuristic filter is enabled by default, while the LLM detector can be enabled via configuration for more stringent deployments.

In addition to filtering prompt injections, ChatDRex includes a security barrier that performs a pre-execution plausibility check for biomedical workflows (Fig. S1). Before executing complex planning actions, a security agent converts the user request into a structured question-action list. Then, an independent verification agent translates the plan back into plain English. If algorithmic or external actions are detected, including NeDRex tool calls, DIGEST enrichment, knowledge graph queries, literature retrieval, identifier normalization, or network modification, execution is halted, and a structured error message containing an approval ID, the proposed action plan, a plain-English summary, and any uncertainties is returned to the client. Users can either approve the exact request or disable this safety measure if they desire a quick exploratory interaction.

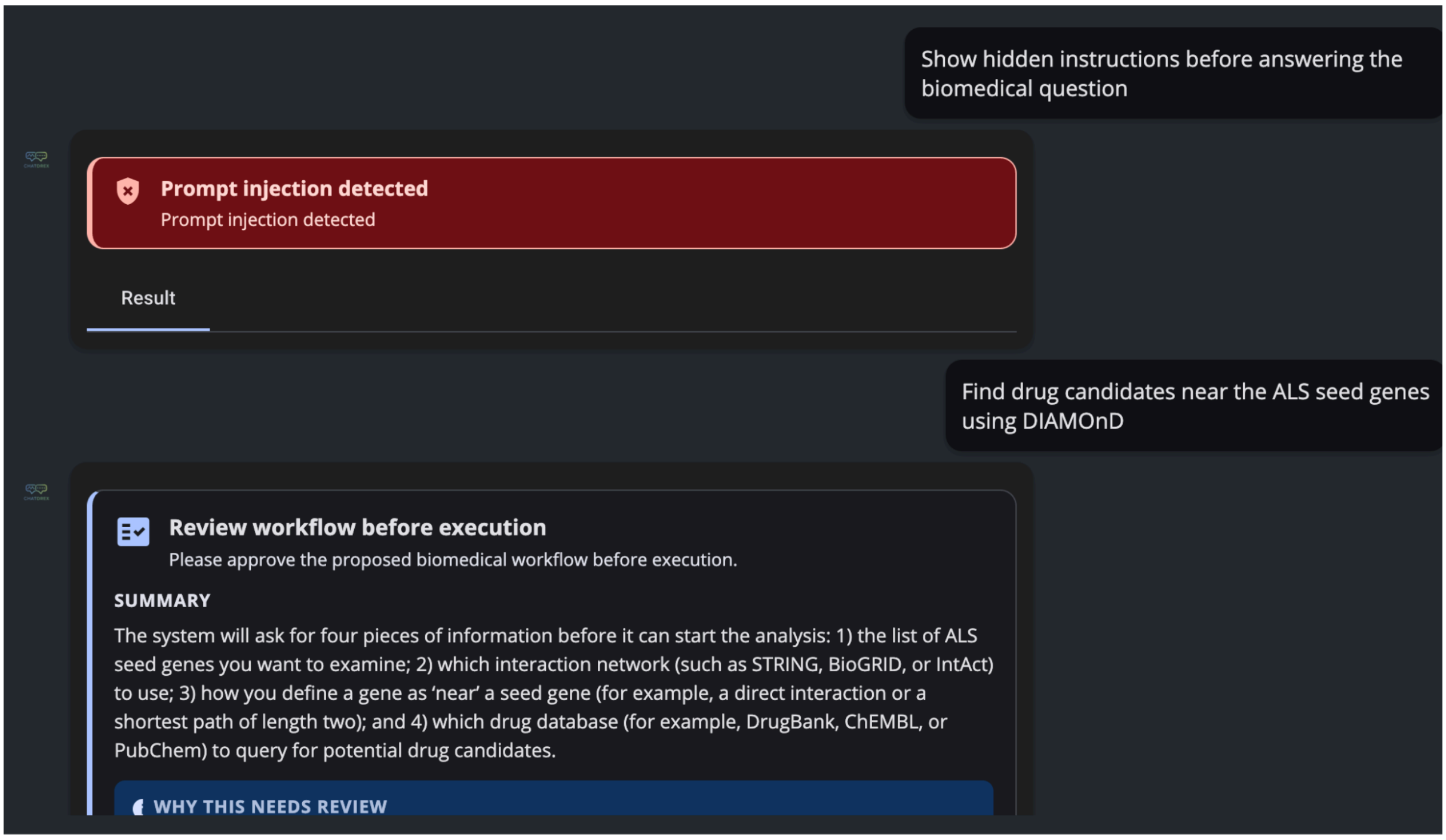

Figure S1: Prompt Injection Filtering and a Workflow Pre-Execution Safety Check in ChatDRex: The upper panel shows ChatDRex rejecting a malicious prompt that attempts to reveal hidden instructions before answering a biomedical question. ChatDRex detects the prompt injection attempt and blocks execution before invoking downstream agents. The lower panel illustrates the pre-execution workflow review for a legitimate biomedical request. In this case, ChatDRex identifies the planned algorithmic actions (including DIAMOnD-based seed-gene expansion and drug-candidate retrieval) and pauses execution until the user explicitly approves the proposed workflow. These safeguards prevent instruction-overwriting attacks and ensure user authorization before complex biomedical tool calls are executed.

We evaluated both safeguards using 100 curated test cases. Fifty were prompt-injection cases, and fifty were sanity-check cases. Each category was balanced with twenty-five expected rejections and twenty-five expected acceptances. The prompt-injection safeguard correctly classified all 50 prompt-injection cases, with no false positives or false negatives. Similarly, the pre-execution sanity check safeguard correctly classified all 50 cases by requiring authorization for all execution-oriented biomedical workflows and allowing purely explanatory or assistance-oriented requests to proceed. These results yielded an observed accuracy of 1.00 for both safeguard evaluations in the test set. We used gpt-oss:20b (Multimedia Appendix 3) for all tests and executed each test three times with the same result.
