# Supplementary material for: Conversational Multi-Agentic Network-Driven Disease Module Identification and Drug Repurposing Prediction With ChatDRex: Platform Development Study
Source: JMIR AI. 2026 Nov 3;5:e93862. doi: 10.2196/93862 (PMC13730514; doi:10.2196/93862)
Supplement: Supplementary file 3 [file ai-v5-e93862-s003.pdf]

Multimedia Appendix 3. Model Parameter

Table S1: ChatDRex LLM Model Parameter: These are the model parameters used in this manuscript. The sampling and context parameters used during model execution are reported. Values marked as "model default" were defined by the respective Ollama model package. Values marked as "Ollama default" correspond to generic Ollama runtime defaults used when no model-specific value was defined. The num_ctx column indicates whether the context window was configured, "default" means no custom context length was set. For qwen2.5-coder:latest, num_ctx was set to 25,000 tokens.

**Provider**	**Model**	**Temperature**	**Top-p**	**Top-k**	**num_ctx**
Ollama / self-hosted	gpt-oss:20b	1 (model default)	0.9 (ollama default)	40 (ollama default)	default
Ollama / self-hosted	nemotron-3-nano:4b	1 (model default)	1 (model default)	40 (ollama default)	default
Ollama / self-hosted	qwen3.6:27b	1 (model default)	0.95 (model default)	20 (model default)	default
Ollama / self-hosted	gemma4:e4b	1 (default)	0.95 (model default)	64 (model default)	default
Ollama / self-hosted	qwen2.5-coder:7b	0.8 (ollama default)	0.9 (ollama default)	40 (ollama default)	25000
Ollama / self-hosted	deepseek-r1:70b	0.8 (ollama default)	0.9 (ollama default)	40 (ollama default)	default

All language models used in this manuscript were executed through the same self-hosted Ollama environment with their default generation parameters. Table S1 summarizes these settings, distinguishing between those inherited from the respective Ollama model package and those inherited from Ollama's runtime defaults. These settings were applied consistently throughout the manuscript, including in interactive uses, system-level examples, and evaluation experiments, unless an exception is explicitly stated.
