# Supplementary material for: Conversational Multi-Agentic Network-Driven Disease Module Identification and Drug Repurposing Prediction With ChatDRex: Platform Development Study
Source: JMIR AI. 2026 Nov 3;5:e93862. doi: 10.2196/93862 (PMC13730514; doi:10.2196/93862)
Supplement: Supplementary file 4 [file ai-v5-e93862-s004.pdf]

Multimedia Appendix 4. Use Cases

Screenshots and response excerpts in Table S1 - Table S5 have been cropped and, where indicated, truncated for readability and presentation. The screenshots and response excerpts in Tables S1 - Table S5 have been truncated (removing the end of the final ChatDrex answer) where necessary for presentation. No editorial changes have been made. The interface-specific styling has been preserved. This does not reflect the capitalization used in the manuscript text. These presentation adjustments do not modify the underlying tool execution or analytical results.

An example ChatDRex workflow for DIGEST-Set enrichment analysis, including user input, planning decisions, tool calls, retrieved enrichment results, final response generation, and visualization output, is provided in Table S1 and is available online under the tab "Use Cases" identifiable by the respective user input (ID: ed459d5f-29ad-46a1-a4dd-da4c67781378 [1]).

Table S1: Functional Enrichment Analysis Workflow and User View in ChatDRex: This table illustrates the key agent workflow steps involved in performing a functional coherence analysis using ChatDRex, alongside corresponding snippets of the user interface. The user initiates the analysis by providing a set of genes, and ChatDRex handles the routing of the query, interaction with DIGEST-Set, and generation of a human-readable summary of the results, including a visualization of enriched pathways. While DIGEST's main purpose is not enrichment analysis, but coherence evaluation, it outputs enriched terms, as part of the process, which ChatDRex can build its answer on.

Workflow Step	Description
User Input	"*Can you tell me which pathways or functions are enriched for the genes APOE, APP, PSEN1, PSEN2, and SORL1?*"
Decisions	ACTION: Planning<br>ACTION: DIGEST<br>REASON: User requests pathway/function enrichment for specified gene set.<br>SUB-TASK: Run DIGEST on the genes APOE, APP, PSEN1, PSEN2, SORL1.<br>ACTION: Finalize<br>REASON: DIGEST result already provided functional coherence validation; no further actions needed.<br>SUB-TASK: Produce final summary and next-step recommendations based on gathered results.

<table>
<tr>
<td>Tool Interaction</td>
<td>
<table>
<tr><th>#</th><th>TOOL</th><th>INPUT</th><th>OUTPUT</th></tr>
<tr><td>1</td><td>MyGene</td><td>APOE, APP, PSEN1, PSEN2, SORL1</td><td>5 of 5 mapped
APOE → 348 (APOE)
APP → 351 (APP)
PSEN1 → 5663 (PSEN1) +2 more</td></tr>
<tr><td>2</td><td>DIGEST-Set</td><td>348, 351, 5663, 5664, 6653</td><td>Coherent in 4 of 4 databases
GO.CC (p = 1.0e-3)
KEGG (p = 1.0e-3)
GO.BP (p = 0.007) +1 more</td></tr>
</table>
</td>
</tr>
<tr>
<td>Response (truncated)</td>
<td>
DIGEST functional coherence analysis results for the gene set APOE, APP, PSEN1, PSEN2, SORL1, displaying KEGG and multi-category GO findings with empirical p-values.

The functional-coherence assessment evaluated the overlap among APOE, APP, PSEN1, PSEN2, and SORL1 across several annotation resources, providing empirical P-values and identifying the most frequently shared terms for each database DIGEST.

Gene Ontology Cellular Component (GO.CC)

The GO CC analysis yielded a highly significant coherence with an empirical P-value of 0.000999, indicating strong functional agreement across all five genes, and identified several subcellular structures that were consistently annotated DIGEST.

Endoplasmic reticulum (GO:0005783): 5 genes DIGEST
Golgi apparatus (GO:0005794): 5 genes DIGEST
Plasma membrane (GO:0005886): 5 genes DIGEST
Membrane (GO:0016020): 5 genes DIGEST
Early endosome (GO:0005769): 4 genes DIGEST

KEGG Pathways

The KEGG pathway analysis also showed significant coherence (P = 0.000999) for four of the five genes and highlighted several disease-related pathways, most notably Alzheimer’s disease, that are recurrent across this gene set DIGEST.

Alzheimer disease (hsa05010): 4 genes DIGEST

Empirical p-values and formatting in this example reproduce the raw ChatDRex tool output verbatim.
</td>
</tr>
<tr>
<td>Visualization</td>
<td>Functional coherence of the gene set according to the annotation database.</td>
</tr>
</table>

	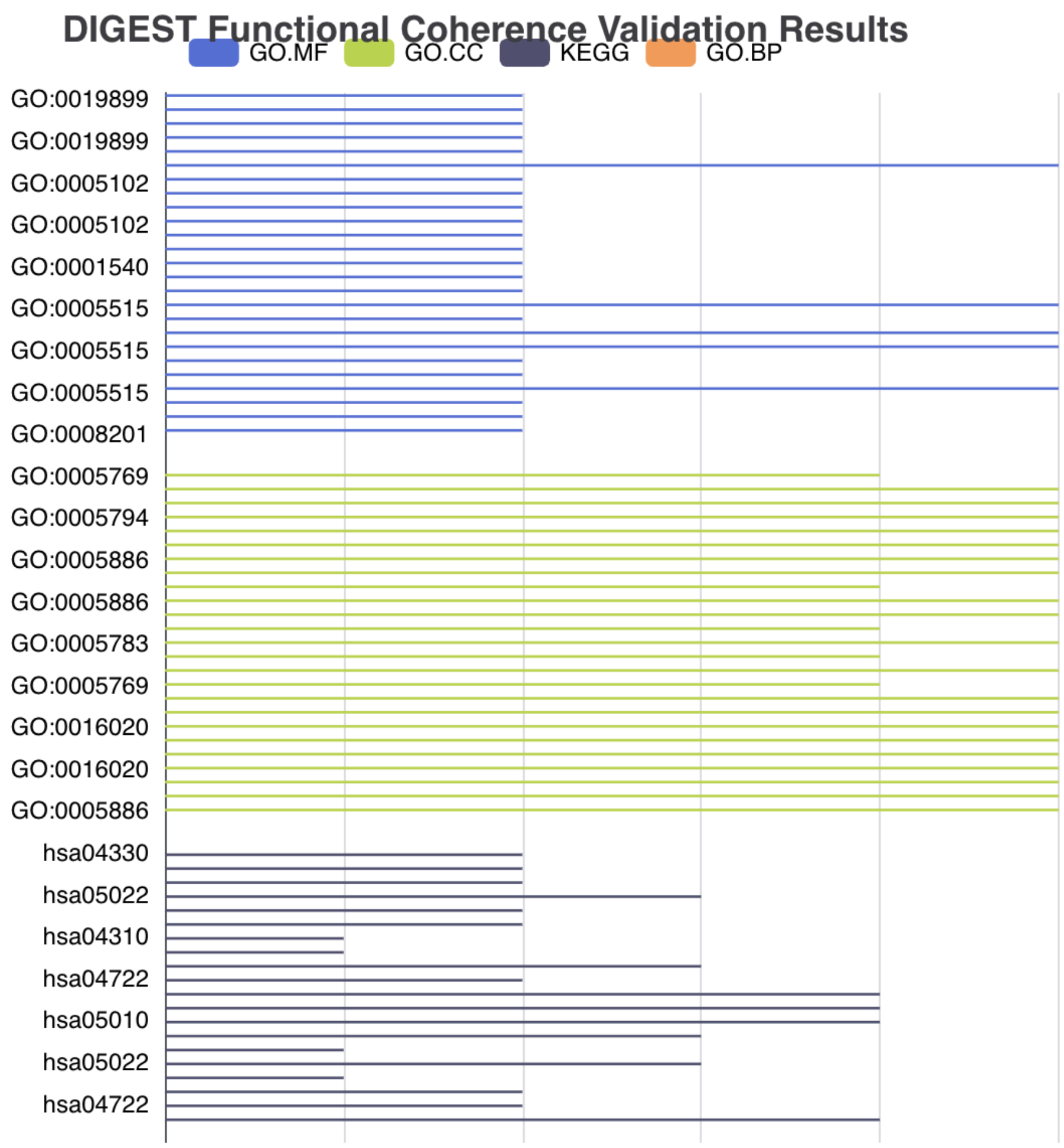<br>Due to space constraints, the result has been shortened here. Hovering over the text will display further information.<br>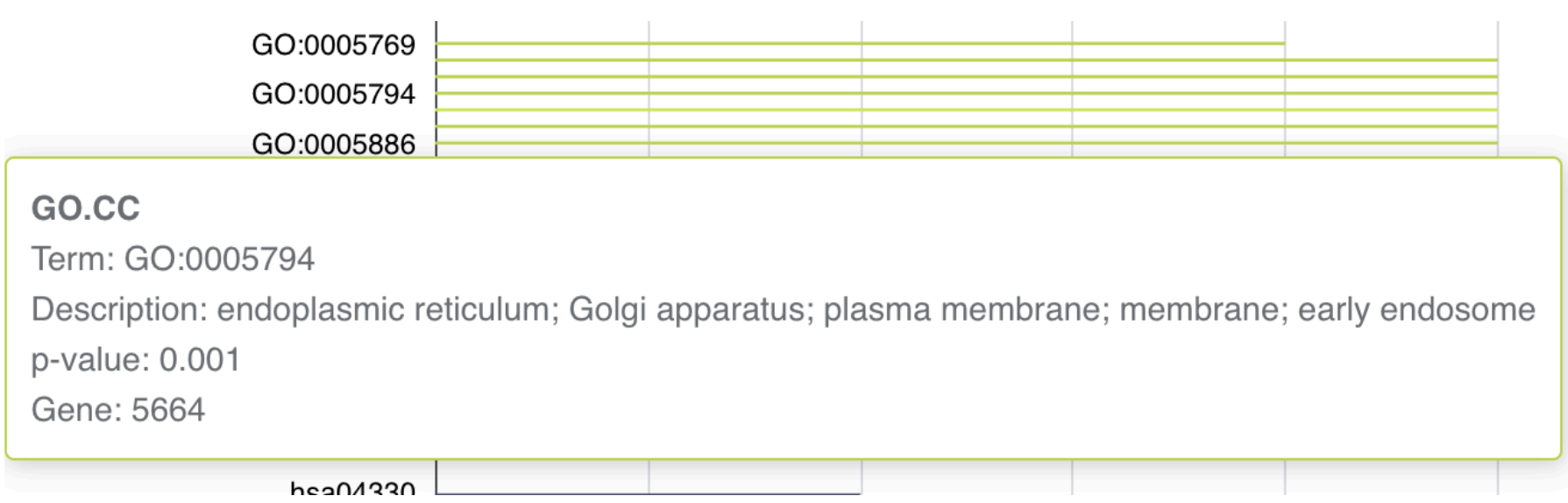

An example ChatDRex literature search workflow, including the agent planning steps, Semantic Scholar retrieval, user-facing interface snippets, and a final summary with observed limitations and inaccuracies, is provided in Table S2 and is available online under the tab "Use Cases" identifiable by the respective user input (ID: 18194734-7bd2-4d9e-abb7-f976c174b5a6 [2]).

Table S2: Literature Search Workflow and User View in ChatDRex - An example with limitations: This table illustrates the agent workflow steps involved in a literature search using ChatDRex, alongside snippets of the user interface. While the system attempts to

answer the user's query about new drugs for Alzheimer's disease, the initial response contains a large amount of data from Semantic Scholar. The final summary also exhibits some inaccuracies. This example highlights some of the current limitations of the system.

**Workflow Step**	**Description**
User Input	*"Can we search scientific literature about new drugs for Alzheimer's disease?"*
Decisions	ACTION: Planning<br>ACTION: Research<br>REASON: User requests scientific literature on novel drugs for Alzheimer's disease.<br>SUB-TASK: Search literature on novel therapeutic drugs for Alzheimer's disease.<br>ACTION: Finalize<br>REASON: All requested literature has been retrieved and no further actions are required.<br>SUB-TASK: Produce final summary and next-step recommendations based on gathered results.
Tool Interaction	# / TOOL / INPUT / OUTPUT<br>1 / Semantic Scholar / novel therapeutic drug for Alzheimer's disease / 40 papers: EURO GEROSCIENCE Conference (2022); Advances in Gold Nanoparticles for the Diagnosis and Management of Alzheimer's Disease (2025); Commentary on Dartois et al.--model building in population PK-PD analyses. A 2002-2004 literature survey. (2007); +37 more<br>2 / Semantic Scholar / novel drug development Alzheimer disease 2023 / 23 papers: ACD856, a novel positive allosteric modulator of Trk receptors, single ascending doses in healthy subjects: Safety and pharmacokinetics (2024); Recent advances in Alzheimer's disease pathogenesis and therapeutics from an immune perspective (2023); Safety and effectiveness of the anti-amyloid monoclonal antibody (mAb) drug lecanemab for early Alzheimer's disease: The pharmacovigilance perspective (2025); +20 more
Response (truncated)	Research documenting promising Alzheimer's disease drug candidates from differing sources.

Literature Search on New Alzheimer's Drugs

Scientific literature on emerging therapeutics for Alzheimer's disease (AD) is abundant and actively evolving, as reflected in the curated research set. The provided collection documents progress across three complementary strategies: anti-amyloid monoclonal antibodies, small-molecule modulators targeting upstream pathogenic pathways, and advanced drug-delivery platforms that cross the blood–brain barrier [1].

Key Therapeutic Strategies and Representative Studies

- **Anti-amyloid monoclonal antibodies** – Real-world pharmacovigilance of lecanemab demonstrates slowing of cognitive decline while highlighting ARIA risk [1].
- **TRK receptor modulators** – ACD856, a novel positive allosteric modulator, achieved favorable pharmacokinetics and rapid brain penetration in healthy volunteers, indicating the feasibility of oral TRK targeting [2].
- **Small-molecule oligomer modulators** – Valiltramiprosate reduces Aβ oligomers and mitigates hippocampal atrophy, with added benefit in APOE4 carriers according to quantitative pharmacology modeling [3].
- **Drug repositioning** – Benzbromarone lowers AD incidence and Aβ expression, exemplifying a viable repurposing strategy supported by real-world data [4].
- **Multi-target directed ligands (MTDLs)** – Triazole-tethered benzothiazoles display potent dual AChE/β-amyloid inhibition and antioxidant activity, with compounds 10f and 10l showing superior preclinical efficacy [5].
- **Nanoparticle delivery systems** – Lipid nanocarriers (SLNs, NLCs, exosomes) facilitate efficient blood–brain barrier transcytosis and enhance Aβ clearance in preclinical models [6].
- **Inflammasome inhibition** – INF235 attenuates NLRP3 activation, brain iron deposits, and glial markers, improving cognition at the presymptomatic stage in 5×FAD mice [7].

Visualization	Sources can be displayed and directly accessed.<br><br>Safety and effectiveness of the anti-amyloid monoclonal antibody (mAb) drug lecanemab for early Alzheimer's disease: The pharmacovigilance perspective<br>British Journal of Clinical Pharmacology - 2025<br>Amy Bobbins, M. Davies, E. Lynn, Debabrata Roy, Alison Yeomans, S. Shakir<br><br>The development of humanized IgG1 anti-amyloid monoclonal antibodies, such as lecanemab, provides a promising novel treatment pathway with potential disease-modifying effects for patients with early Alzheimer's disease (AD). Lecanemab, which gained marketing approval by the United States Food and Drug Administration (US FDA) in July 2023, has since been approved in multiple countries, including the United Kingdom (UK). The decision by the UK's Medicines and Healthcare products Regulatory Agency (MHRA) to approve lecanemab in August 2024 followed similar regulatory decisions in the US and Japan. However, at the time of approval, the decision contrasted with that of the European Medicine Agency (EMA) in July 2024. Subsequently, the EMA recommended the marketing approval of lecanemab in November 2024 following a re-examination of further data submitted by the Marketing Authorisation Holder. The UK's National Institute for Health and Care Excellence (NICE) has not recommended lecanemab for use in early AD amid concerns, including treatment cost and the translation of efficacy outcomes into clinically meaningful improvement. The risks of serious adverse events (SAEs), including amyloid-related imaging abnormalities (ARIA), have also emerged from clinical trial data with a concern for the potential for rare, life-threatening events. This narrative review discusses the requirement for a robust method of monitoring the safety and effectiveness of lecanemab in the real-world clinical setting considering recent regulatory decisions. Additionally, the need to evaluate proposed risk minimization measures (RMMs) is discussed considering the resource constraints of healthcare systems, such as those faced by the UK's National Health Service (NHS).

An example ChatDRex network analysis workflow, including agent planning steps, NeDRex KG-based identification of Alzheimer's disease-related genes, DIAMOnD disease module detection, and corresponding user-facing interface snippets, is provided in Table S3 and is available online under the tab "Use Cases" identifiable by the respective user input (ID: 36b601b9-498b-4722-9dda-36990e97fc2a [3]).

Table S3: Network Analysis Workflow and User View in ChatDRex: This table outlines key agent workflow steps in ChatDRex for network-based analysis of Alzheimer's disease. First, the NeDRex KG tool retrieves 51 genes associated with Alzheimer's disease, ranked by their association score and presented as an annotated, clickable gene list with detailed node information. The user then runs DIAMOnD on six Alzheimer's disease-associated seed genes (CD2AP, ABI3, BACE1, ARC, TREM2, and MS4A4A), which expands them into a disease module of 106 nodes and 1,651 edges and identifies EGFR, SOS1, SH3BP1, and GRB2 as hubs connecting multiple seeds. The module is displayed in the interactive Drugst.One network view; the node and edge counts shown there (56 nodes, 606 edges)

reflect the currently displayed subnetwork rather than the full module. This example shows how ChatDRex combines KG retrieval and network-based module

**Workflow Step**	**Description**
**NeDRex KG tool: Finding Key Genes**	
User Input	*"Which genes are related to alzheimer?"*
Decisions	ACTION: Planning<br>ACTION: NeDRex KG<br>REASON: User requests gene list for Alzheimer disease; retrieve associations directly from the knowledge graph.<br>SUB-TASK: Retrieve genes associated with Alzheimer disease from the knowledge graph.<br>ACTION: Finalize<br>REASON: User query answered with gene list; no further requests.<br>SUB-TASK: Produce final summary and next-step recommendations based on gathered results.
Tools	# / TOOL / INPUT / OUTPUT<br>1 / NeDRex KG / Retrieve genes associated with Alzheimer disease from the knowledge graph. / 41+ records: APP, ABCA7, APOE, +38 more<br>2 / Drugst.One / Building Graph with 51 Entrez IDs / Network: 46 nodes, 72 edges: early-onset autosomal dominant Alzheimer disease, familial Alzheimer disease, Alzheimer disease, +43 more
Response (truncated)	Curated list of genes directly linked to Alzheimer's disease from NeDRex with corresponding Entrez IDs and database references.

Genes Related to Alzheimer's Disease

The NeDRex Knowledge Graph identifies **41 genes** that have been linked to Alzheimer's disease with varying levels of evidence strength. Each association score reflects the confidence in the gene–disease relationship, as determined by the graph's scoring algorithm. NeDRex

Ranked Gene List

Gene Symbol	Entrez ID	Score
APP	351	0.7817 NeDRex
ABCA7	10347	0.7067 NeDRex
APOE	348	0.6950 NeDRex
GRIN1	2902	0.6836 NeDRex
CDK5	1020	0.6796 NeDRex

Visualization

Annotated and clickable gene list for Alzheimer's disease with gene symbols, Entrez identifiers, and disease ontology references.

Gene Symbol	Entrez ID	Score
APP	351	0.7817 NeDRex
ABCA7	10347	0.7067 NeDRex
APOE	348	0.6950 NeDRex
GRIN1	2902	0.6836 NeDRex
CDK5	1020	0.6796 NeDRex
SORL1	6653	0.6776 NeDRex

Detail dialog with more information for individual node results.

<table>
<tr><td></td><td>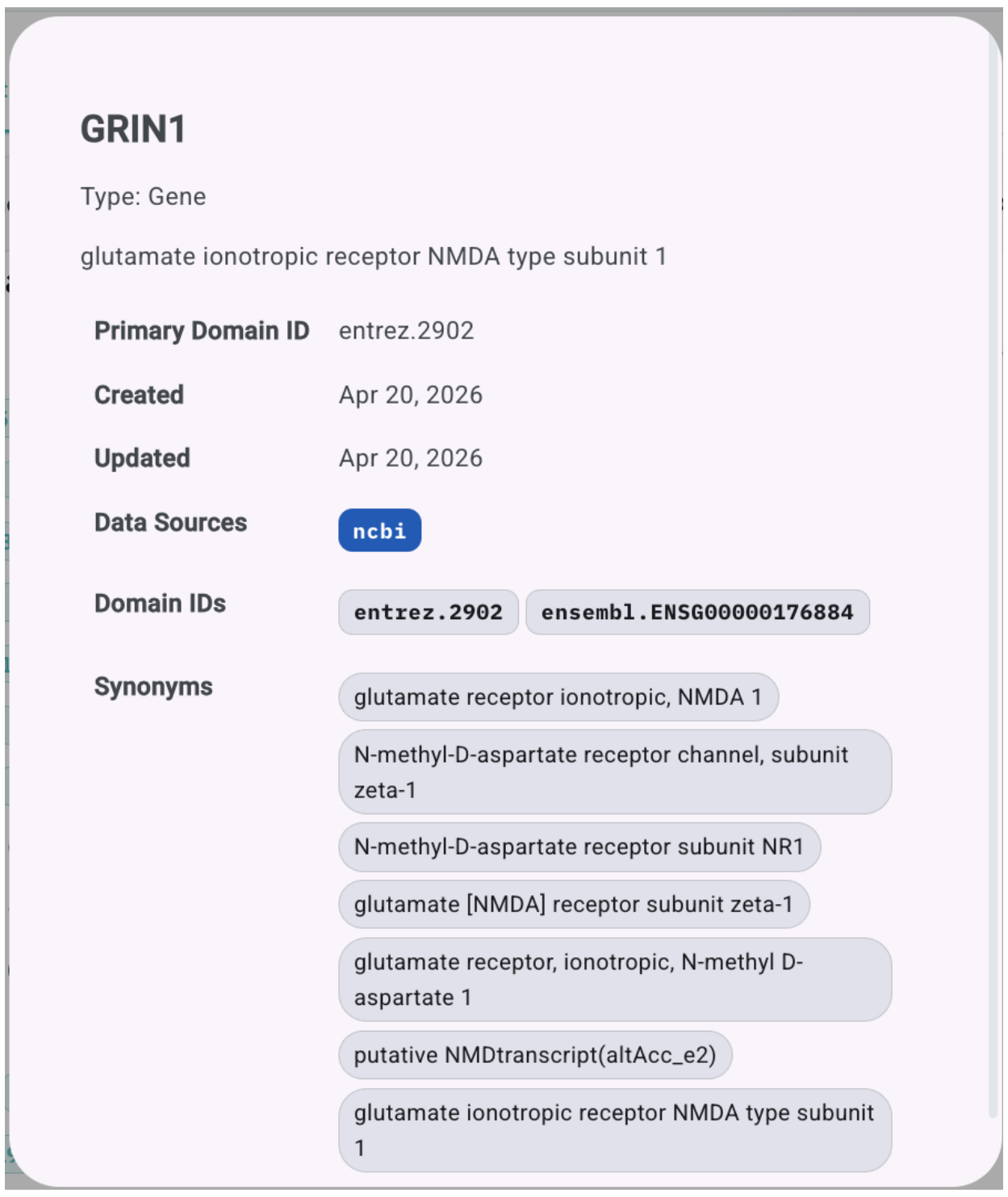
</td></tr>
<tr><td colspan="2">DIAMOnD tool: Identifying Disease Modules</td></tr>
<tr><td>User Input</td><td>“Run DIAMOnD for CD2AP, ABI3, BACE1, ARC, TREM2, MS4A4A”</td></tr>
<tr><td>Decisions</td><td>ACTION: Planning
ACTION: NeDRex
REASON: User requested DIAMOnD analysis on the seed genes CD2AP, ABI3, BACE1, ARC, TREM2, MS4A4A within the current network.
SUB-TASK: Run DIAMOnD on the genes of the current network module
ACTION: Finalize
REASON: User goal was only to run DIAMOnD; the requested analysis is complete.
SUB-TASK: Produce final summary and next-step recommendations based on gathered results.</td></tr>
</table>

Tools

#	TOOL	INPUT	OUTPUT
1	`Summary`	Your previous messages and the agent's responses	Run DIAMOnD for CD2AP, ABI3, BACE1, ARC, TREM2, MS4A4A; k not specified
2	`MyGene`	CD2AP, ABI3, BACE1, ARC, TREM2, MS4A4A	6 of 6 mapped `CD2AP → 23607 (CD2AP)` `ABI3 → 51225 (ABI3)` `BACE1 → 23621 (BACE1)` +3 more
3	`DIAMOnD`	DIAMOnD with 6 Entrez IDs	DIAMOnD · Network: 106 nodes, 1651 edges `ARC` `CD2AP` `BACE1` +103 more
4	`Drugst.One`	Analyzing Drugst.One network with question: NETWORK FACTS (authoritative — these counts are computed, not estimated; use them verbatim and do not...	Disease module identified by DIAMOnD	Item	Value	|------|-------|	Total number of nodes in the final module	106		Total number of edges in the final module	1 651		Seed-gene set (group "seednode")	6 genes – ARC, CD2AP, BACE1, ABI3, MS4A4A, TREM2		DIAMOnD–adde...

Response (truncated)

Network-based disease module detection showing DIAMOnD execution on six Alzheimer's disease-related seed genes.

DIAMOnD-Added Gene Set

DIAMOnD expanded the module by adding **100** non-seed genes (the "DIAMOnD - Node" group).
These added nodes introduce extensive connectivity among the seeds, forming a densely connected subnetwork.

- Total added nodes with at least one seed edge: 29 `Network`
- Added nodes lacking any direct seed edge: 71 `Network`

Cross-Seed Hubs

Four non-seed genes receive connections from two or more seed genes, serving as integrative hubs within the module:

Hub	Seeds it connects to
EGFR	ARC, CD2AP
SOS1	CD2AP, ABI3
SH3BP1	CD2AP, ABI3
GRB2	CD2AP, ABI3

`Network`

These multi-seed connections suggest that EGFR, SOS1, SH3BP1, and GRB2 may coordinate signalling pathways arising from the disease–associated seed genes.

Module Size

The final DIAMOnD module comprises **106 nodes** (6 seeds + 100 added genes) and contains **1 651 edges**, reflecting a highly interconnected network structure. `Network`

Visualization

Interactive network visualization interface displaying disease modules with seed genes and integrated analysis tools for pathway and drug

	exploration. 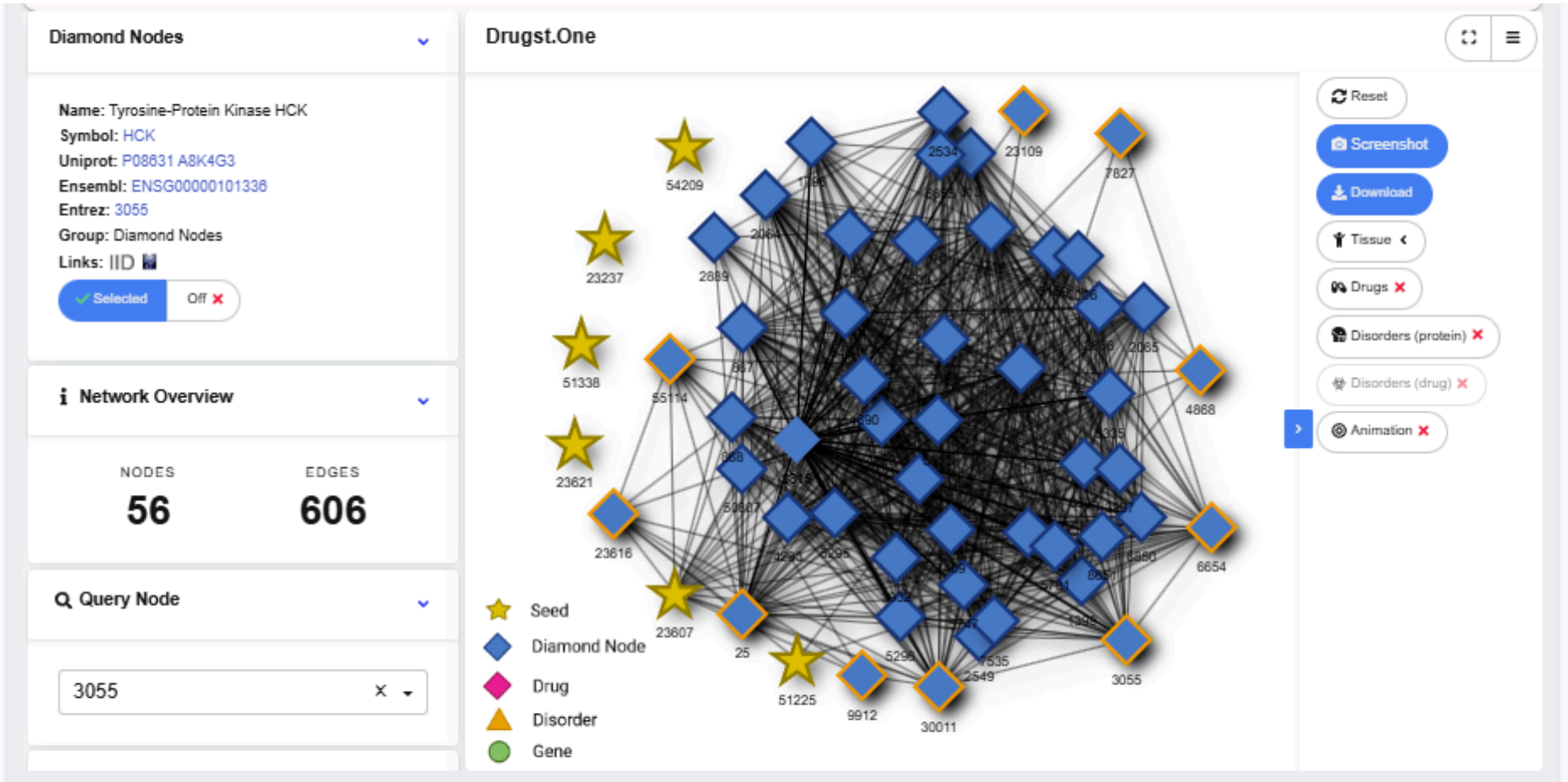

An example ChatDRex workflow for colorectal cancer, including NeDRex KG-based retrieval of disease-associated genes, DIAMOnD-based disease module expansion, DIGEST-supported functional coherence evaluation, drug–target identification, and KG- and literature-based verification, is provided in Table S4 and is available online under the tab "Use Cases", identifiable by the respective user input(ID: 8164ed88-d42b-4647-9090-4937dd027e80 [4]).

Table S4: Disease Module Detection Workflow and User Interaction in ChatDRex: This table summarizes the key workflow steps of a colorectal cancer use case. ChatDRex retrieves 38 canonical colorectal cancer genes (including APC, TP53, KRAS, SMAD4, PIK3CA, BRAF, MLH1 and MSH2) from the NeDRex KG. It then expands them with DIAMOnD into a disease module of 138 nodes and 2,129 edges (38 seeds, 100 added genes). The tool trace lists 39 Entrez IDs as DIAMOnD input because GREM1 is passed twice; the duplicate collapses into a single node, yielding the 38 unique seed genes reported for the module. DIGEST evaluates the functional coherence of the top 10 module genes and reports empirical P values of 0.000999 for GO.CC and KEGG and 0.002 for GO.BP. The most frequent terms relate to cellular compartments and to KEGG pathways in cancer, Ras signalling and PI3K-Akt signalling. ChatDRex finally attempts to verify interactions among the top 10 genes. It finds no direct interactions in the NeDRex KG and only partial support in the literature. The example shows how ChatDRex supports interactive network medicine analyses, while also highlighting current limitations such as sparse KG coverage of pairwise interactions and the limited specificity of broad cellular component terms.

**Agent Workflow Step**	**Description**
**NeDRex KG tool: Finding Key Genes**	
User Input	*“Show me the colorectal cancer disease module and its key genes/pathways.”*

Decisions	ACTION: Planning<br><br>ACTION: NeDRex KG<br>REASON: Need to obtain the colorectal cancer disease module genes and their relations first.<br>SUB-TASK: What are the genes associated with colorectal cancer in NeDRex KG?<br><br>ACTION: DIGEST<br>REASON: User requested key pathways for colorectal cancer module; perform functional coherence analysis.<br>SUB-TASK: Run DIGEST on the genes of the colorectal cancer disease module to identify enriched pathways.<br><br>ACTION: Finalize<br>REASON: DIGEST analysis completed; all requested information is available.<br>SUB-TASK: Produce final summary and next-step recommendations based on gathered results.

Tools

TOOLS

#	TOOL	INPUT	OUTPUT
1	NeDRex KG	Retrieve all genes linked to colorectal cancer from NeDRex KG.	38+ records<br>MLH1 MSH6 APC +35 more
2	Drugst.One	Building Graph with 49 Entrez IDs	Network: 42 nodes, 83 edges<br>GREM1 colorectal adenocarcinoma colorectal cancer +39 more
3	MyGene ×3	–	No identifiers provided.
4	MyGene	MLH1, 4292, MSH6, 2956, APC, 324, TP53, 7157, POLD1, 5424, MSH2, 4436, BRAF, 673, CTNNB1, 1499, POLE, 5426, AXIN2, 8313, FGFR3, 2261, MLH3, 27030, SMAD7, 4092,...	78 of 78 mapped<br>MLH1 → 4292 (MLH1) 4292 → 4292 (MLH1) MSH6 → 2956 (MSH6) +75 more
5	DIAMOnD	DIAMOnD with 39 Entrez IDs	DIAMOnD · Network: 138 nodes, 2129 edges<br>TMEM238L CHEK2 POU2AF3 +135 more
6	Drugst.One	Analyzing Drugst.One network with question: NETWORK FACTS (authoritative — these counts are computed, not estimated; use them verbatim and do not...	Disease module obtained by DIAMOnD on colorectal-cancer seed genes | Item | Count | |------ |-------| | Total nodes | 138 | | Total edges | 2,129 | | Seed genes (group "seednode") | 38 | | DIAMOnD-added genes (group "diamondnode") | 100 | | Non-seed nodes with ≥1 seed neighbour |...

Response (truncated)

The colorectal-cancer disease module is defined by the NeDRex KG seed genes expanded with the DIAMOnD algorithm, resulting in a network that captures both canonical drivers and their immediate functional neighbours.

Module Overview

The module contains 138 nodes and 2,129 edges, comprising 38 seed genes (group "seednode") and 100 DIAMOnD-added genes (group "DIAMOnD - Node"). Network

Seed Genes

The seed core consists of the canonical colorectal-cancer drivers such as **APC, TP53, KRAS, SMAD4, PIK3CA, BRAF, MLH1, MSH2, MSH6, MSH3, MLH3, BRCA2, CHEK2, ATM, BRCA1, SMAD7, AXIN2, CTNNB1, CTNNA1, ZMIZ1, PREX1, EIF3H, FLCN, EP300, GREM1, SLCO2A1, GREM1, BLM, TERT, TCF7L2, BUB1B, MYC, POU5F1B, FGF6, LATS2, BRIP1**. These genes are listed

<table>
<tr><td>Visualization</td><td>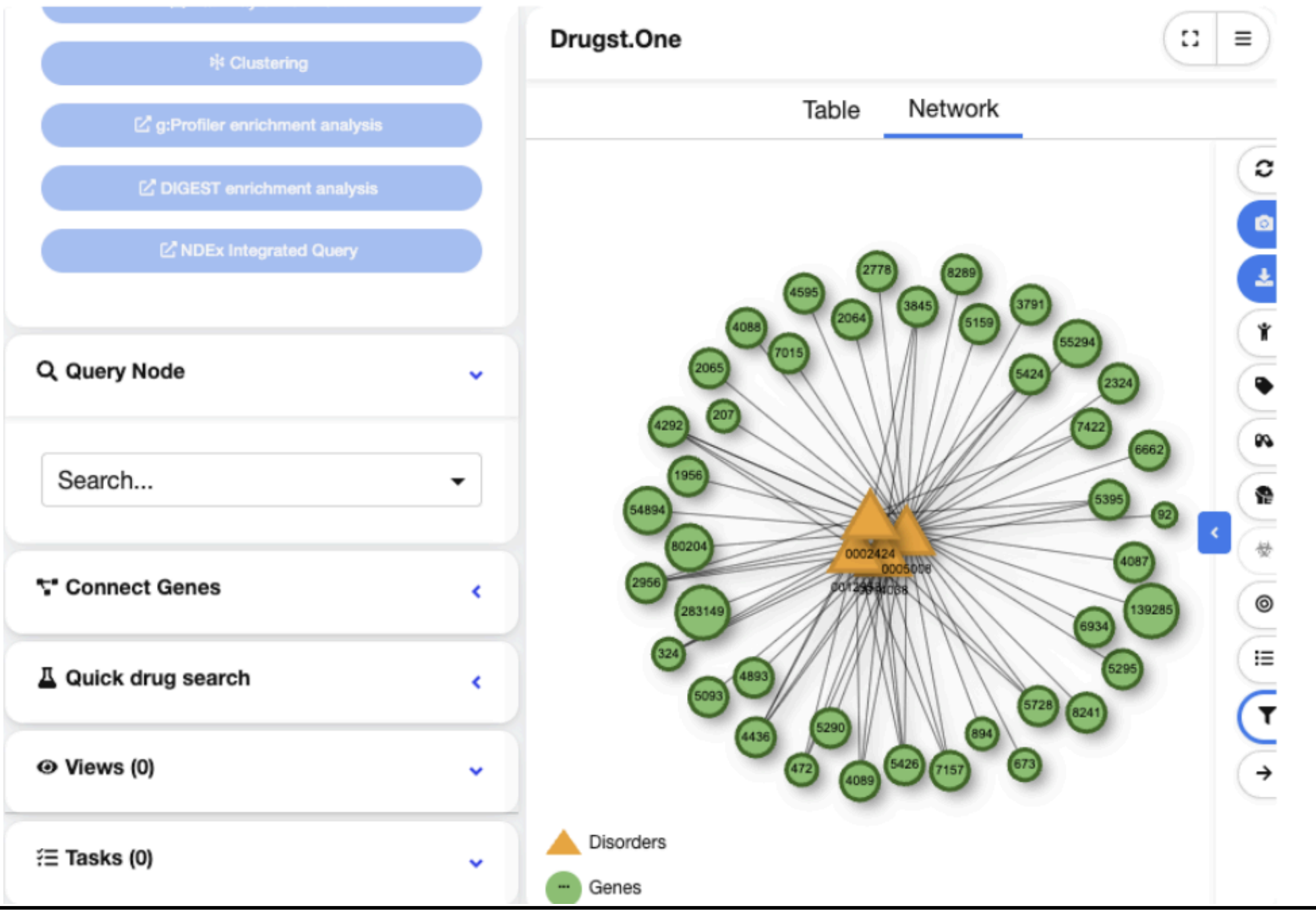
</td></tr>
<tr><td colspan="2">DIAMOnD tool: Identifying Disease Modules</td></tr>
<tr><td>User Input</td><td>“run DIAMOnD with this 5426 (POLE), 5424 (POLD1), 121364 (OR10A7), 93492 (TPTE2), 92922 (CCDC102A), 6299 (SALL1), 5782 (PTPN12), 4952 (OCRL), 4605 (MYBL2), 4430 (MYO1B), 4343 (MOV10), 4248 (MGAT3), 84808 (PERM1), 84700 (MYO18B), 84643 (KIF2B), 84256 (FLYWCH1), 83872 (HMCN1), 80336 (PABPC1L), 80230 (RUFY1)”</td></tr>
<tr><td>Decisions</td><td>Decisions
ACTION: Planning
ACTION: Finalize
REASON: DIAMOnD already executed; no further user request.
SUB-TASK: Produce final summary and next-step recommendations based on gathered results.</td></tr>
<tr><td>Tools</td><td>DECISIONS
ACTION: Planning
ACTION: Finalize
REASON: DIAMOnD already executed; no further user request.
SUB-TASK: Produce final summary and next-step recommendations based on gathered results.

TOOLS
<table>
<tr><th>#</th><th>TOOL</th><th>INPUT</th><th>OUTPUT</th></tr>
<tr><td>1</td><td>Summary</td><td>Your previous messages and the agent's responses</td><td>User requests DIAMOnD with seeds: APC, TP53, KRAS, SMAD4, PIK3CA, BRAF, MLH1, MSH2, MSH6, MSH3, MLH3, BRCA2, CHEK2, ATM, BRCA1, SMAD7, AXIN2, CTNNB1, CTNNA1, ZMIZ1 ... (+16 more); Prior: DIAMOnD already run on this seed set producing 138-node module with 100 added genes; module de...</td></tr>
</table></td></tr>
<tr><td>Response (truncated)</td><td>Disease module composition with annotated seed genes (Entrez IDs and gene symbols) from the DIAMOnD algorithm output. The displayed user input lists only a subset of the relevant genes. ChatDRex correctly resolved the request using the conversation history and identified the matching</td></tr>
</table>

	previously generated DIAMOnD result, and returned the corresponding module information. The response therefore reflects the relevant prior DIAMOnD run rather than only the genes explicitly repeated in the displayed input.

Connectivity Between Seeds and Added Genes

- 99 of the 100 added nodes have at least one direct edge to a seed node, while 1 added node has no seed neighbor. `Network`
- 95 added nodes are connected to two or more seed nodes, indicating higher integration into the seed network. `Network`
- Example seed–neighbor relationships (selected):
- CHEK2 is directly linked to LATS2, BRCA1, E2F1, MPRIP, MDM4, ATM, MSH2, XRCC5, TP53, PRKDC, AKT1, RB1, FBXW7, BRCA2, WEE1, FZR1, RAD9A, CDKN1A, PTPN11, BLM, ATR, CHEK1, ERBB2, PTEN, SPOP, PPM1D, CDC6, CDK4, FOXO3, RFC1, VHL, NBN. `Network`
  - CTNNA1 is connected to ZNF510, EPHA2, SMAD1, MAP2K3, MYC, TCF7L2, AKT1, SMAD4, BRAF, E2F3, MET, PTPN11, EGFR, ERBB2, PTEN, SPOP, BRCA1, CTNNB1, CD44, APC. `Network`
  - CTNNB1 is linked to XRCC5, PTPN11, PRKDC, CDK6, AXIN2, SMAD4, FBXW7, NF2, ERBB2, GLIS2, FZR1, FANCF, ARNT, HIF1A, CDK2, FANCA, MET, EGFR, HSP90AA1, CCNA2, LATS2, UHRF2, PCNA, PTEN, SMARCA4, CDK1, BRCA1, MYC, MPRIP, FANCE, TCF7L2, TERT, FOXO3, APC, TP53, VHL, EP300, AKT1, CCND1, FANCC, EPHA2, CCNE1, KAT2A, E2F3, MAP2K3, CTNNA1, ZMIZ1, SMAD7, PIK3CA, FANCG. `Network`
  - Additional seed–neighbor patterns are summarized for all 38 seed nodes in the provided adjacency lists. `Network`

Summary of Module Size and Connectivity

Feature	Value	Source
Total nodes	138	Network
Total edges	2129	Network
Seed nodes	38	Network
Added nodes	100	Network
Added nodes with ≥1 seed edge	99	Network
Added nodes with ≥2 seed edges	95	Network

DIGEST: Pathway and Process Enrichment Assessment

User Input	*“Take the top 10 genes and run a pathway and GO term enrichment analysis on this gene set.”*
Decisions	**Decisions**<br>ACTION: `Planning`<br>ACTION: `DIGEST`<br>REASON: User requests GO enrichment of the top 10 DIAMOnD genes; digest analysis directly provides functional coherence and enriched terms.<br>SUB-TASK: Run DIGEST on the genes AKT1, SMARCA4, FBXW7, SMAD1, FANCC, PTPN11, FGF23, SMAD4, FGF6, PTEN for GO enrichment.<br>ACTION: `Finalize`<br>REASON: DIGEST analysis completed; coherence and frequent terms are available, satisfying the user request.<br>SUB-TASK: Produce final summary and next-step recommendations based on gathered results.

Tools

#	TOOL	INPUT	OUTPUT
1	**Summary**	Your previous messages and the agent's responses	User requests GO pathway coherence evaluation for the top 10 DIAMOnD genes: AKT1, SMARCA4, FBXW7, SMAD1, FANCC, PTPN11, FGF23, SMAD4, FGF6, PTEN; also wants the most enriched GO terms. Prior: DIAMOnD analysis already identified these top 10 genes as the most connected additions...
2	**MyGene**	AKT1, SMARCA4, FBXW7, SMAD1, FANCC, PTPN11, FGF23, SMAD4, FGF6, PTEN	10 of 10 mapped<br>`AKT1 → 207 (AKT1)`<br>`SMARCA4 → 6597 (SMARCA4)`<br>`FBXW7 → 55294 (FBXW7)` +7 more
3	**DIGEST-Set**	207, 6597, 55294, 4086, 2176, 5781, 8074, 4089, 2251, 5728	Coherent in 4 of 4 databases<br>`GO.CC (p = 1.0e-3)` `KEGG (p = 1.0e-3)`<br>`GO.BP (p = 0.002)` +1 more

Response (truncated)

Evaluation of the top 10 DIAMOnD genes for GO-based pathway coherence The functional coherence of the 10 genes (AKT1, SMARCA4, FBXW7, SMAD1, FANCC, PTPN11, FGF23, SMAD4, FGF6, PTEN) was assessed by the DIGEST coherence validation, which reports empirical P-values, mean pairwise Jaccard indices, and mappability for several annotation databases.
All reported P-values are below 0.05, indicating significant coherence in each database.

Gene Ontology Cellular Component (GO.CC)

- Empirical P-value: 0.000999 – significant coherence `DIGEST`.
- Coherence score (mean pairwise Jaccard index): 0.199 `DIGEST`.
- Mappability: 10/10 genes annotated (all 10 genes) `DIGEST`.
- Most frequent terms:
  - cytoplasm (GO:0005737) – 9 genes `DIGEST`.
  - nucleus (GO:0005634) – 8 genes `DIGEST`.
  - nucleoplasm (GO:0005654) – 8 genes `DIGEST`.
  - cytosol (GO:0005829) – 7 genes `DIGEST`.
  - chromatin (GO:0000785) – 4 genes `DIGEST`.

KEGG Pathways

- Empirical P-value: 0.000999 – significant coherence `DIGEST`.
- Coherence score: 0.055 `DIGEST`.
- Mappability: 10/10 genes annotated (all 10 genes) `DIGEST`.
- Most frequent terms:
  - Pathways in cancer (hsa05200) – 5 genes `DIGEST`.
  - Ras signaling pathway (hsa04014) – 4 genes `DIGEST`.
  - PI3K-Akt signaling pathway (hsa04151) – 4 genes `DIGEST`.

Visualization	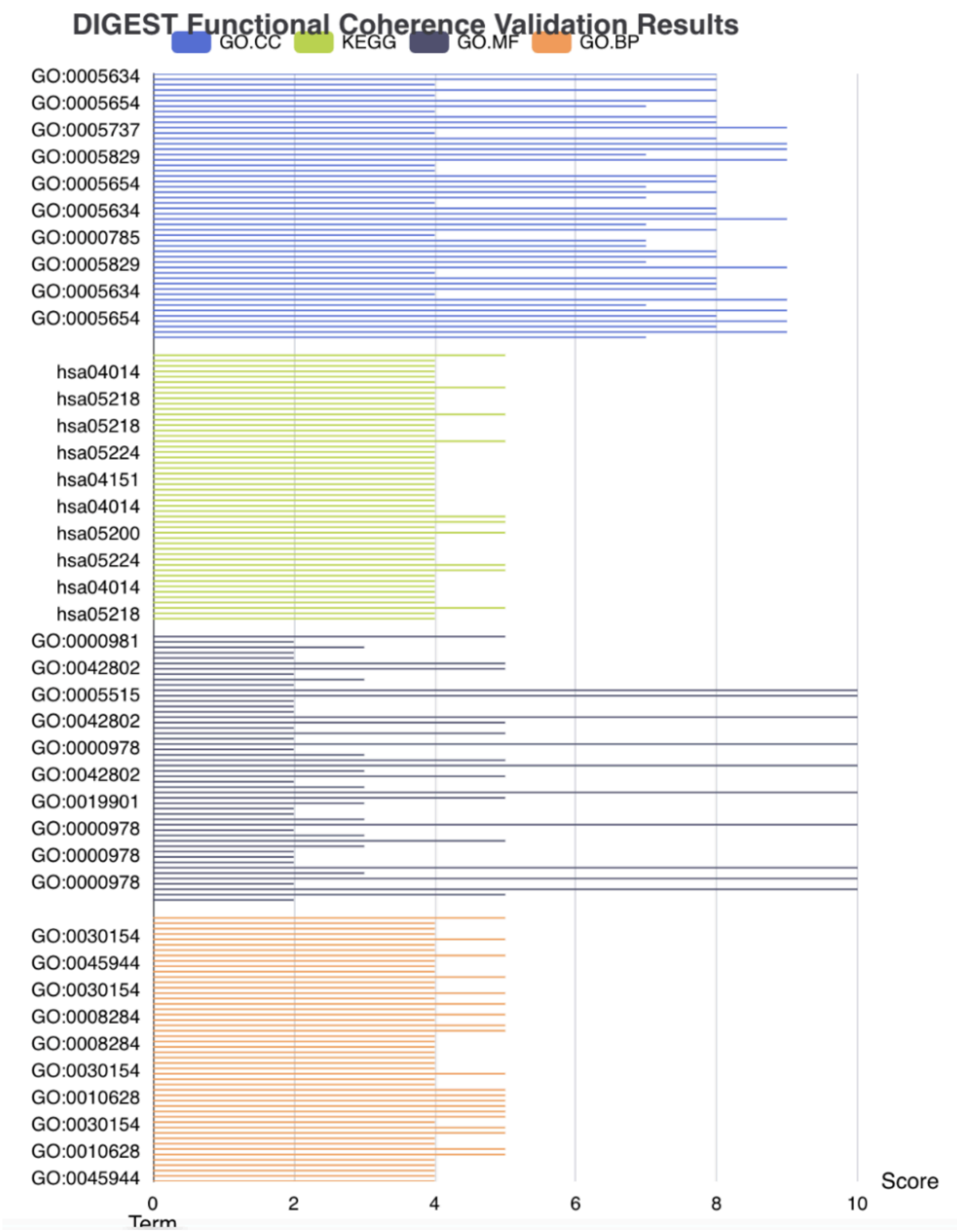<br>All DIGEST outputs can also be viewed via the 'Files' tab
User Input	*"Can you find drugs that work on 5426"*

Decisions	ACTION: **Planning**<br><br>ACTION: **NeDRex KG**<br>REASON: Need KG evidence for interactions among the top 10 DIAMOnD genes.<br>SUB-TASK: Retrieve NeDRex KG interactions among AKT1, SMARCA4, FBXW7, SMAD1, FANCC, PTPN11, FGF23, SMAD4, FGF6, PTEN.<br><br>ACTION: **Research**<br>REASON: KG lookup returned no interactions; need literature evidence for the 10 DIAMOnD genes.<br>SUB-TASK: Search literature for pairwise interactions among AKT1, SMARCA4, FBXW7, SMAD1, FANCC, PTPN11, FGF23, SMAD4, FGF6, PTEN.<br><br>ACTION: **Finalize**<br>REASON: All requested evidence has been gathered; no further actions needed.<br>SUB-TASK: Produce final summary and next-step recommendations based on gathered results.
Tool	(see table below)

#	TOOL	INPUT	OUTPUT
1	**Summary**	Your previous messages and the agent's responses	The NeDRex KG list provided does not include the top 10 DIAMOnD genes (AKT1, SMARCA4, FBXW7, SMAD1, FANCC, PTPN11, FGF23, SMAD4, FGF6, PTEN) or their direct interactions, so the connection cannot be verified within the KG as presented. No research evidence for these specific pai...
2	**NeDRex KG**	Retrieve NeDRex KG interactions among AKT1, SMARCA4, FBXW7, SMAD1, FANCC, PTPN11, FGF23, SMAD4, FGF6, PTEN.	No relevant nodes were provided for this question.
3	**Semantic Scholar**	AKT1 SMARCA4 interaction	5 papers<br>Characteristics of miRNA interaction with mRNA of candidate genes of the non-small cell lung cancer (2018)<br>AKT1, LKB1, and YAP1 Revealed as MYC Interactors with NanoLuc-Based Protein-Fragment Complementation Assay (2017)<br>Identification and Validation of SNP-Containing Genes With Prognostic Value in Gastric Cancer via Integrated Bioinformatics Analysis (2021)<br>+2 more
4	**Semantic Scholar**	AKT1 PTEN interaction	40 papers<br>Type 2 Diabetes Mellitus Mediation by the Disruptive Activity of Environmental Toxicants on Sex Hormone Receptors: In Silico Evaluation (2021)<br>Downregulated miR-23b-3p expression acts as a predictor of hepatocellular carcinoma progression: A study based on public data and RT-qPCR verification (2018)<br>Ginsenosides as promising hepatoprotective natural drugs: progress, challenges and opportunities (2026)<br>+37 more

Response (truncated)	**Verification in the literature**<br>The research summary states that evidence for pairwise links among these genes is sparse. The following mechanistic interactions are supported by the literature:<br>• PTEN directly binds and dephosphorylates phosphorylated Akt1, a key step in PI3K/Akt signalling. [1] [2]<br>• PTEN also associates with FANCC in a neuro-protective pathway that modulates mitophagy after spinal-cord injury. [3]<br>• SMAD1 and SMAD4 collaborate in BMP/TGF-β signalling, forming a transcriptionally active complex. [4]<br>• SMAD1 physically interacts with PLEKHO2, a regulator of TGF-β/Smad signalling. [5] No direct literature links are available for SMARCA4, FBXW7, PTPN11, FGF23, or FGF6 with any of the other nine genes in the current evidence base. Research<br>**Conclusion**<br>• In the NeDRex KG, the top-10 DIAMOnD genes are not present in the raw KG list, but the network summary does include several of them and their pairwise edges, indicating partial verifiability.
Visualization	
User Input	*"Is this connection also verifiable in NeDrex KG and via research?"*
Decisions	ACTION: Planning<br>ACTION: Research<br>REASON: Need literature evidence for drug–target connections shown in the KG (Gemcitabine and Cytarabine targeting POLE)<br>SUB-TASK: Search PubMed for studies linking Gemcitabine or Cytarabine to inhibition of POLE<br>ACTION: NeDRex KG<br>REASON: Need to confirm if NeDRex KG includes the POLE–Gemcitabine/Cytarabine interaction.<br>SUB-TASK: Retrieve any NeDRex KG records linking POLE with Gemcitabine or Cytarabine.<br>ACTION: Finalize<br>REASON: Both literature search and KG query completed; no further actions needed.<br>SUB-TASK: Produce final summary and next-step recommendations based on gathered results.

Tools

#	TOOL	INPUT	OUTPUT
1	**Summary**	Your previous messages and the agent's responses	User asks if this connection is verifiable in NeDrex KG and via research; No explicit connection stated; Prior discussion indicates EGFR is linked to colorectal adenocarcinoma in NeDRex (score 0.6757) and literature supports EGFR's role in CRC; AURKA connects to multiple seed ge...
2	**Semantic Scholar**	Gemcitabine POLE inhibition	4 papers<br>Restoring hemidesmosomes to prevent cancer cell invasiveness (2013)<br>DNA Repair gene alterations and efficacy from gemcitabine and nab-paclitaxel with/without durvalumab and tremelimumab in metastatic pancreatic ductal adenocarcinoma (2026)<br>Abstract 293: Screening the druggable genome for synthetic lethal interactions with the CHK1 inhibitor PNT737 (2017)<br>+1 more
3	**Semantic Scholar**	gemcitabine POLE	25 papers<br>552 Rétinopathie à gemcitabine chez un patient diabétique (2009)<br>Mitotic checkpoints, MUGs and kinetochores (2013)<br>Appréciation du risque d'exposition aux médicaments anticancéreux à l'aide des prélèvements surfaciques (2018)<br>+22 more
4	**Semantic Scholar**	gemcitabine inhibits POLE	4 papers<br>Mitotic checkpoints, MUGs and kinetochores (2013)<br>54Therapeutic Targeting of IDHl Mutations in Intrahepatic Cholangiocarcinoma Arising in Oilier Disease (2026)<br>Restoring hemidesmosomes to prevent cancer cell invasiveness (2013)<br>+1 more

Response (truncated)

NeDRex KG Evidence

- **POLE (Entrez 5426)** is linked to **Gemcitabine (DrugBank DB00441)** in the NeDRex KG `NeDRex`.
- **POLE (Entrez 5426)** is also linked to **Cytarabine (DrugBank DB00987)** in the NeDRex KG `NeDRex`. These associations are the only gene-drug links present for POLE in the provided KG data `NeDRex`.

Literature Evidence

The research list contains four PubMed/Semantic Scholar articles that do not address POLE inhibition by Gemcitabine or Cytarabine:

- 7c1df0275c099e47bbd8010d649efa5184753b08 – discusses pancreatic cancer biology but not POLE [1].
- 80921b005f38f9fd4791a616130d0559168879a4 – reports POLE mutations in tumors, without mentioning drug inhibition [2].
- 08e58b1de6898aaeff4c7b98b9dfc9e925e695f5 – describes a gemcitabine-related retinopathy case, with no POLE link [3].
- 08ed56bd124ef72cd4ee3e4d9ac5d66c91dcb5af – covers mitotic checkpoints and gemcitabine's effect on mitosis, not POLE inhibition [4]. No study in the provided research set demonstrates that Gemcitabine or Cytarabine directly inhibits POLE activity. Thus, while the NeDRex KG records a drug–gene association, there is no supporting literature evidence within the supplied data [1] [2] [3] [4].

Visualization	Each source can also be opened in detail with a single click.<br>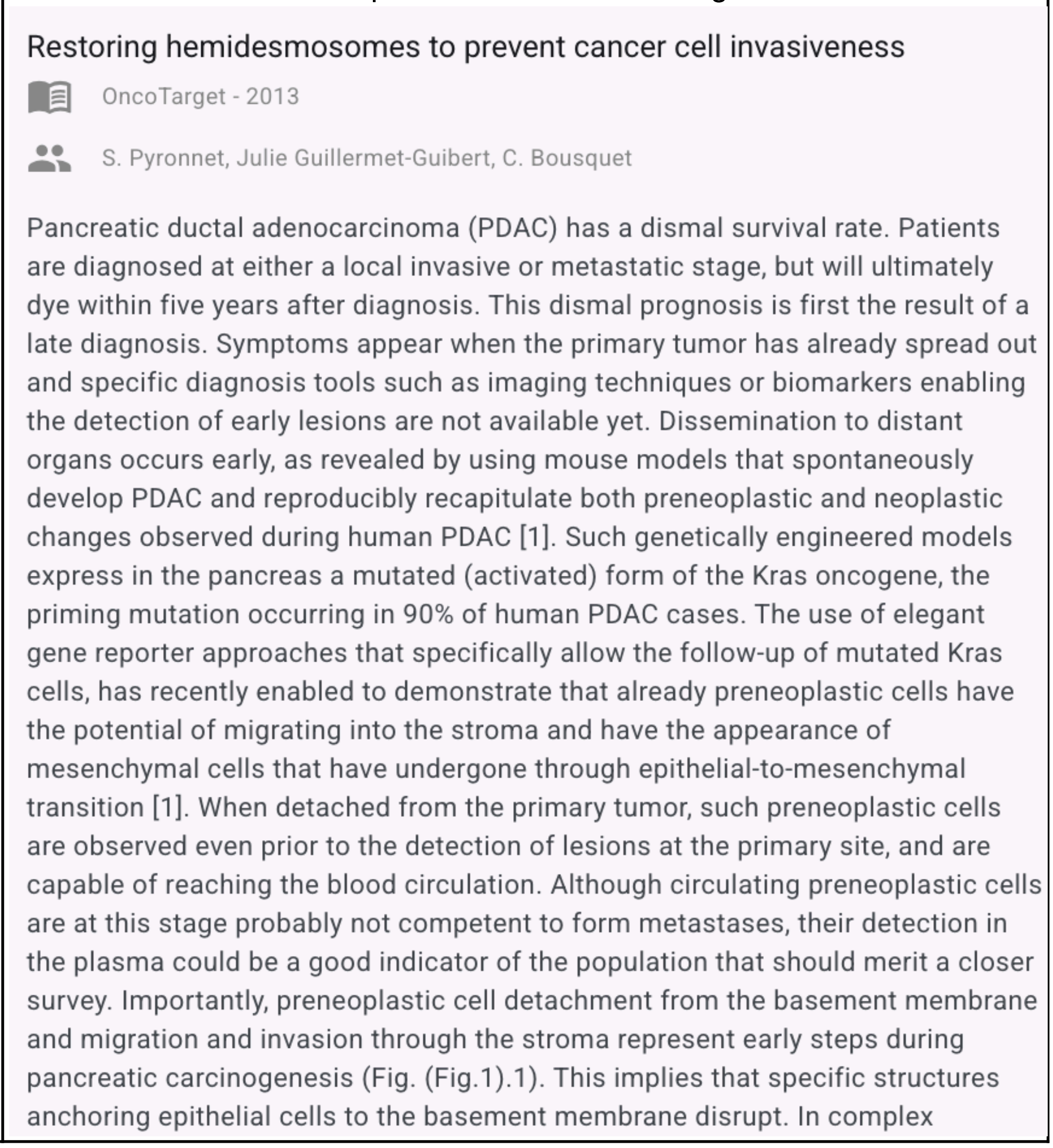<br>Restoring hemidesmosomes to prevent cancer cell invasiveness<br>OncoTarget - 2013<br>S. Pyronnet, Julie Guillermet-Guibert, C. Bousquet<br>Pancreatic ductal adenocarcinoma (PDAC) has a dismal survival rate. Patients are diagnosed at either a local invasive or metastatic stage, but will ultimately dye within five years after diagnosis. This dismal prognosis is first the result of a late diagnosis. Symptoms appear when the primary tumor has already spread out and specific diagnosis tools such as imaging techniques or biomarkers enabling the detection of early lesions are not available yet. Dissemination to distant organs occurs early, as revealed by using mouse models that spontaneously develop PDAC and reproducibly recapitulate both preneoplastic and neoplastic changes observed during human PDAC [1]. Such genetically engineered models express in the pancreas a mutated (activated) form of the Kras oncogene, the priming mutation occurring in 90% of human PDAC cases. The use of elegant gene reporter approaches that specifically allow the follow-up of mutated Kras cells, has recently enabled to demonstrate that already preneoplastic cells have the potential of migrating into the stroma and have the appearance of mesenchymal cells that have undergone through epithelial-to-mesenchymal transition [1]. When detached from the primary tumor, such preneoplastic cells are observed even prior to the detection of lesions at the primary site, and are capable of reaching the blood circulation. Although circulating preneoplastic cells are at this stage probably not competent to form metastases, their detection in the plasma could be a good indicator of the population that should merit a closer survey. Importantly, preneoplastic cell detachment from the basement membrane and migration and invasion through the stroma represent early steps during pancreatic carcinogenesis (Fig. (Fig.1).1). This implies that specific structures anchoring epithelial cells to the basement membrane disrupt. In complex

An exemplary ChatDRex module-detecting workflow for Huntington's disease includes user-driven disease specification, NeDRex retrieval of Huntington's disease-associated genes using the NeDRex KG, disease module expansion using DIAMOnD, functional characterization using DIGEST, TrustRank-based drug candidate prioritization, and corresponding user ineraction steps, is provided in Table S5 and is available online under the tab "Use Cases", tab identifiable by the respective user inputs (IDs: 08889d39-8eec-4512-a38a-048a20562f04 [5]).

Table S5: Huntington's Disease Module Detection and Drug Prioritization Workflow in ChatDRex: This table summarizes the key workflow steps that ChatDRex executes for a Huntington's disease use case. The workflow begins when the user requests genes associated with Huntington's disease. ChatDRex then retrieves these genes, as well as other disease-associated genes, from the NeDRex KG, including HTT, DRD3, DRD2, SLC18A2, and SLC2A3. These genes are then used as seed genes for DIAMOnD-based disease module detection. The resulting module is then evaluated using DIGEST to determine functional coherence and biological pathway relevance. Finally, TrustRank identifies drugs with interactions to proteins within the identified disease module. Note that "seed" refers to two different sets in this workflow: the five disease-associated genes (HTT, DRD3, DRD2, SLC18A2, and SLC2A3) are the DIAMOnD seeds, whereas the "14 seed proteins" in the drug-ranking response are TrustRank seeds, that is, the module proteins

directly targeted by the ten top-ranked drugs. This approach prioritizes drugs targeting module components through network pharmacology rather than claiming direct disease efficacy. This workflow illustrates how ChatDRex integrates knowledge graph retrieval, network-based disease module detection, functional interpretation, and drug prioritization within a conversational framework.

Example: Huntington's disease

User Input

"Which genes are associated with Huntington's Disease"

Decisions

ACTION:	Planning
ACTION:	NeDRex KG
REASON:	User wants list of genes associated with Huntington's disease; KG provides curated gene-disease links.
SUB-TASK:	Retrieve all genes linked to Huntington's disease from NeDRex KG.
ACTION:	Finalize
REASON:	All requested genes linked to Huntington's disease have been retrieved.
SUB-TASK:	Produce final summary and next-step recommendations based on gathered results.

Tools

#	TOOL	INPUT	OUTPUT
1	NeDRex KG	Retrieve all genes linked to Huntington's disease from NeDRex KG.	5 records<br>HTT SLC18A2 DRD2 +2 more
2	Drugst.One	Building Graph with 15 Entrez IDs	Network: 8 nodes, 7 edges<br>Huntington disease<br>Huntington disease and related disorders<br>Huntington disease-like syndrome +5 more

Response (truncated)

Gene-disease associations retrieved from NeDRex showing Huntington's disease-linked genes with standardized identifiers.

Which genes are associated with Huntington's disease?

The NeDRex knowledge graph (KG) identifies five genes that it links to Huntington's disease, providing an association score for each gene. These genes and their Entrez identifiers are listed below along with the KG-derived scores.

Gene	Entrez ID	Score
HTT NeDRex	3064 NeDRex	0.705 NeDRex
SLC18A2 NeDRex	6571 NeDRex	0.584 NeDRex
DRD2 NeDRex	1813 NeDRex	0.393 NeDRex
SLC2A3 NeDRex	6515 NeDRex	0.389 NeDRex
DRD3 NeDRex	1814 NeDRex	0.330 NeDRex

These entries represent all genes that the NeDRex KG currently links to Huntington's disease.

Visualization	Interactive network visualization interface displaying disease modules with seed genes and integrated analysis tools for pathway and drug exploration.<br>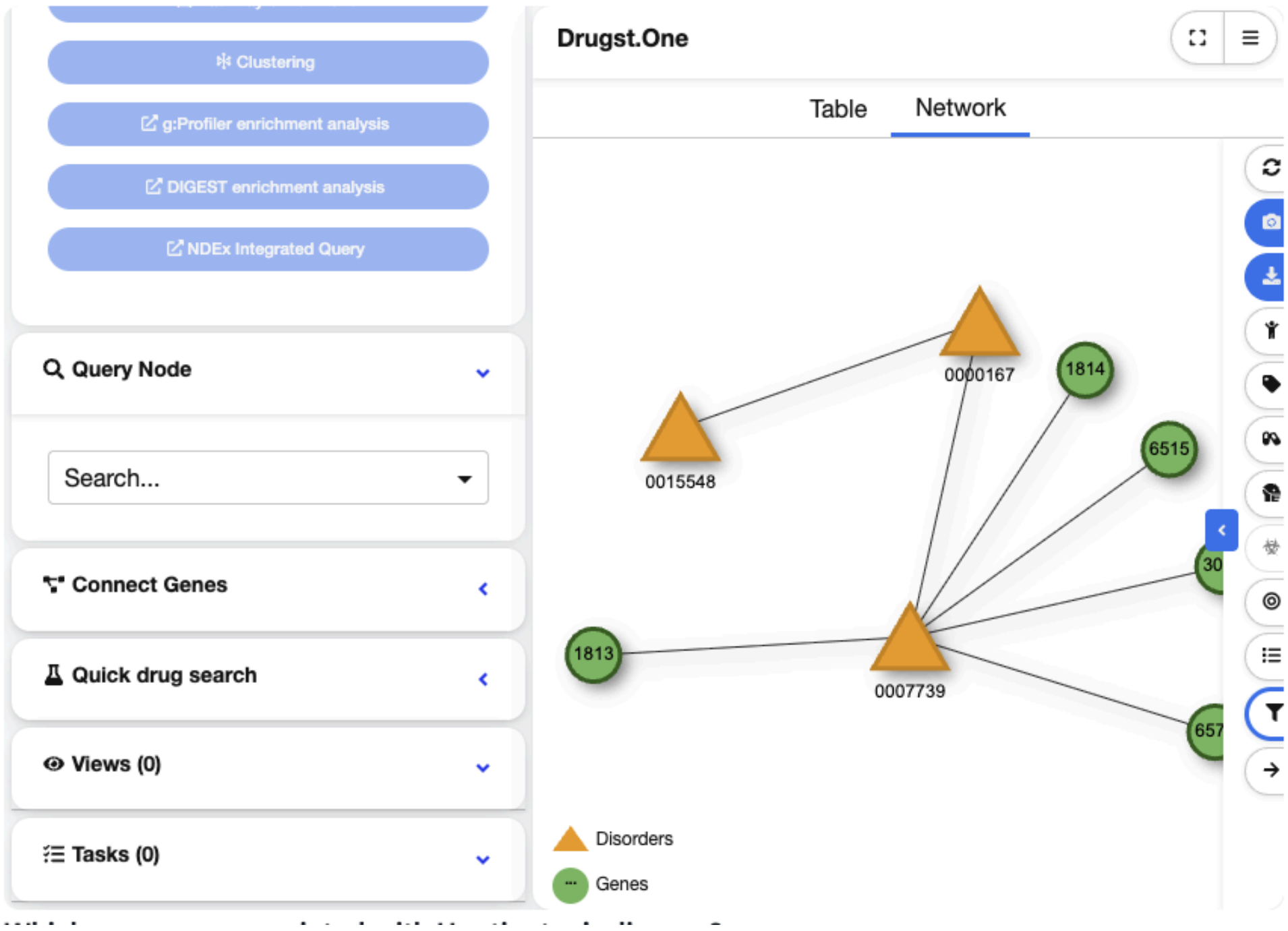
User Input	*"Let's identify the disease modules from those genes"*
Decisions	ACTION: Planning<br>ACTION: NeDRex<br>REASON: Expand the disease module by adding functionally related genes around the seed genes.<br>SUB-TASK: Run DIAMOnD on the genes HTT, SLC18A2, DRD2, SLC2A3, DRD3<br>ACTION: NeDRex<br>REASON: Identify drugs that target or could be repurposed for the disease module.<br>SUB-TASK: Run TrustRank on the genes of the current network module<br>ACTION: Finalize<br>REASON: Module identification, functional validation, and drug ranking are complete.<br>SUB-TASK: Produce final summary and next-step recommendations based on gathered results.

Tools

#	TOOL	INPUT	OUTPUT
1	`Summary`	Your previous messages and the agent's responses	User requests to identify disease modules from the genes HTT, SLC18A2, DRD2, SLC2A3, DRD3; prior discussion listed these five genes as associated with Huntington's disease per NeDRex KG.
2	`MyGene` ×2	HTT, SLC18A2, DRD2, SLC2A3, DRD3	5 of 5 mapped `HTT → 3064 (HTT)` `SLC18A2 → 6571 (SLC18A2)` `DRD2 → 1813 (DRD2)` `+2 more`
3	`DIAMOnD`	DIAMOnD with 5 Entrez IDs	DIAMOnD · Network: 105 nodes, 650 edges `DRD2` `DRD3` `HTT` `+102 more`
4	`Drugst.One`	Analyzing Drugst.One network with question: NETWORK FACTS (authoritative — these counts are computed, not estimated; use them verbatim and do not...	Disease module created by the DIAMOnD run | Item | Detail | |------|--------| | Seed genes (group “seednode”) | HTT, SLC18A2, DRD2, SLC2A3, DRD3 | | Added genes (group “diamondnode”) | 100 genes added by DIAMOnD – the top-ranked list is: GPR143, EPB41L1, EPB41, CLIC6, PPRC1,...

Response (truncated)

Detailed breakdown of the Huntington’s disease module showing which seed genes are present.

Disease Module Overview

The disease module inferred from the five seed genes (DRD2, DRD3, HTT, SLC2A3, SLC18A2) comprises 105 genes connected by 650 protein-protein interaction edges. The network is split into a **seed-node group** of five genes and a **DIAMOnD-node group** of 100 additional genes added by the DIAMOnD algorithm `Network`.

Seed Genes

- DRD2
- DRD3
- HTT
- SLC2A3
- SLC18A2 These are the only seed nodes that initiated the module construction `Network`.

DIAMOnD Nodes

The DIAMOnD run introduced 100 DIAMOnD-node genes, which together with the seeds form the complete module. Examples of DIAMOnD nodes include GPR143, EPB41L1, EPB41, CLIC6, PPRC1, ADORA2A, HRH3, etc., but the full lis is provided in the network summary `Network`.

Direct Seed Neighbourhoods

User Input

“Let's run a functional analysis over that module”

Decisions

ACTION: `Planning`

ACTION: `DIGEST`
REASON: User requested functional analysis (enrichment) of the module.
SUB-TASK: Run DIGEST on the genes of the current network module

ACTION: `Finalize`
REASON: Functional coherence validation completed; no further actions needed.
SUB-TASK: Produce final summary and next-step recommendations based on gathered results.

<table>
<tr><td>Tools</td><td>

#	TOOL	INPUT	OUTPUT
1	**Summary**	Your previous messages and the agent's responses	User requests functional analysis of the module comprising 5 seed genes (HTT, SLC18A2, DRD2, SLC2A3, DRD3) plus 100 added genes; module has 105 nodes and 650 edges. Prior steps identified this composition via DIAMOnD; no additional parameters provided.
2	**MyGene**	HTT, SLC18A2, DRD2, SLC2A3, DRD3	5 of 5 mapped `HTT → 3064 (HTT)` `SLC18A2 → 6571 (SLC18A2)` `DRD2 → 1813 (DRD2)` +2 more
3	**DIGEST-Subnetwork**	1813, 1814, 3064, 6515, 6571, 4935, 2036, 2035, 54102, 23082, 135, 11255, 1268, 2770, 3356, 1815, 3765, 3760, 3763, 3762, 154, 2550, 2782, 2775, 54331, 150,...	Coherent in 3 of 4 databases `GO.BP (p = 1.0e-3)` `GO.CC (p = 1.0e-3)` `KEGG (p = 1.0e-3)` +1 more

</td></tr>
<tr><td>Response (truncated)</td><td>

Subnetwork-based coherence analysis reveals significant GO annotations associated with the Huntington's disease module.

Functional Coherence of the Module

The module contains 105 genes and 650 interactions, as determined by DIAMOnD (seed = 5; added = 100). Network

Gene Ontology Biological Process

- Empirical P-value: 0.000999 – significant coherence.
- Coherence score (mean pairwise Jaccard index): 0.031.
- Mappability: 104 of 105 genes annotated.
- Most frequent annotations:
  - G protein-coupled receptor signaling pathway (GO:0007186) – 51 genes.
  - Signal transduction (GO:0007165) – 18 genes.
  - Regulation of G protein-coupled receptor signaling pathway (GO:0008277) – 14 genes.
  - Adenylate cyclase-activating G protein-coupled receptor signaling pathway (GO:0007189) – 13 genes.
  - Adenylate cyclase-modulating G protein-coupled receptor signaling pathway (GO:0007188) – 12 genes. DIGEST

Gene Ontology Cellular Component

</td></tr>
</table>

Visualization	Ranked enrichment analysis showing relative significance of biological pathways and GO terms.<br>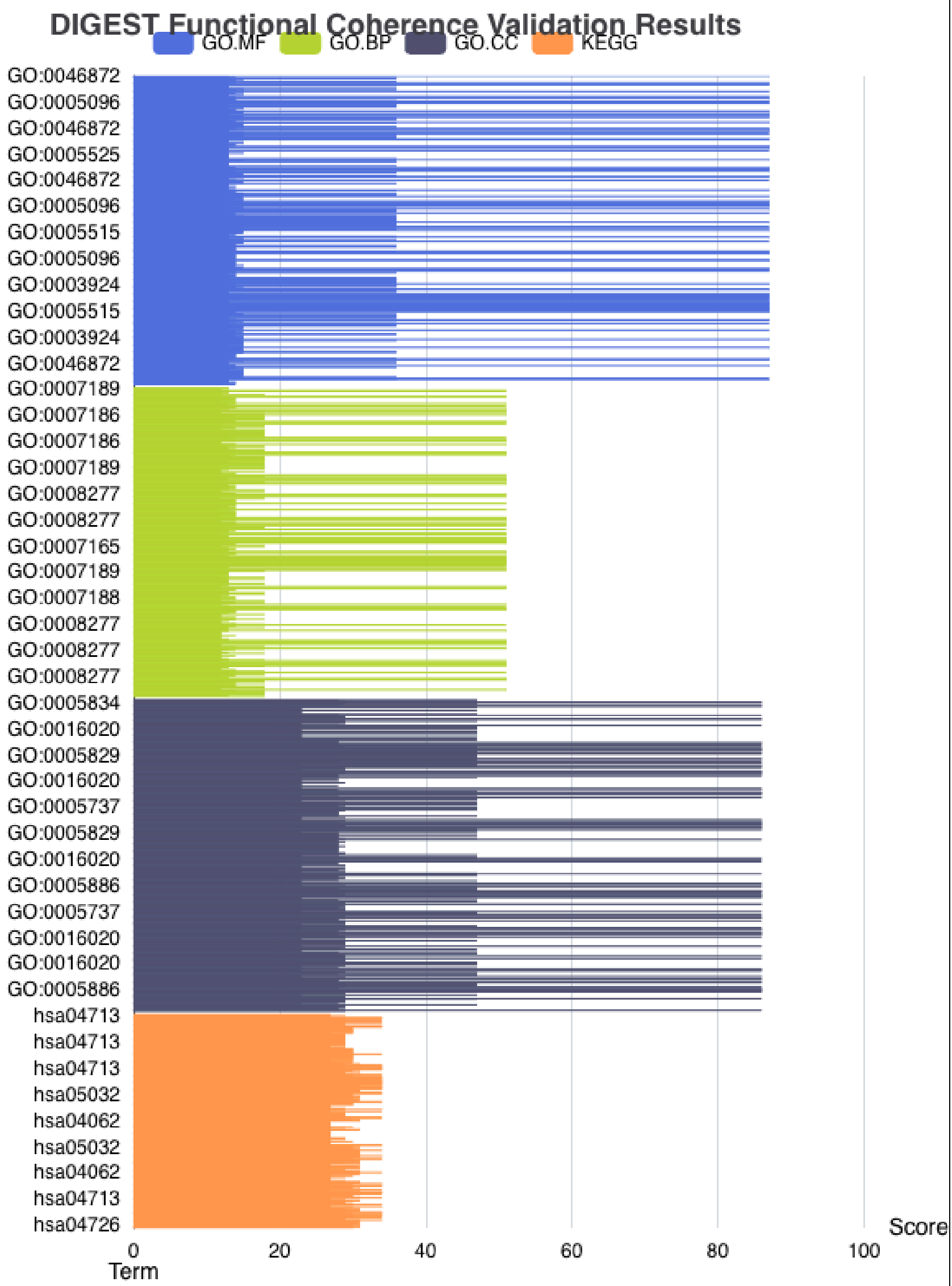
User Input	*"Which existing drug can target that module?"*
Decisions	ACTION: Planning<br>ACTION: NeDRex<br>REASON: User requests existing drug(s) targeting the disease module; TrustRank ranks drugs by relevance to module genes.<br>SUB-TASK: Run TrustRank on the genes of the current network module to rank drugs<br>ACTION: Finalize<br>REASON: TrustRank ranking of drugs against module already completed; no further actions needed.<br>SUB-TASK: Produce final summary and next-step recommendations based on gathered results.

Tools

#	TOOL	INPUT	OUTPUT
1	`Summary`	Your previous messages and the agent's responses	User asks for existing drug targeting the module; Prior: module derived via DIAMOnD contains seed genes HTT, SLC18A2, DRD2, SLC2A3, DRD3 plus 100 added nodes; No drug information has been provided in previous steps; uncertain which drugs target this module.
2	`MyGene`	HTT, SLC18A2, DRD2, SLC2A3, DRD3	5 of 5 mapped<br>`HTT → 3064 (HTT)`<br>`SLC18A2 → 6571 (SLC18A2)`<br>`DRD2 → 1813 (DRD2)` `+2 more`
3	`UniProt`	3064, 6571, 1813, 6515, 1814	Entrez:3064 Hit:P42858 (Entrez:3064) Entrez:6571 Hit:Q05940 (Entrez:6571) Entrez:1813 Hit:P14416 (Entrez:1813) Entrez:6515 Hit:P11169 (Entrez:6515) Entrez:1814 Hit:P35462 (Entrez:1814)
4	`TrustRank`	TrustRank with 105 UniProt IDs	TrustRank · Network: 24 nodes, 95 edges<br>`Chlorpromazine` `Clozapine` `Miconazole`<br>`+21 more`
5	`Drugst.One`	Analyzing Drugst.One network with question: NETWORK FACTS (authoritative — these counts are computed, not estimated; use them verbatim and do not...	Top ranked drugs in the TrustRank module	Rank	Drug (Label – ID)	Targeted seed proteins (label – id)	|------|-------------------|----------------------------------|	1	Chlorpromazine – DB00477	ADRA2A (P08913), DRD2 (P14416), HTR2A (P28223), DRD3 (P35462), HRH4 (Q9...

Response (truncated)

Transparent interpretation of findings with acknowledgment of current limitations and recommendations for extended analysis.

Existing drugs that target the module

The network summary lists a group of ten "founddrug" nodes that directly interact with the 14 seed proteins of the module. These drugs and their corresponding targets are:

Drug (DrugBank ID)	Targeted seed proteins
Chlorpromazine – [DB00477]	ADRA2A, DRD2, HTR2A, DRD3, HRH4, DRD4, DRD5, CHRM1, OPRD1
Clozapine – [DB00363]	ADRB1, ADRA2A, DRD2, HTR2A, DRD3, HRH4, DRD4, ADRB2, DRD5, CHRM1
Miconazole – [DB01110]	ADRB1, ADRA2A, DRD2, ADORA2A, CNR1, HTR2A, DRD3, ADORA1, DRD4, CHRM1, OPRD1
Econazole – [DB01127]	ADRB1, ADRA2A, DRD2, ADORA2A, CNR1, HTR2A, DRD3, ADORA1, DRD4, CHRM1, OPRD1
Zotepine – [DB09225]	ADRB1, ADRA2A, DRD2, HTR2A, DRD3, HRH4, DRD4, ADRB2, DRD5, CHRM1
Thioridazine – [DB00679]	ADRA2A, DRD2, HTR2A, DRD3, HRH4, DRD4, DRD5, CHRM1
Astemizole – [DB00637]	ADRB1, ADRA2A, DRD2, HTR2A, DRD3, HRH4,

Visualization	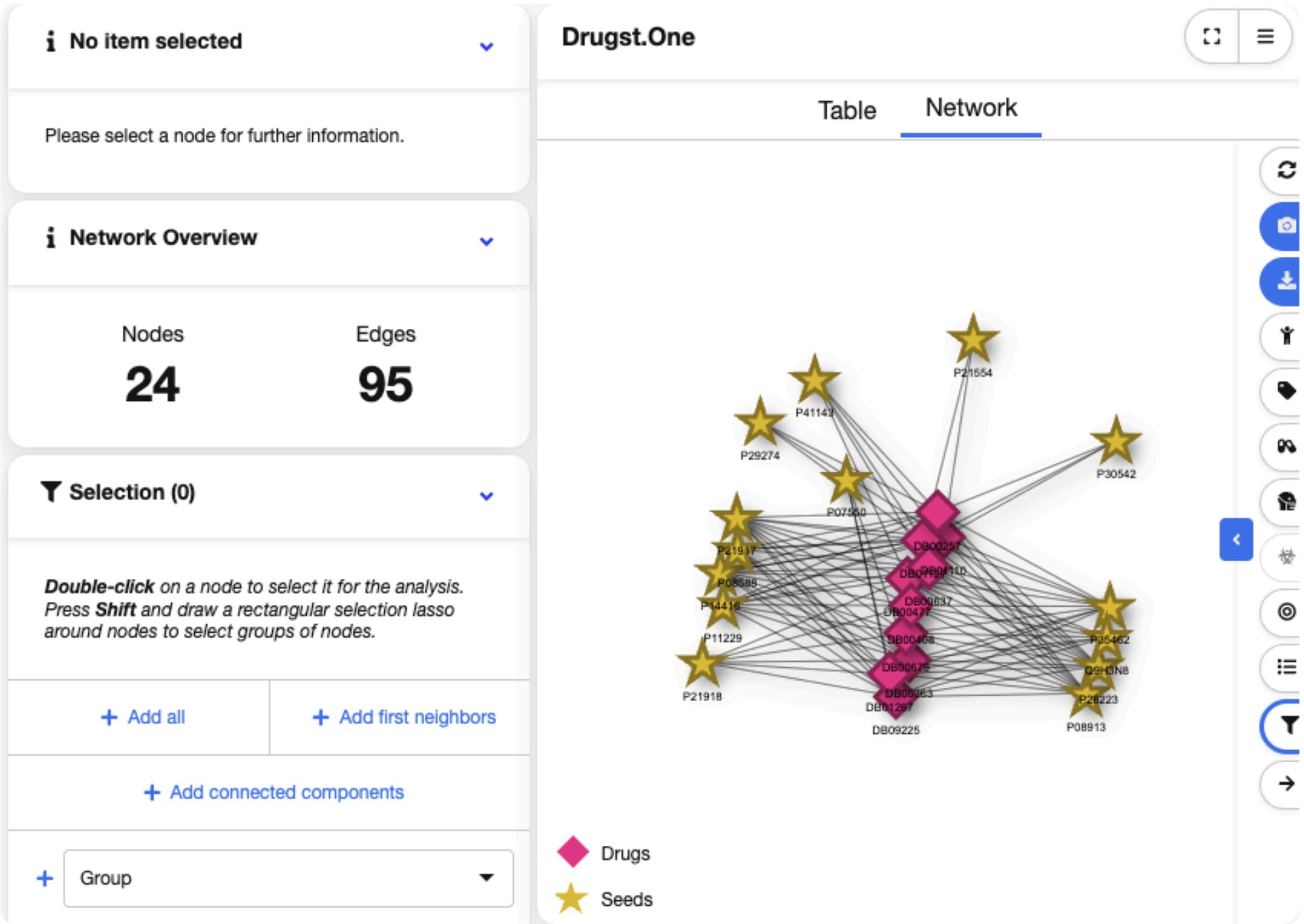
User Input	*"Find research on these drugs"*
Decisions	ACTION: Planning<br><br>ACTION: Research<br>REASON: User wants literature on the ten identified drugs for potential repurposing.<br>SUB-TASK: Search literature for research on Chlorpromazine, Clozapine, Miconazole, Econazole, Zotepine, Thioridazine, Astemizole, Loxapine, Paliperidone, Clotrimazole related to drug repurposing.<br><br>ACTION: Finalize<br>REASON: All requested research literature has been retrieved; no further actions remain.<br>SUB-TASK: Produce final summary and next-step recommendations based on gathered results.

Tools

#	TOOL	INPUT	OUTPUT
1	Summary	Your previous messages and the agent's responses	User requests research on these drugs; prior output identified ten existing drugs targeting the module: Chlorpromazine [DB00477], Clozapine [DB00363], Miconazole [DB01110], Econazole [DB01127], Zotepine [DB09225], Thioridazine [DB00679], Astemizole [DB00637], Loxapine [DB00408],...
2	Semantic Scholar	Chlorpromazine drug repurposing	40 papers<br>Inhibition of the replication of SARS-CoV-2 in human cells by the FDA-approved drug chlorpromazine (2020)<br>Network-driven prioritization and functional phenotyping nominate TTC23 as a biomarker-informed target in chlorpromazine repurposing for glioblastoma (2026)<br>Neuroimmune pleiotropy links COVID-19 outcomes to brain structural and functional imaging-derived phenotypes (2025)<br>+37 more
3	Semantic Scholar ×2	Clozapine drug repurposing	23 papers<br>From Therapeutic Drug to Xenobiotic in Cancer Repurposing: Clozapine Mechanisms, Metabolic Liabilities, and Human-Relevant Translational Approaches (2026)<br>Next-generation precision medicine for suicidality prevention (2024)<br>The cellular mechanism of antipsychotic-induced myocarditis: A systematic review. (2023)<br>+20 more

Response (truncated)

The literature review identified eleven peer-reviewed studies that report on repurposing activities for the ten drugs linked to the module (Chlorpromazine, Clozapine, Miconazole, Econazole, Zotepine, Thioridazine, Astemizole, Loxapine, Paliperidone, Clotrimazole). The most extensive evidence concerns clozapine and miconazole; the remaining agents have only sparse or mechanistic reports.

Clozapine

- **Oncology** – shows activity against glioblastoma, breast cancer, and melanoma brain metastases at concentrations that require dose optimization [1] [2].
- **Multiple Sclerosis** – reduces pro-inflammatory cytokines in patient monocytes, suggesting disease-modifying potential [3].

Miconazole

- **Oncology** – induces G0/G1 arrest and apoptosis via p53 in prostate cancer cell lines; reproducible across independent assays [4] [5].
- **Antiviral** – down-regulates thioredoxin-like 1 to suppress West Nile virus replication in neuronal cultures [6].
- **Malaria** – sustained-release nanostructured lipid carriers reduce parasitaemia in mouse models, supporting a systemic antimalarial strategy [7].
- **Nanomedicine** – olive oil–based nanoemulsion improves solubility and cytotoxicity against breast, liver, and colorectal cancer cells; docking suggests aromatase-like binding [8].
- **Target Discovery** – lipid-based chemical proteomics identified SGPL1 inhibition, linking miconazole to anti-inflammatory pathways in multiple sclerosis models [9] and predicted synergy with albendazole for Alzheimer's disease [10].

Visualization

The source is displayed as soon as you hover over it.

Miconazole

- **Oncology** – induces G0/G1 arrest and apoptosis via p53 in prostate cancer cell lines; reproducible across independent assays [4] [5].
- **Antiviral** – down-regulates thioredoxin-like 1 to suppress West Nile virus replication in neuronal cultures [6].
- **Malaria** – sustained-release nanostructured lipid carriers reduce parasitaemia in mouse models, supporting a systemic antimalarial strategy [7].
- **Nanomedicine** – olive oil–based nanoemulsion improves solubility and cytotoxicity against breast, liver, and colorectal cancer cells; docking suggests aromatase-like binding [8]

SOURCE 2026

Enhancing anticancer efficacy of miconazole via olive oil-based nanoemulsion: a molecularly-driven approach to drug repurposing

H. F. Alotaibi, Mustafa A. Al-Qadhi

Open Chemistry

matory

Clicking on it shows the source in detail.

Enhancing anticancer efficacy of miconazole via olive oil-based nanoemulsion: a molecularly-driven approach to drug repurposing

Open Chemistry - 2026

H. F. Alotaibi, Mustafa A. Al-Qadhi

Abstract Miconazole, a well-known antifungal drug, has recently gained attention for its potential anticancer properties, prompting efforts to enhance its therapeutic performance through innovative delivery systems. This study explores the repurposing of miconazole for anticancer applications using a green nanoemulsion delivery system. Olive oil was selected as a natural, biocompatible carrier to improve miconazole's bioavailability and therapeutic targeting. The formulated miconazole-loaded nanoemulsion exhibited an average particle size of 177.76 ± 5.2 nm and a zeta potential of −24 ± 0.94 mV, indicating physical stability. The formulation showed potent cytotoxicity against MCF-7 breast cancer cells (IC50 = 1.4 ± 0.4 µg/ml), with apoptosis induction of 70.31 ± 2.1 %. Broader anticancer activity was observed against HePG2 (IC50 = 10.7 ± 1.2 µg/ml) and HCT-116 (IC50 = 5.9 ± 0.7 µg/ml). Molecular docking revealed favorable binding of miconazole to target proteins, resembling interactions of the aromatase inhibitor letrozole, thereby supporting its anticancer potential. The findings support the use of olive oil-based nanoemulsions as an effective platform for repurposing miconazole in cancer therapy. This delivery approach enhances drug solubility, cellular uptake, and anticancer efficacy, offering a promising strategy for targeted and cost-effective treatment development.

Generated research questions are displayed under the "Sources" tab.

SEARCH QUERY `"Chlorpromazine drug repurposing"`

SOURCE 2020

Inhibition of the replication of SARS-CoV-2 in human cells by the FDA-approved drug chlorpromazine

M. Plaze

Publication — Open

SOURCE 2026

Network-driven prioritization and functional phenotyping nominate TTC23 as a biomarker-informed target in chlorpromazine repurposing for glioblastoma

Jian-Qi Hao, Hong-Bin Liu, Yongqiang Ye, Jianwei Lv, Fang Xue, Yanli Cai

Frontiers in Pharmacology — Open

SOURCE 2025

Neuroimmune pleiotropy links COVID-19 outcomes to brain structural and functional imaging-derived phenotypes

Qian-Yu Chen, Jun He, Brenda Cabrera-Mendoza, Ziang Xu, D. Qiu, R. Polimanti

medRxiv — Open

Multimedia Appendix 4 References

1. ChatDrex. Available from: https://apps.cosy.bio/chatdrex/use-cases/ed459d5f-29ad-46a1-a4dd-da4c67781378 [accessed Sept 23, 2026]
2. ChatDrex. Available from: https://apps.cosy.bio/chatdrex/use-cases/18194734-7bd2-4d9e-abb7-f976c174b5a6 [accessed Sept 23, 2026]
3. ChatDrex. Available from: https://apps.cosy.bio/chatdrex/use-cases/36b601b9-498b-4722-9dda-36990e97fc2a [accessed Sept 23, 2026]
4. ChatDrex. Available from: https://apps.cosy.bio/chatdrex/use-cases/8164ed88-d42b-4647-9090-4937dd027e80 [accessed Sept 23, 2026]
5. ChatDrex. Available from: https://apps.cosy.bio/chatdrex/use-cases/08889d39-8eec-4512-a38a-048a20562f04 [accessed Sept 23, 2026]
