# Supplementary material for: Conversational Multi-Agentic Network-Driven Disease Module Identification and Drug Repurposing Prediction With ChatDRex: Platform Development Study
Source: JMIR AI. 2026 Nov 3;5:e93862. doi: 10.2196/93862 (PMC13730514; doi:10.2196/93862)
Supplement: Supplementary file 5 [file ai-v5-e93862-s005.pdf]

Multimedia Appendix 5. ChatDRex Usability Feedback Form

Professional background:
☐ Clinician
☐ Biologist
☐ Bioinformatician
☐ Other: ____________

Programming experience:
☐ None
☐ Basic
☐ Intermediate
☐ Advanced

Experience with biomedical databases or network tools:
☐ None
☐ Basic
☐ Intermediate
☐ Advanced

Task Feedback

Please complete the assigned ChatDRex task and answer the following questions.

Task Completion

Were you able to complete the task?

☐ Yes, without help
☐ Yes, with minor help
☐ Yes, with major help
☐ No

Did any errors or misunderstandings occur?

☐ No
☐ Yes, please describe:

User Experience

Please rate the following statements from 1 to 5.

1 = strongly disagree, 5 = strongly agree

Statement	1	2	3	4	5

I understood how to interact with ChatDRex.	☐	☐	☐	☐	☐
I could complete the task without programming knowledge.	☐	☐	☐	☐	☐
The system responses were understandable.	☐	☐	☐	☐	☐
The biomedical results were presented clearly.	☐	☐	☐	☐	☐
I trusted the system’s explanation of the result.	☐	☐	☐	☐	☐
I would use a system like ChatDRex for similar tasks.	☐	☐	☐	☐	☐

Open Feedback

What worked well?

What was confusing or difficult?

Which part of the system should be improved?

Do you think ChatDRex supports a no-code workflow for users without programming expertise? Why or why not?

To evaluate ChatDRex's user-centered usability, we conducted a pilot study with a group of seven bioinformaticians and one clinician expert. The participants evaluated the system across several dimensions, including task completion success, system interaction, trust, and overall usability (Table S1). Quantitative feedback indicates strong system performance. Participants reported high levels of trust in the system's predictions and successfully completed assigned tasks with minimal to no external assistance.

Table S1: Overview of User Feedback Collected During the Usability Pilot Study: This table summarizes participant demographics (professional background, programming and biomedical experience), task completion success, and quantitative ratings regarding system interaction, trust, and usability (Q1–Q6). A dash indicates that no response was provided by

the participant. “Bioinf.” denotes “Bioinformatician,” “Clinic.” denotes “Clinician,” “Int.” denotes “Intermediate,” and “Adv.” denotes “Advanced.”.

**Participant**	1	2	3	4	5	6	7	8
**Timestamp**	08.06 09:20	08.06 09:26	08.06 09:54	08.06 10:58	08.06 14:30	08.06 14:50	08.06 15:45	08.06 17:24
**Role**	Bioinf.	Bioinf.	Bioinf.	Bioinf.	Bioinf.	Bioinf.	Bioinf.	Clinic.
**Programming Experience**	Int.	Adv.	Int.	Int.	Adv.	Adv.	Adv.	None
**Biomedical Experience**	Basic	Adv.	Int.	Basic	Int.	Basic	Adv.	Basic
**Completion**	Yes, without help	Yes, without help	Yes, without help	Yes, without help	Yes, without help	Yes, without help	Yes, without help	Yes, with minor help
**Errors/ Misunder-standings**	No	No	-	No	No	No	No	No
**Q1**	5	5	5	4	5	5	5	4
**Q2**	5	5	5	5	5	5	5	3
**Q3**	4	5	4	4	5	4	5	4
**Q4**	4	5	5	3	5	4	5	4
**Q5**	4	4	4	3	5	5	4	4

**Q6**	5	5	4	5	5	5	5	3

Feedback- What worked well?

Participant 1:
Yes, because you do not have to code and can type everything with regular text
Participant 2:
yes
Participant 3:
I really appreciate that the chat let you review the steps before doing them and warn you if anything is missing. In the use-case I did not understand what [State:] stays for.
Participant 4-6:
-
Participant 7:
nice visualisation and condensed description of research graphs and similarities yes
Participant 8:
Gives direct answer to the question. The structure and explanations could be improved.
